# Tuberculosis revisted: classic imaging findings in childhood

**DOI:** 10.1007/s00247-023-05648-z

**Published:** 2023-05-23

**Authors:** Nasreen Mahomed, Tracy Kilborn, Elsabe Jacoba Smit, Winnie Chiu Wing Chu, Catherine Yee Man Young, Nonceba Koranteng, Joanna Kasznia-Brown, Abbey J. Winant, Edward Y. Lee, Kushaljit Singh Sodhi

**Affiliations:** 1 University of Witwatersrand, 7 York Road Parktown, Johannesburg, 2193 South Africa; 2 Red Cross War Memorial Children’s Hospital, University of Cape Town, Cape Town, South Africa; 3 Department of Imaging & Interventional Radiology, Prince of Wales Hospital, The Chinese University of Hong Kong, Hong Kong, Hong Kong; 4 University of Bristol, Musgrove Park Hospital, Taunton, UK; 5 Department of Radiology, Boston Children’s Hospital and Harvard Medical School, 300 Longwood Avenue, Boston, MA USA; 6 Mallinckrodt Institute of Radiology, Washington University in St. Louis School of Medicine, St. Louis, MO USA; 7 Department of Radiodiagnosis, PGIMER, Chandigarh, India

**Keywords:** Adoloescent, Child, Imaging, Infant, Infection, Radiology, Spina ventosa, Tuberculosis

## Abstract

Tuberculosis (TB) remains one of the major public health threats worldwide, despite improved diagnostic and therapeutic methods. Tuberculosis is one of the main causes of infectious disease in the chest and is associated with substantial morbidity and mortality in paediatric populations, particularly in low- and middle-income countries. Due to the difficulty in obtaining microbiological confirmation of pulmonary TB in children, diagnosis often relies on a combination of clinical and radiological findings. The early diagnosis of central nervous system TB is challenging with presumptive diagnosis heavily reliant on imaging. Brain infection can present as a diffuse exudative basal leptomeningitis or as localised disease (tuberculoma, abscess, cerebritis). Spinal TB may present as radiculomyelitis, spinal tuberculoma or abscess or epidural phlegmon. Musculoskeletal manifestation accounts for 10% of extrapulmonary presentations but is easily overlooked with its insidious clinical course and non-specific imaging findings. Common musculoskeletal manifestations of TB include spondylitis, arthritis and osteomyelitis, while tenosynovitis and bursitis are less common. Abdominal TB presents with a triad of pain, fever and weight loss. Abdominal TB may occur in various forms, as tuberculous lymphadenopathy or peritoneal, gastrointestinal or visceral TB. Chest radiographs should be performed, as approximately 15% to 25% of children with abdominal TB have concomitant pulmonary infection. Urogenital TB is rare in children. This article will review the classic radiological findings in childhood TB in each of the major systems in order of clinical prevalence, namely chest, central nervous system, spine, musculoskeletal, abdomen and genitourinary system.

**Graphical Abstract:**

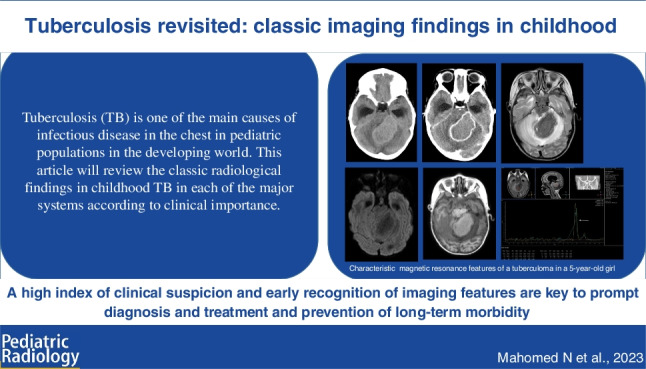

## Introduction

Tuberculosis (TB) remains one of the major public health threats worldwide, causing a high burden of morbidity and mortality, predominantly affecting the African and Asian continents. According to the World Health Organization, an estimated 10 million people were infected in 2020, of which 1.1 million are paediatric patients [1]. This is despite improved diagnostic and therapeutic methods. Tuberculosis continues to be a main cause of infectious disease, especially of the chest, and is associated with substantial morbidity and mortality in paediatric populations, particularly in low- and middle-income countries. Imaging plays an important role in the initial detection and follow-up of TB [2].

### Chest tuberculosis

The diagnosis of pulmonary TB is difficult in children due to non-specific clinical signs and limited utility of the tuberculin skin test, especially in human immunodeficiency virus (HIV)-infected children [3, 4]. Sputum smear microscopy from gastric washings and induced sputa have a low yield [5], with TB culture producing better yield (30% to 40%) but taking a longer time [6–9]. Due to the difficulty in obtaining microbiological confirmation of pulmonary TB in children, diagnosis often relies on a combination of clinical and radiological findings [7, 10, 11]. The main imaging modalities for evaluating thoracic TB in children are chest radiography (CXR), ultrasound (US) and computed tomography (CT). Magnetic resonance imaging (MRI), which is not associated with potentially harmful ionising radiation exposure, can also be considered and is particularly beneficial in children who may require multiple imaging studies, although MRI has limited availability, higher cost and need for sedation in infants and young children [11]. Ultrasound is an imaging modality that can be helpful, particularly for evaluating mediastinal and hilar lymphadenopathy and pleural and pericardial disease [11, 12].

**Fig. 1:**
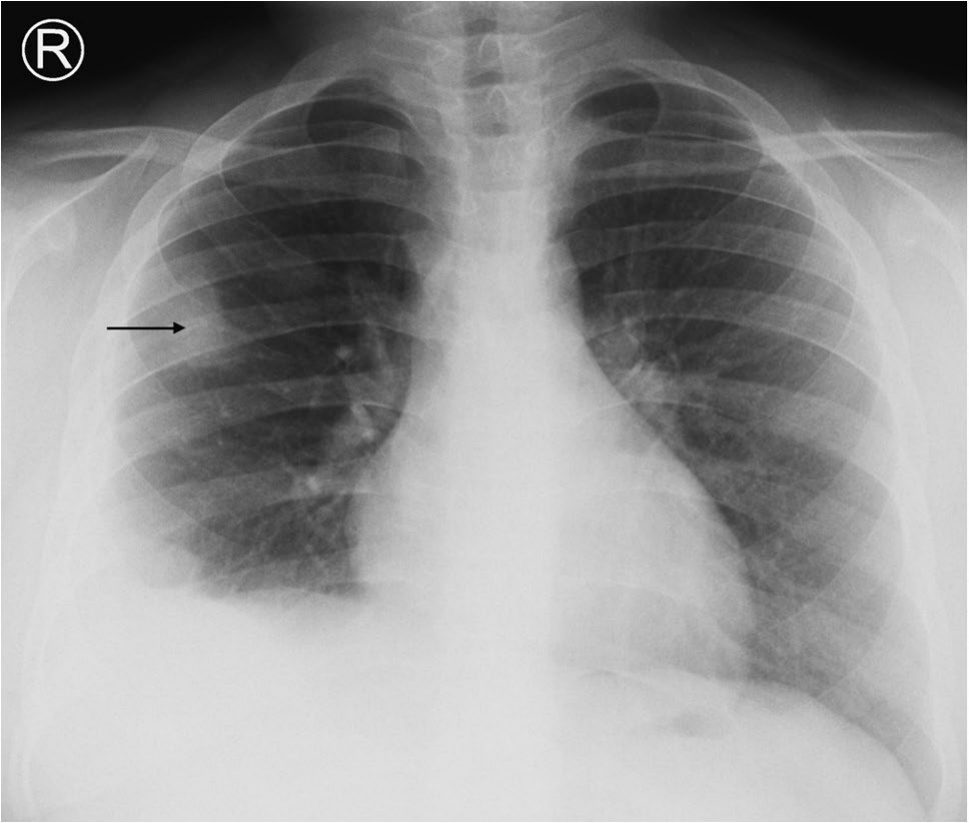
An 8-year-old boy on steroids with cough and night sweats. Posteroanterior chest radiograph shows a focal area of opacification in the right upper lobe representing a Ghon focus (*arrow*) and right pleural effusion. Elevation of the right hemidiaphragm suggests a sub-pulmonic component. Gene expert was positive for pulmonary tuberculosis

**Fig. 2:**
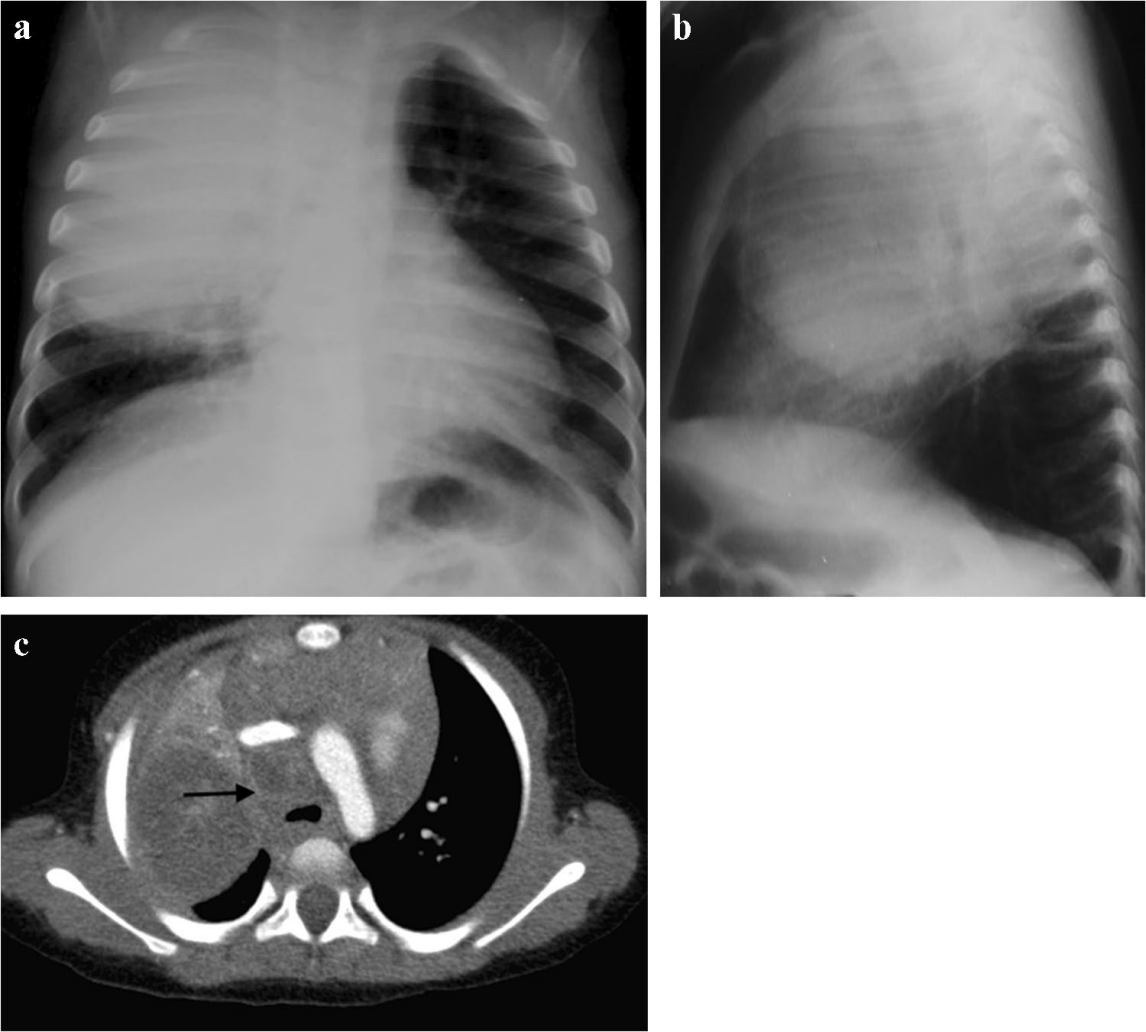
Chest imaging in a 2-year-old girl with pulmonary tuberculosis. **a** Posteroanterior chest radiograph (CXR) shows pneumonia of the right upper lobe. Note the displacement of the trachea towards the left and attenuation of the bronchus intermedius suggesting paratracheal lymphadenopathy. **b** Lateral CXR shows inferior bulging of the horizontal fissure and soft tissue density of lymphadenopathy around the carina. **c** A post-contrast axial computed tomography image (mediastinal window) demonstrates large volume lymphadenopathy of low density and peripherally enhancing lymph nodes (*arrow*). The right upper lobe is expanded and of predominantly low density, consistent with caseous necrosis. There is a small associated pleural effusion

Tuberculosis can affect every organ in the body, but pulmonary infection is the most common. The initial infection, referred to as primary pulmonary TB, is acquired by inhalation of droplet nuclei carrying the organism. The initial focus of parenchymal disease is termed the Ghon focus [13] (Fig. 1). The combination of the Ghon focus and affected lymph nodes is known as the Ranke complex. Air space consolidation is seen in 70% of primary TB without significant lobar predilection [13]. Primary TB may present as pneumonia with lymphadenopathy (Fig. 2).

**Fig. 3:**
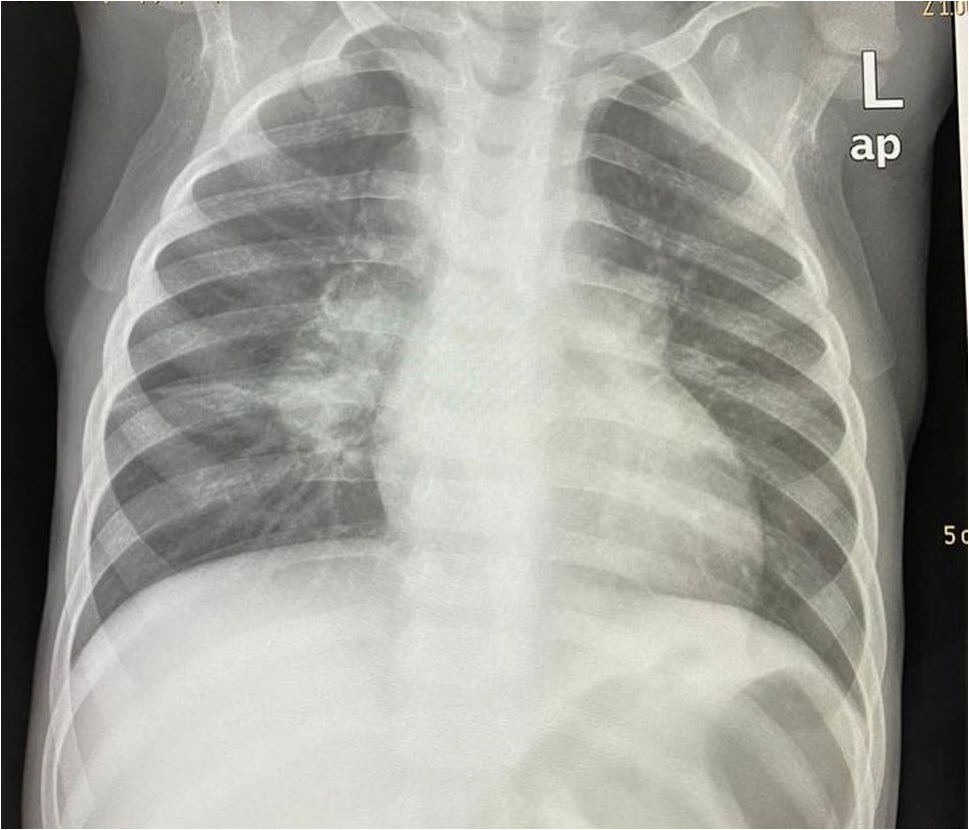
Anteroposterior chest radiograph in a 1-year-old boy with tuberculosis demonstrating bilateral hilar lymphadenopathy

**Fig. 4:**
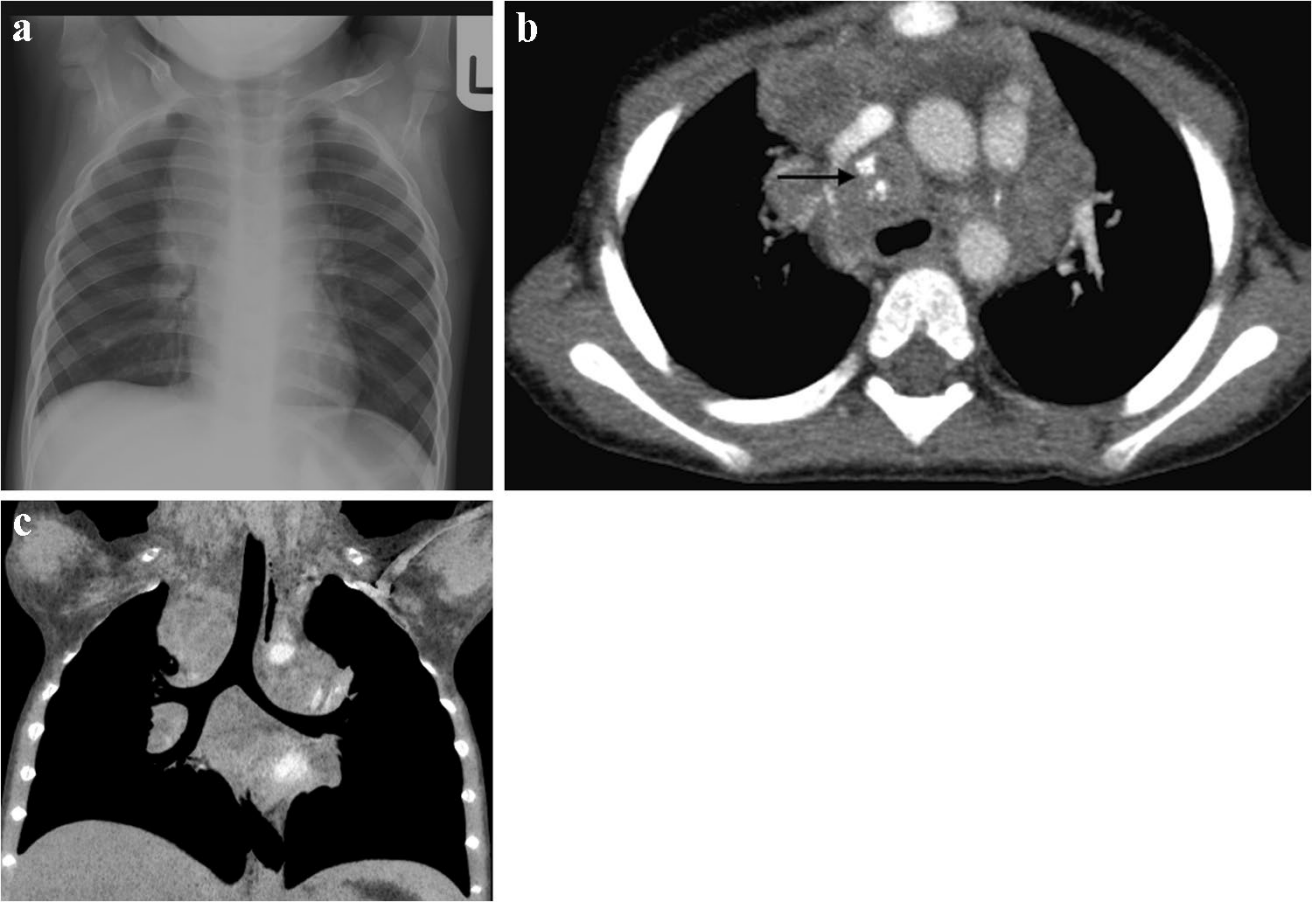
A 1-year-old girl with a cough and known to be a tuberculosis (TB) contact **a** Posteroanterior chest radiograph shows extensive mediastinal and hilar lymphadenopathy. **b** Axial post-contrast computed tomography (mediastinal window) performed a few days later confirms peripherally enhancing and low-density anterior mediastinal, pre-vascular, hilar and paratracheal nodes. There is also a pre-tracheal node that shows punctate calcification (*arrow*). These features are characteristic of TB. **c **Coronal thick slab multiplanar reconstruction (mediastinal window) demonstrates deviation of the trachea to the left but no significant airway compression

**Fig. 5:**
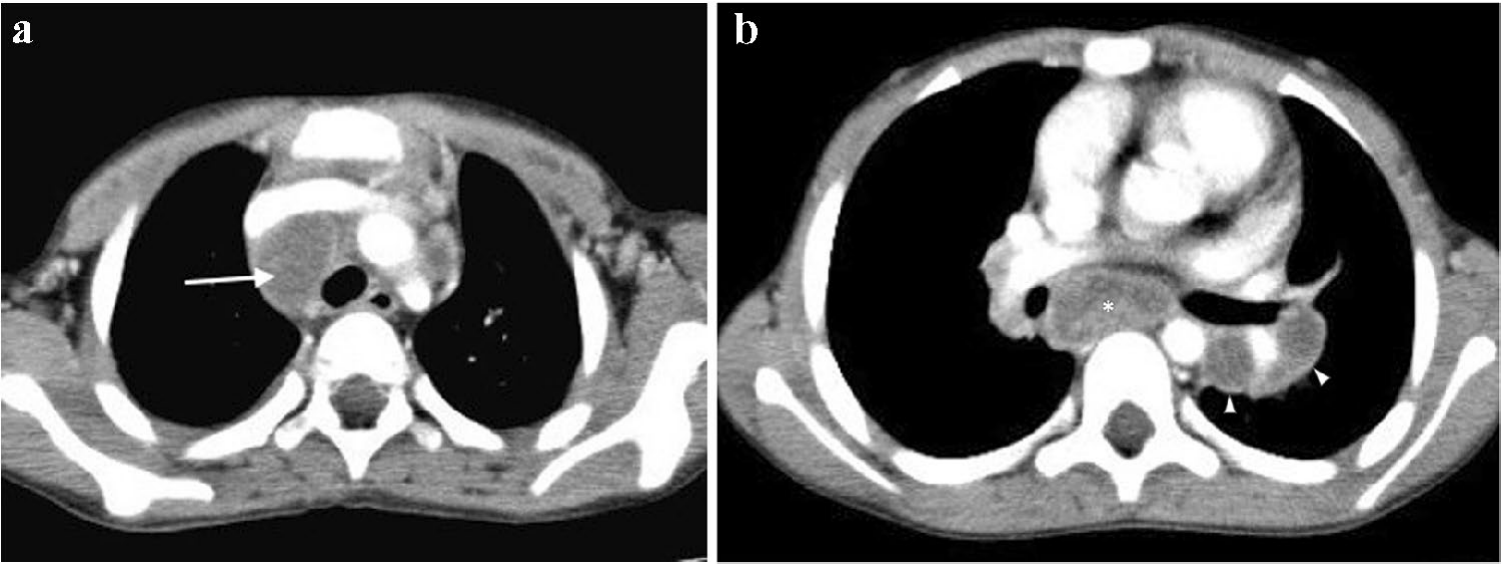
An 8-year-old boy with pulmonary tuberculosis. Axial post-contrast CT (mediastinal window) demonstrates multiple peripherally enhancing necrotic lymph nodes in the mediastinum (*arrow*), left hilum (*arrowheads*) and posterior mediastinum (*asterisk*)

The radiologic hallmark of primary childhood TB is hilar and/or mediastinal lymphadenopathy [10, 13, 14] and this may be the only radiographic finding, prevalent in 46% to 50% of cases of early primary childhood pulmonary TB [13, 15] (Fig. 3). On CXR, lymph node enlargement (typically hilar and/or mediastinal) is more common in children than in adults with primary TB (Fig. 3). On follow-up CXR in children who have had primary TB, calcification is seen within the pulmonary lesion and lymph nodes in 10% to 15% and 5% to 35% of cases, respectively [13, 16] (Fig. 4). An important limitation of reporting intrathoracic lymphadenopathy on CXR in children is the poor inter-reader agreement, as described in the current literature [3, 4, 7, 17]. On post-contrast CT chest, TB lymphadenopathy has a characteristic appearance of a central area of low attenuation surrounded by a rim of contrast enhancement or calcification (15%) and sometimes obliteration of perinodal fat [18] (Fig. 5).

**Fig. 6:**
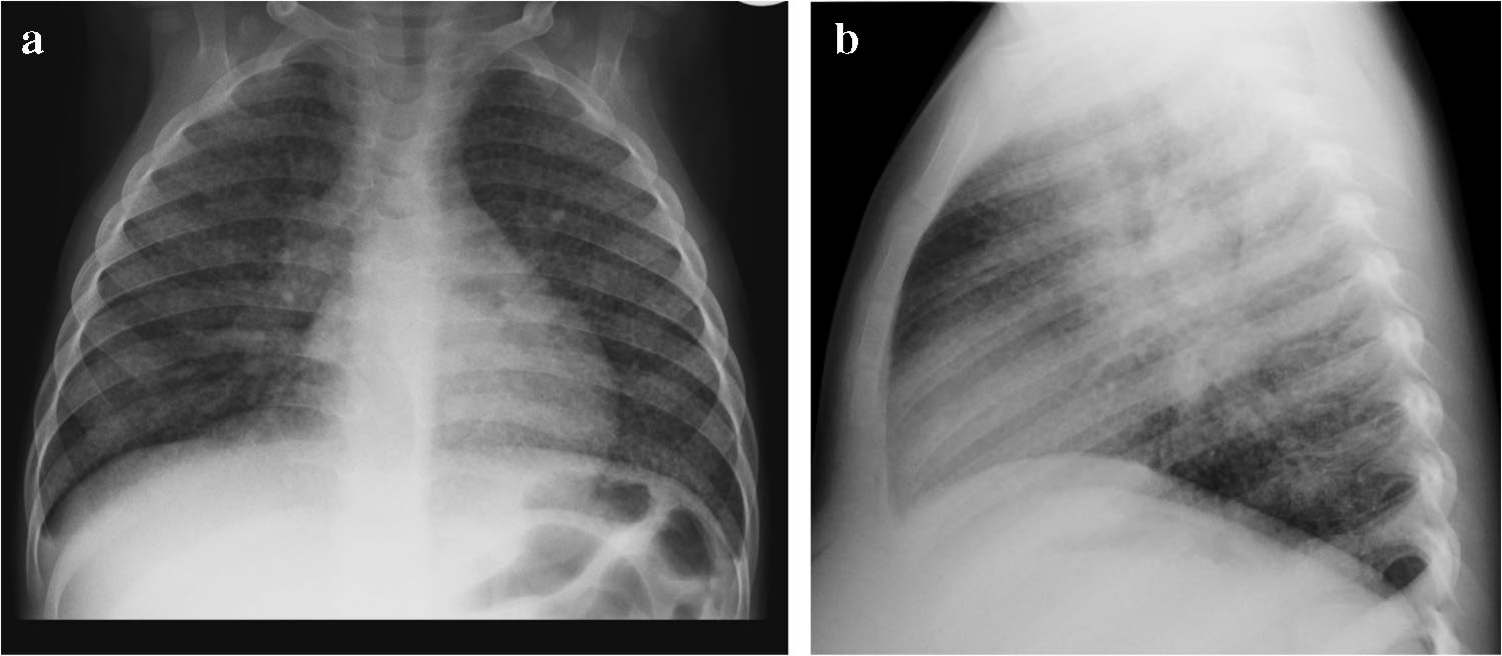
Posteroanterior (**a**) and lateral (**b**) chest radiographs in a 5-year-old boy demonstrate a diffuse pattern of miliary nodules in keeping with haematogenous spread of tuberculosis. Note the leftward displacement of the trachea and prominent right hilum, representing paratracheal and hilar lymphadenopathy

Miliary TB, which is due to haematogenous dissemination, may develop within 6 months of the primary infection, typically in very young and immunocompromised paediatric patients [19–21]. Military TB may also be seen in infants with congenital TB as a result of in utero infection acquired transplacentally from a mother with haematogenous TB or by aspiration of amniotic fluid infected from endometritis or the placenta [19–22]. On imaging studies, miliary TB typically appears as innumerable tiny nodules, usually 1 mm to 2 mm in size, throughout both lungs in a random distribution (Fig. 6). Compared to CXR, miliary TB is better visualised on CT—using maximum intensity projection (MIP) post-processing [23].

**Fig. 7:**
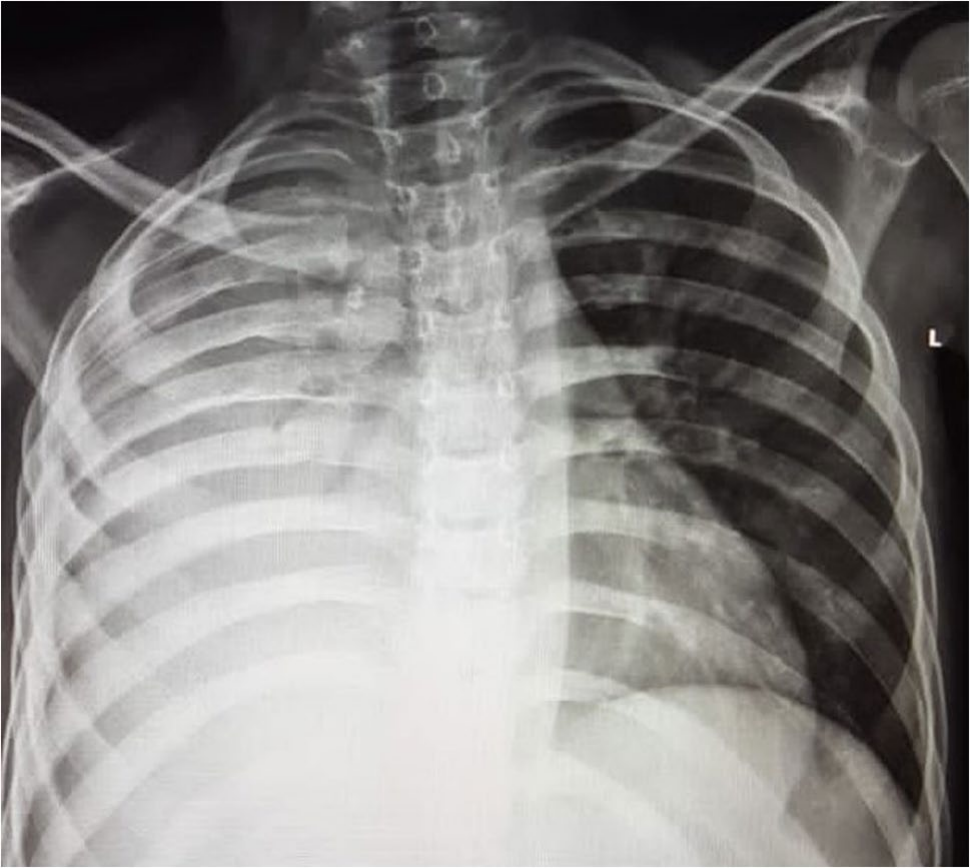
Posteroanterior chest radiograph in a 9-year-old girl with confirmed pulmonary tuberculosis shows a large right-sided pleural effusion with minimum mediastinal shift due to underlying atelectasis

**Fig. 8:**
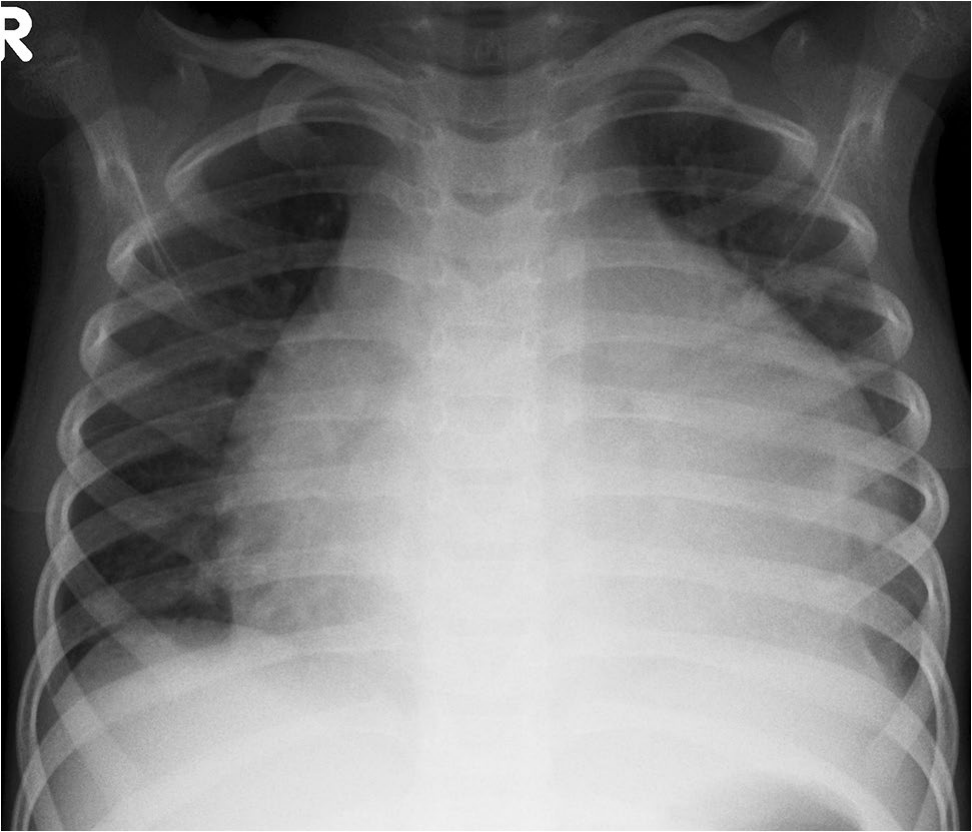
Posteroanterior chest radiograph in a 6-year-old boy demonstrates a tuberculous pericardial effusion  causing globular cardiomegaly

When pulmonary TB affects the pleura, pleural thickening, effusions (Fig. 7) and TB empyema can result as complications with or without pleural calcification. Pericardial effusion and pericarditis may also develop as complications of TB (Fig. 8). These complications can be further evaluated with US or cross-sectional imaging studies such as CT or MRI [11, 24].

On MRI, TB infection typically presents as consolidation with or without cavitation (low signal intensity on T2 and short tau inversion recovery sequences), ground-glass signal abnormality, pulmonary nodules, lymphadenopathy (low signal intensity on T2 and peripheral enhancement on T1 post-contrast sequences) and pleural effusion [25, 26]. A previously published study consisting of 40 consecutive paediatric patients with pulmonary TB showed that MRI with fast imaging sequences is comparable to CXR for evaluating underlying pulmonary consolidation, bronchiectasis, necrosis/abscess and pleural effusion often associated with TB infection in children [27]. It has been reported that MRI may be superior to CT for evaluating lymphadenopathy and pleural effusions, whereas ground-glass and small nodular, particularly tree-in-bud signal abnormalities, are better visualised on CT [25].

Besides lung and mediastinal involvement of TB infection in children, TB can also affect the airways via extrinsic tracheobronchial compression by infectious mediastinal and/or hilar lymphadenopathy or intrinsic airway involvement, which can be seen in up to 43% of children with active TB [28]. On imaging studies, various degrees of lung aeration (from hyperinflation to atelectasis) may be observed, depending on the degree of extrinsic airway compression from adjacent lymphadenopathy—better assessed with cross-sectional imaging studies such as CT or MRI than CXR [11, 28]. The distal trachea and proximal main bronchi are most affected from TB lymphadenopathy. Intrinsic airway involvement is characterised by tracheobronchial thickening and narrowing from mucosal inflammation and oedema. Tracheobronchial fibrostenosis may occur in the later stages and fistula formation may develop with advanced airway disease [28–30].

**Fig. 9:**
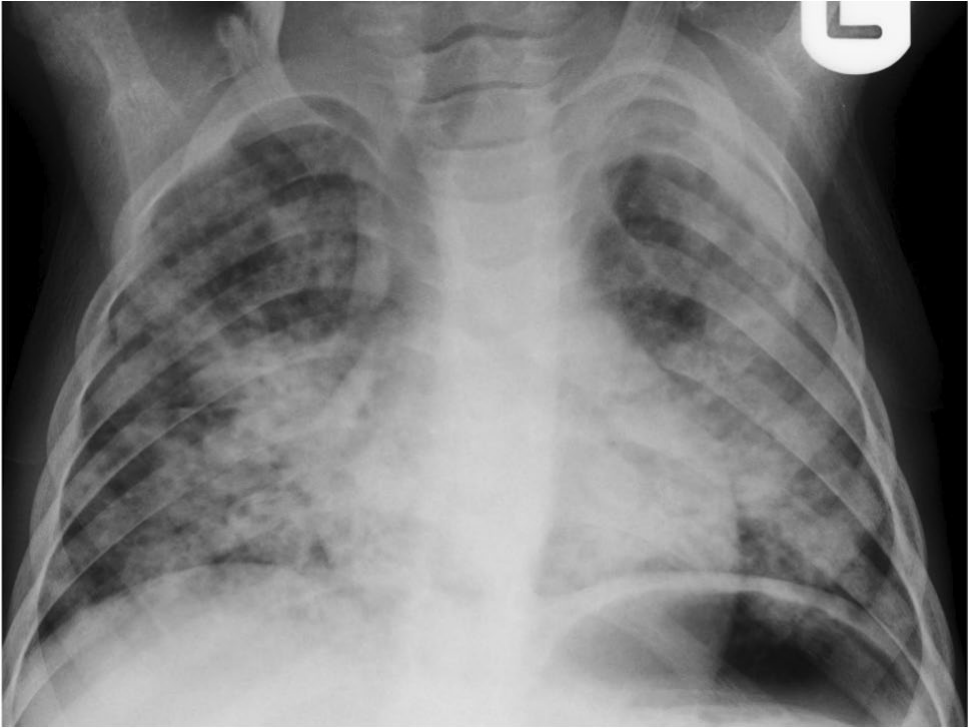
Posteroanterior chest radiograph of an 8-month-old girl with proven tuberculosis (TB) shows multiple ill-defined nodules of differing sizes and parenchymal opacification of the right middle and both lower lobes. These features are suggestive of bronchogenic spread of TB

**Fig. 10:**
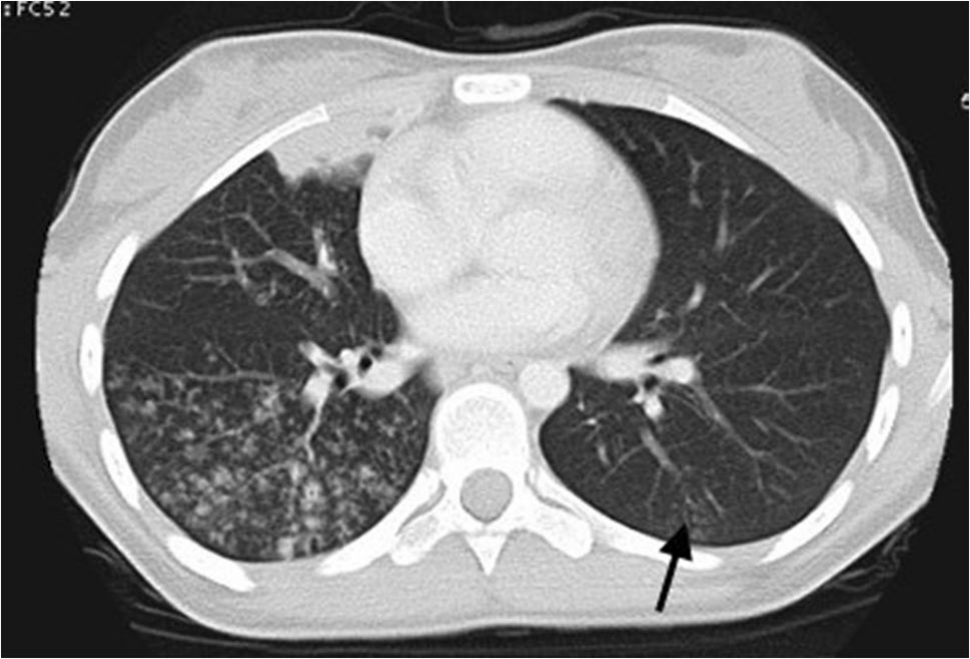
A 12-year-old girl with pulmonary tuberculosis. Axial computed tomography image (lung window) demonstrates multiple coalescing centrilobular nodules in the right lung. A small patch of consolidation is also seen. A few ill-defined centrilobular nodules are also seen in the left lung (*arrow*)

In children with primary TB, infection may progress, children may recover completely without recurrence or following recovery the infection may recur as post-primary TB (also known as reactivation TB or adult-type TB). Post-primary TB is uncommon in children. However, when it occurs, it is typically seen in children over 10 years of age. Post-primary TB results from the growth of previously dormant bacilli either at the site of the initial Ghon focus or elsewhere in the lung [31]. Post-primary TB has a classic predilection for the posterior segments of the upper lobes and the superior segments of the lower lobes. It typically presents as areas of consolidation with ill-defined margins often with small satellite foci in the adjacent lung. These areas of consolidation can be necrotic and cavitary. The cavities often have irregular thin or thick walls with air-fluid levels and may be colonised by fungal infection such as aspergillus species [13]. Post-primary TB can also be associated with nodular and linear fibrosis and distortion of the adjacent mediastinal and bronchovascular structures. In addition, small centrilobular nodules with a linear branching pattern known as "tree-in-bud" appearance can also be seen in paediatric patients with endobronchial spread of post-primary TB [32–34] (Figs. 9 and 10). Pleural effusion is a common accompanying imaging feature. Unlike in primary TB, lymphadenopathy is rarely seen in post-primary TB. In some recovered paediatric patients, bronchiectasis may be identified on follow-up imaging studies [35].

**Fig. 11:**
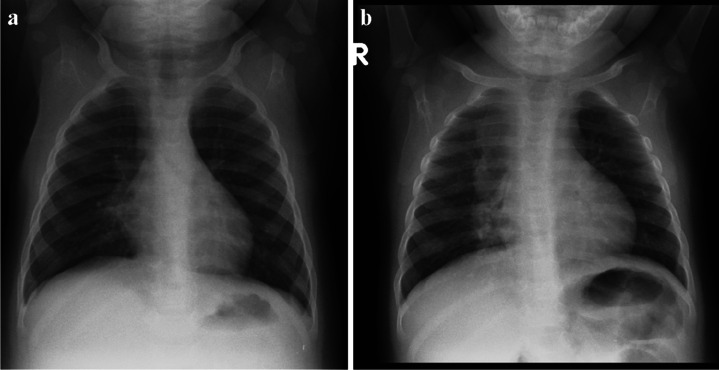
A 3-year-old boy newly diagnosed with human immunodeficiency virus infection and started on antiretroviral therapy. **a** Posteroanterior chest radiograph (CXR) on initiation of therapy shows no radiographic evidence of pulmonary tuberculosis (TB) and the patient was asymptomatic. **b** Repeat posteroanterior CXR 6 weeks later, on presentation with a cough, shows marked paratracheal, right hilar and subcarinal lymphadenopathy with splaying of the carina and attenuation of the bronchus intermedius and middle/lower lobe bronchi in keeping with TB. This is an example of unmasking TB immune reconstitution inflammatory syndrome

Immune reconstitution inflammatory syndrome is an inflammatory disorder related to infectious processes, that manifests after the initiation of antiretroviral therapy and can be classified as unmasking or paradoxical [36]. The prevalence of immune reconstitution inflammatory syndrome in children has been reported as 4.8% among 104 children under 8 years of age [37]. Approximately half of all cases of immune reconstitution inflammatory syndrome in the chest are associated with *Mycobacterium tuberculosis*. The most common radiological manifestations of TB immune reconstitution inflammatory syndrome occur within the chest and include new or worsening hilar or mediastinal lymphadenopathy, with or without tracheo-bronchial compression (Fig. 11). Other common CXR findings include worsening or new air space consolidation, pleural effusions and reticular or nodular infiltrates [36].

### Central nervous system tuberculosis

Tuberculous meningitis continues to be an important cause of morbidity, with one in five affected children dying and only one in three surviving without disability [38]. Depending on disease prevalence and age range studied, TB meningitis accounts for 1% to 10% of childhood TB cases, although there is considerable uncertainty around these estimates, given the difficulties in making a microbiological diagnosis [38]. Diagnostic and treatment delay are the most important predictors of mortality and disability [39]. Diagnostic challenges include non-specific symptoms leading to misdiagnosis, lack of sensitive diagnostic tools to confirm TB meningitis, suboptimal sample collection for laboratory processing and lack of standardised region-specific training of healthcare staff to identify TB meningitis and initiate appropriate anti-TB therapy [38]. Currently, a definitive diagnosis of TB meningitis requires a positive cerebrospinal fluid (CSF) culture (2–6 weeks to yield a result) or visualisation of acid-fast bacilli on CSF. The rapid molecular test, Xpert *Mycobacterium tuberculosi*s/rifampicin (MTB/RIF), is an important diagnostic test for all forms of TB, and next-generation Xpert MTB/RIF Ultra (Xpert Ultra) has a sensitivity of 44% to 77% and specificity approaching 100% in CSF of adults with TB meningitis [40, 41].

Information on diagnostic performance in the paediatric population and impact on mortality have yet to be clearly defined; therefore, diagnosis of TB meningitis still relies on clinical symptoms and signs; CSF parameters (lymphocytic pleocytosis, increased protein and decreased glucose); and radiology, namely CXR and neuroimaging. In most centres, CT is used for acute imaging as it is accessible and can rapidly demonstrate complications such as hydrocephalus that require urgent neurosurgical intervention. CT is also used to monitor response to treatment; however, it should be used judiciously as multiple scans subject patients to a significant radiation burden. Post-contrast MRI has become part of routine care in many centres as it avoids radiation and is more sensitive in detecting the number and location of infarcts and providing prognosis [42–45]. MRI also better detects basal meningeal, pituitary stalk and cranial nerve enhancement [44, 46].

**Fig. 12:**
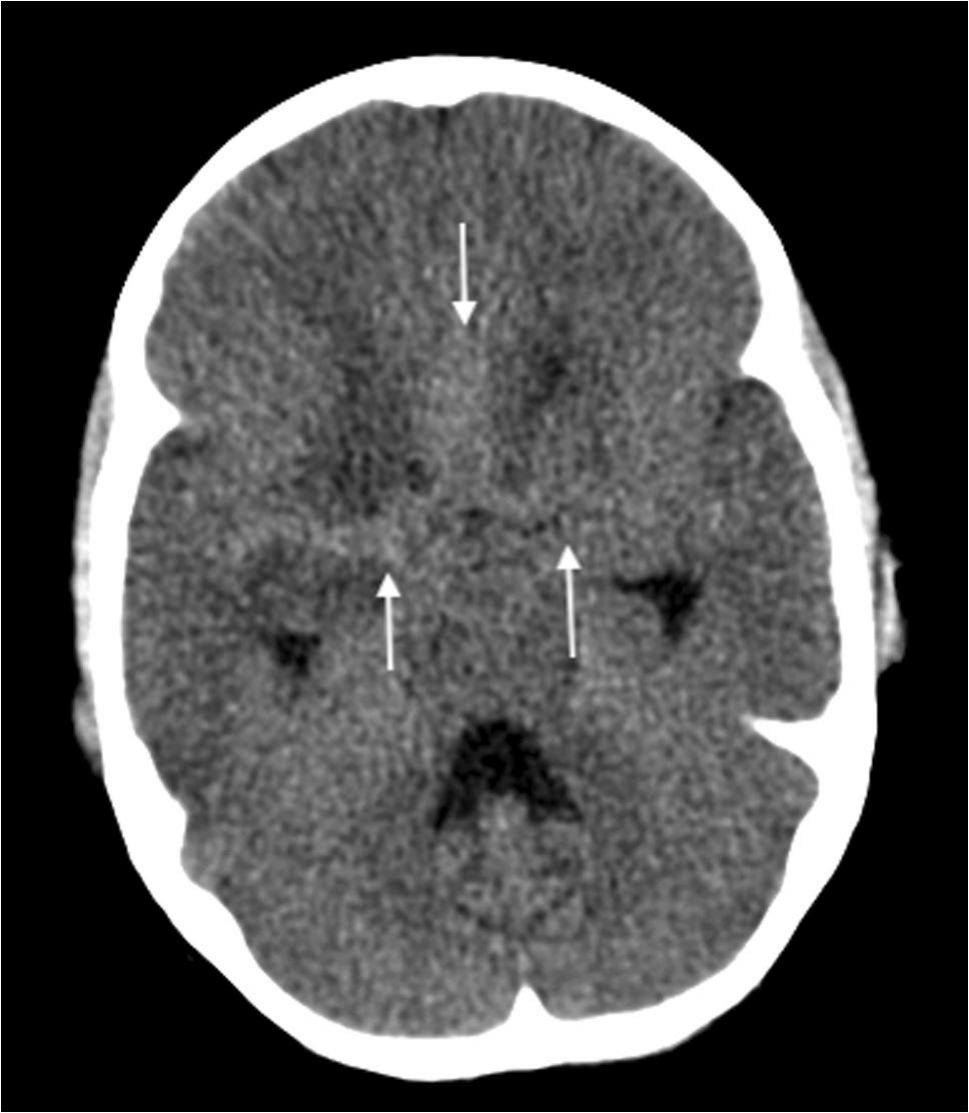
Non-contrast axial computed tomography of the brain in a 30-month-old boy with tuberculosis demonstrates hyperdensity (*arrows*) in the basal cisterns, representing granulation tissue and exudate. Note the bilateral basal ganglia and right temporal lobe low density, indicating established infarcts

**Fig. 13:**
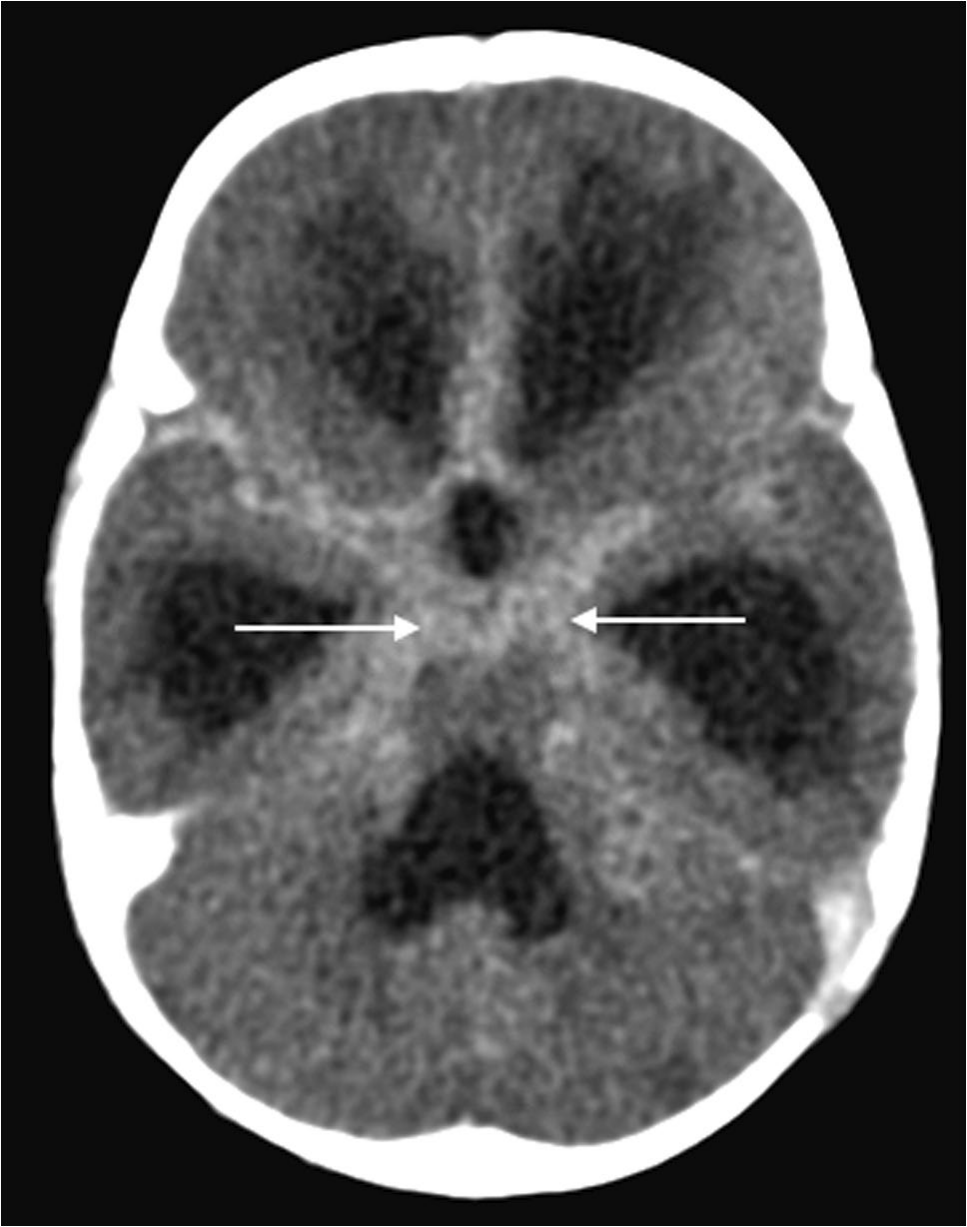
Axial post-contrast  computed tomography image of the brain in a 30-month-old boy with tuberculosis (same patient as Fig. 12) shows diffuse basal meningeal enhancement filling the cisterns (*arrows*) and accompanying hydrocephalus

**Fig. 14:**
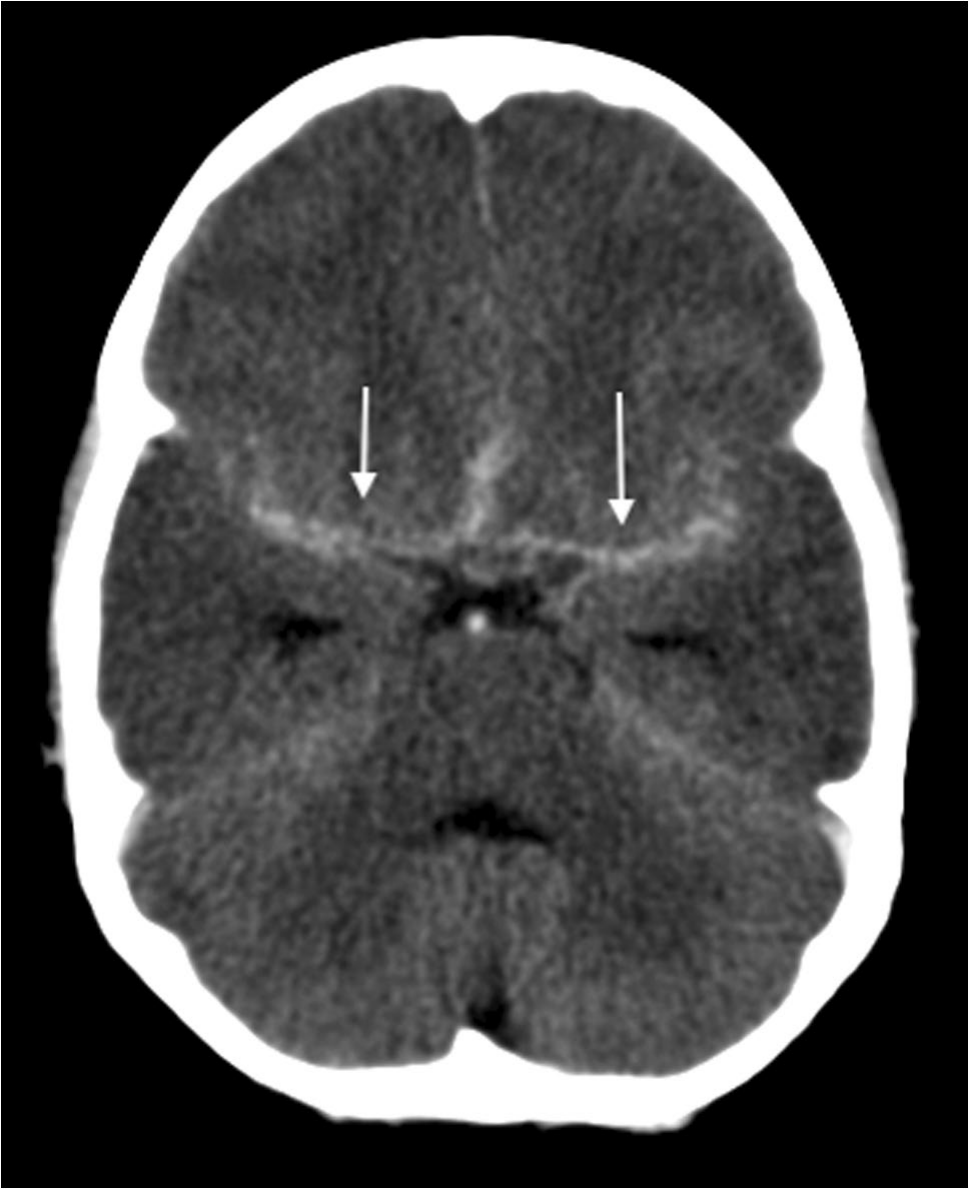
Axial post-contrast computed tomography image of the brain in a 30-month-old boy with tuberculosis (same patient as Figs. 12 and 13) shows enhancement at the junction of the suprasellar and middlecerebral artery cisterns (the “Y-sign”) (*arrows*)

**Fig. 15:**
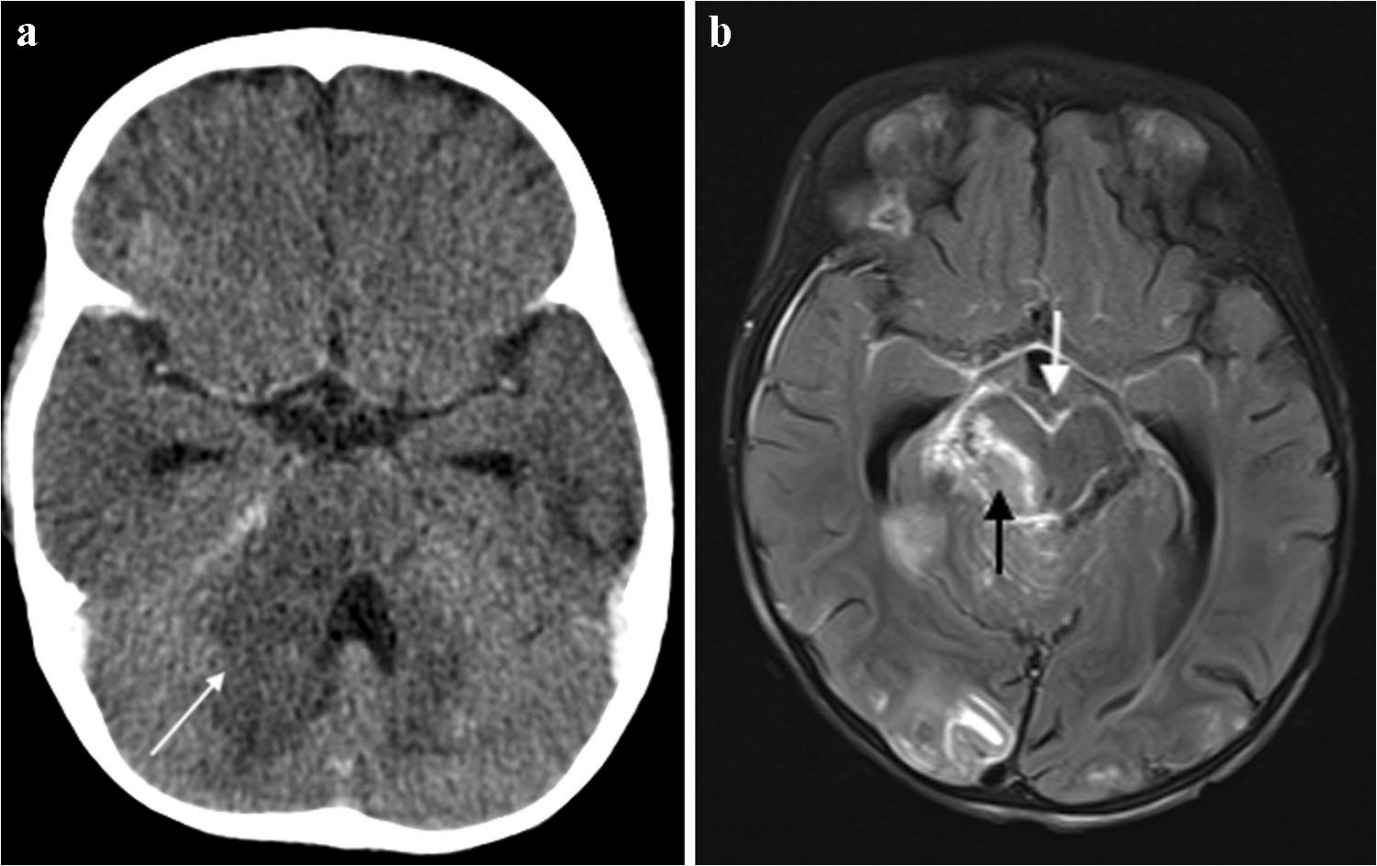
**a** Brain imaging in an 18-month-old girl with newly diagnosed human immunodeficiency virus infection and presumed tuberculous meningitis. **a** Axial post-contrast computed tomography shows hypodensity with mass effect in the right cerebellar peduncle (*arrow*), hydrocephalus with temporal horn prominence and absence of basal meningeal enhancement. **b** Axial T1 post-gadolinium magnetic resonance image clearly demonstrates basal meningeal enhancement, an enhancing mass in the right peduncle (*white arrow*) and multiple tiny miliary nodules (*black arrow*)

The triad of neuroradiological findings in TB meningitis is basal meningeal enhancement, hydrocephalus and infarction. On pre-contrast CT, hyperdensity can be seen in the cisterns in approximately 50% of patients—this “hyperdense cistern sign” is reported to be specific for TB meningitis and the hyperdense tissue is seen to enhance with contrast [47] (Fig. 12). The reported incidence of basal meningeal enhancement is 75% to 92% and represents meningeal inflammation, an enhancing gelatinous exudate and/or granulation tissue in the subarachnoid space [48]. Enhancement is best demonstrated on post-contrast CT or T1 fluid-attenuated inversion recovery(FLAIR) MRI scans, with the typical pattern considered one of the features most sensitive for the diagnosis of TB meningitis [48]. Enhancement is seen in the basal cisterns, in the tentorium, around the optic chiasm, the cranial nerves, over the cerebral convexities and (with extension of disease into the ventricular system) in the ependyma and choroid plexus [48, 49] (Fig. 13). MRI is more sensitive than CT in detecting basal enhancement because it is not confused with blood vessels (seen as flow voids); on CT, vessels and subtle enhancement of the edges of the cisterns can be indistinguishable [50]. Objective criteria for evaluating basal meningeal enhancement on CT have been proposed with the most useful being enhancement at the junction of the suprasellar and middle cerebral artery cistern (the “Y-sign”) and linear enhancement in the middle cerebral artery cistern, contrast filling the cistern and asymmetry [47] (Fig. 14). Asymmetrical or focal enhancement may be seen in 10% of cases, involving the Sylvian fissure and middle cerebral artery cistern [46]. Minimal, asymmetric or absent enhancement can be seen in HIV-infected children with TB meningitis. Nodular enhancement is more common, likely due to immunosuppression in HIV-positive patients leading to an impaired granulomatous response to mycobacteria [51, 52]*.* In HIV-positive patients, MRI is recommended to assist in early and accurate diagnosis [53] (Fig. 15). The evolution of enhancement in TB meningitis is variable—some follow-up scans show improvement or no change; however, up to 28% of scans may demonstrate new or progressive enhancement after the first month of treatment [54, 55].

**Fig. 16:**
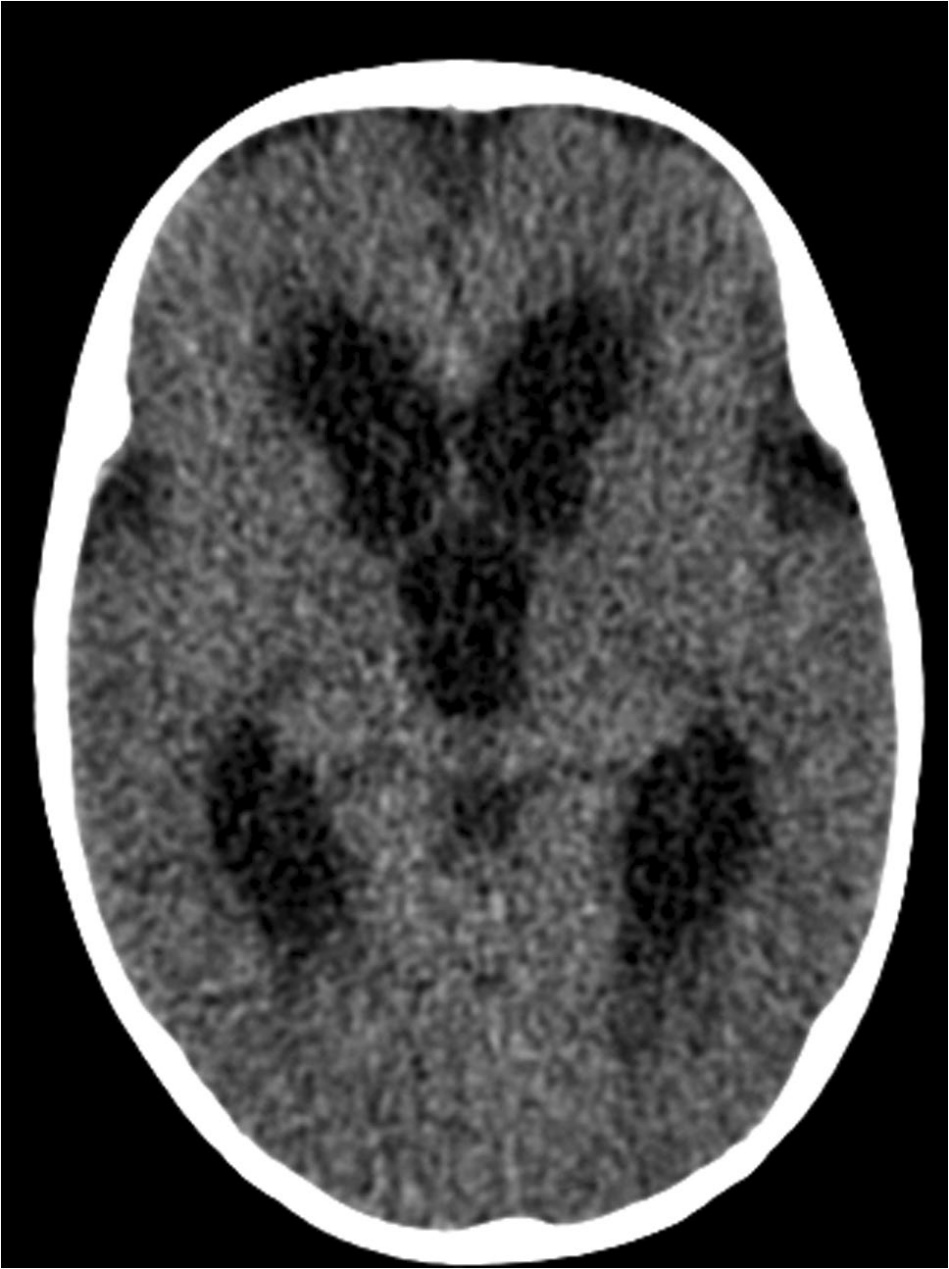
Axial non-contrast computed tomography scan of the brain in a 2-year-old boy with severe hydrocephalus due to tuberculous meningitis

**Fig. 17:**
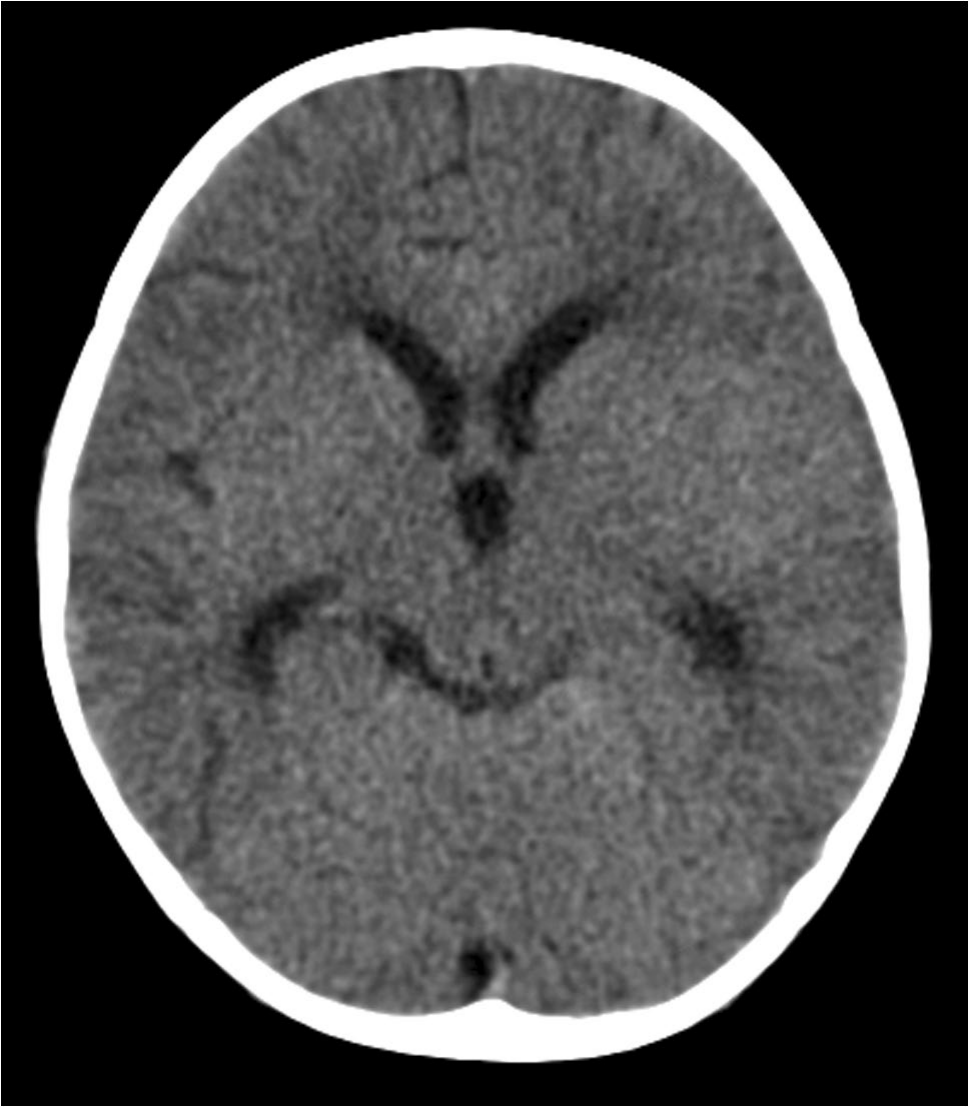
Axial non-contrast computed tomography of the  brain in a 4-year-and-6-month-old boy with tuberculous meningitis. There is only mild hydrocephalus despite the child being severely obtunded at presentation

Hydrocephalus occurs in up to 80% of patients with TB meningitis and is more common and severe in children [49]. Hydrocephalus is of two forms, communicating hydrocephalus if the obstruction to CSF flow is in the subarachnoid space (as a result of the exudate in the basal cisterns) and non-communicating hydrocephalus when the obstruction occurs at the outlet foramina of the fourth ventricle, or cerebral aqueduct (by circumferential compression of meningeal exudates, mesencephalic oedema or intraventricular exudate) [56]. Occasionally, hydrocephalus results from obstruction of the foramen of Munro [57]. Non-communicating hydrocephalus occurs less frequently (12% to 25%) (Fig. 16) but accurately differentiating between the two forms is not possible on brain imaging [56, 58, 59]. Diagnosing raised intracranial pressure in children with TB meningitis can be challenging as there is poor correlation between the degree of hydrocephalus (ventricular size) and the severity of the raised intracranial pressure (Fig. 17). Communicating hydrocephalus may progress to non-communicating hydrocephalus, requiring ventricular drainage [60].

**Fig. 18:**
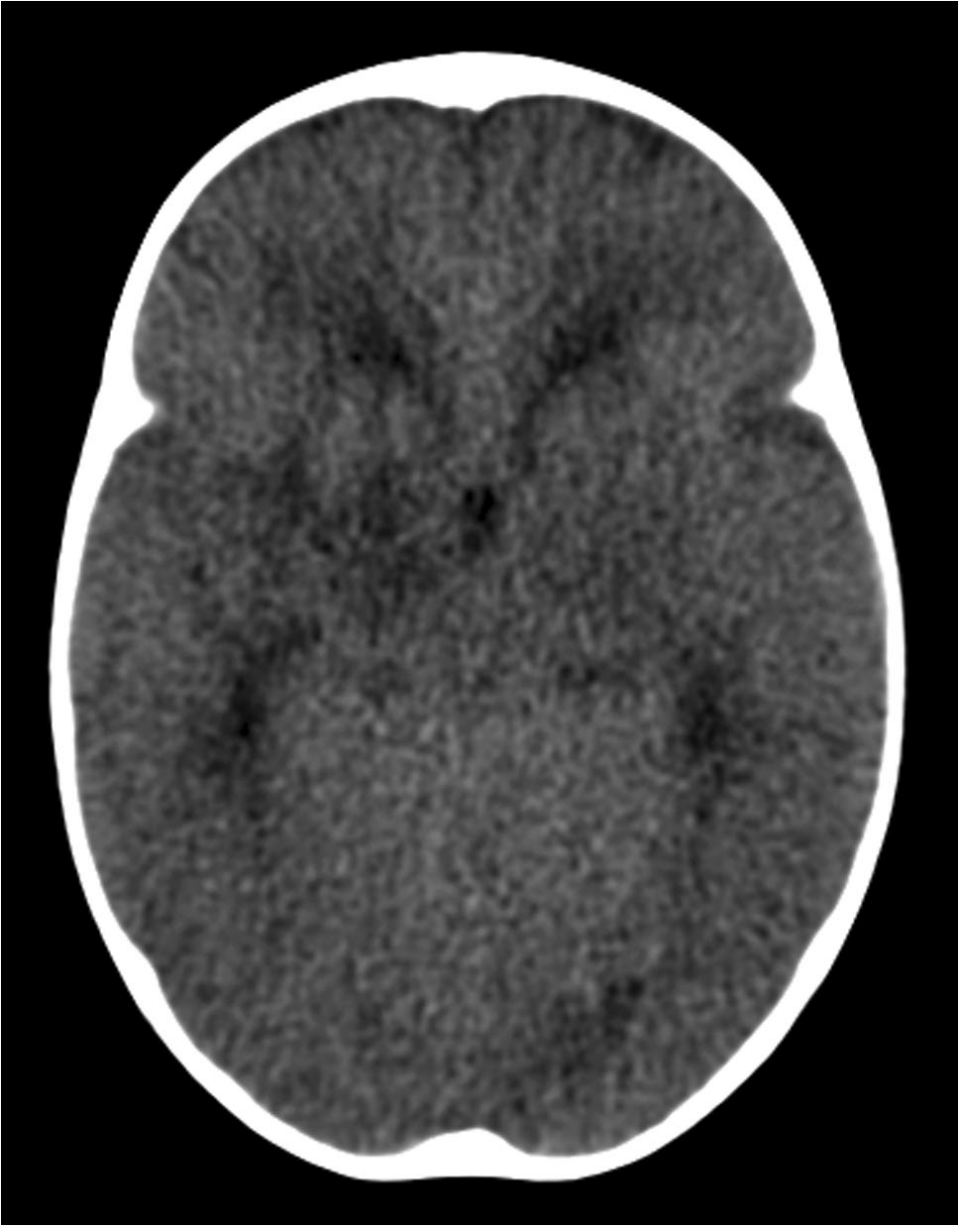
Axial non-contrast computed tomography of the brain in a 7-year-old boy with tuberculosis shows infarcts in the “TB zone” of the bilateral medial lenticulostriate and thalamo-perforating vessels (head of the caudate nucleus, anterior limb of the internal capsule and the anteromedial thalamus)

**Fig. 19:**
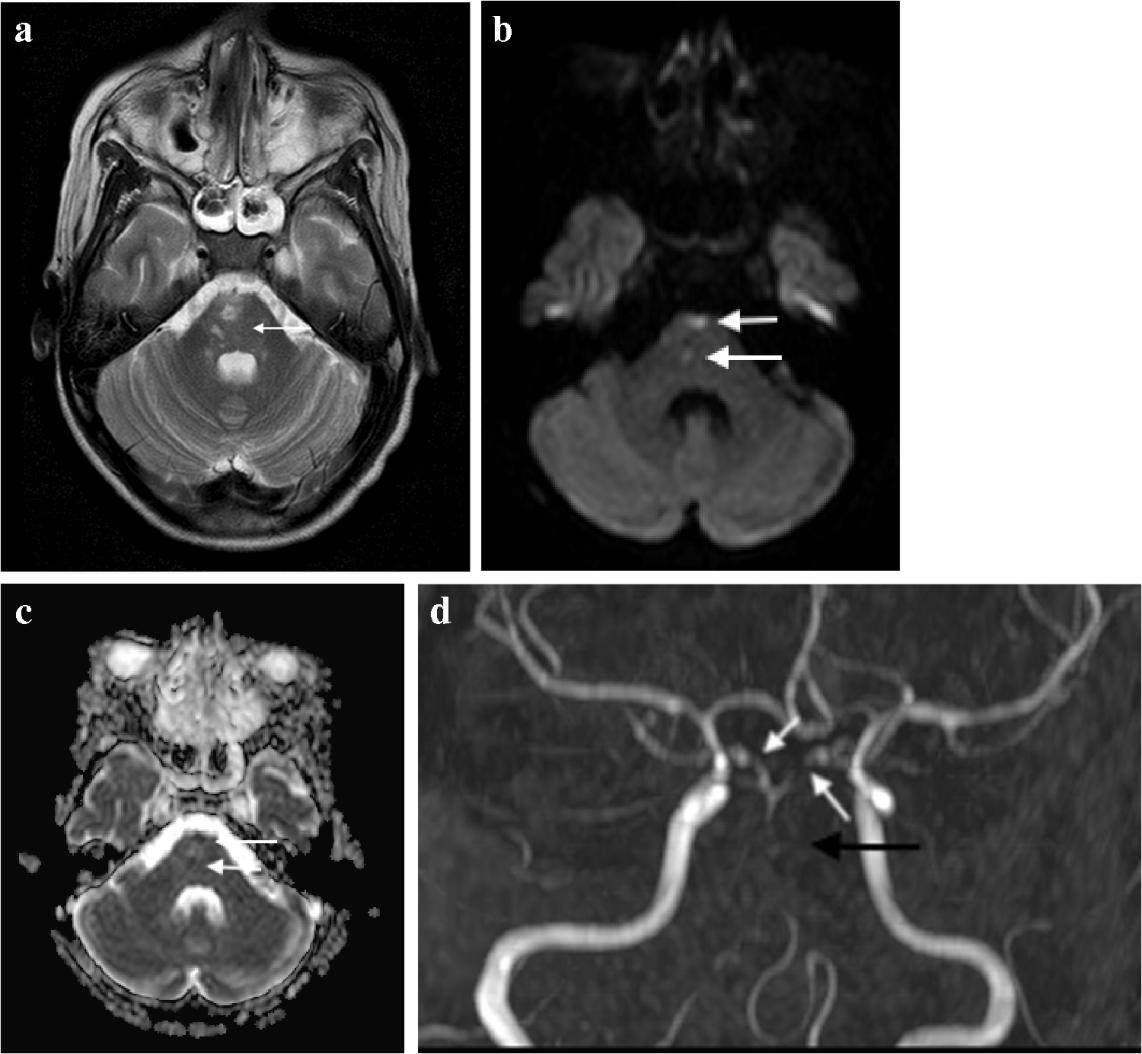
Magnetic resonance imaging of the brain in a 12-year-old boy with tuberculous meningitis and persistent decreased level of consciousness. **a** Axial T2 demonstrates multiple hyperintense foci in the pons (*arrow*). **b** Axial diffusion-weighted image demonstrates restricted diffusion (*arrow*) **c** Low signal (*arrows*) on the apparent diffusion coefficient map confirms infarcts. **d** Coronal magnetic resonance angiogram image shows partial occlusion of the basilar artery (*black arrow*) and irregularity of both posterior communicating arteries (*white arrows*)

The poor outcome from TB meningitis can be linked to the extent of ischaemic brain injury, with antituberculosis treatment and steroids being ineffective at preventing vascular complications [57]. Approximately one third of all children with advanced TB meningitis will have an ischaemic event [61]. Periarteritis of the cerebral vessels (which become surrounded by inflammatory exudate) progresses to panarteritis and vascular occlusion predominantly in the middle cerebral artery territory and involving the so-called "TB zone" of the medial lenticulostriate and thalamo-perforating vessels [57]. This results in infarcts of the head of the caudate nucleus, anterior limb of the internal capsule and the anteromedial thalamus (Fig. 18). The large vascular distribution territories of the middle and anterior cerebral arteries are affected less often [49]. Infarcts in the cerebellum and midbrain have been reported in up to 47% of children with TB meningitis, likely due to exudate coating the basilar artery and its branches; these are associated with a more severe presentation and poorer outcome [43] (Fig. 19)*.* Border zone necrosis develops because of inflammation of the brain underlying the exudate, with extension of disease along small proliferating vessels into the brain parenchyma resulting in vasculitis and ischaemic change. These infarcts are adjacent to areas of severe meningeal/cisternal inflammation [45].

Infarcts are frequently poorly demonstrated on admission CT scans with MRI being more sensitive in the early phase [62]. Infarcts not visualised on initial imaging are identified on follow-up scans in 22% to 48% of patients, suggesting that the disease process is ongoing and the injury evolving, despite treatment [54, 55, 63]. Post-contrast MRI is superior in detecting early ischaemia, particularly in the posterior fossa and basal ganglia. Vascular abnormalities detected on MRI and CT angiograms in 46% to 70% of TB meningitis patients include paucity of terminal branches, irregular beading, segmental attenuation of major vessels and complete occlusion [62–65] (Fig. 19).

**Fig. 20:**
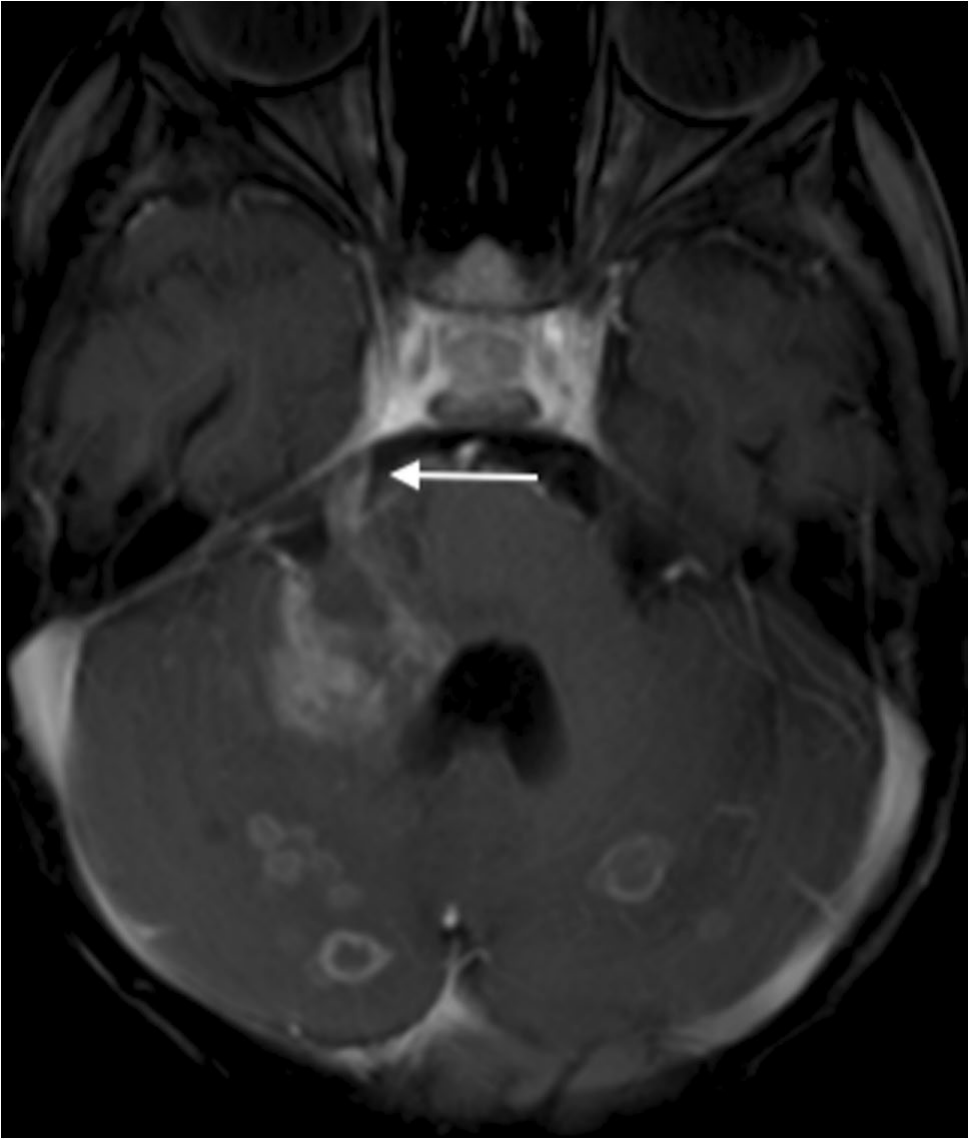
Axial T1 post-gadolinium magnetic resonance image in a 3-year-and-4-month old girl shows enhancement of the right trigeminal nerve (*arrow*). There is enhancement of the right cerebellar peduncle and there are multiple peripherally enhancing tuberculomas

In addition to the triad of basal meningeal enhancement, hydrocephalus and infarction, there are other manifestations of TB meningitis that should be looked for on imaging. Cranial nerve enhancement is seen in 70% of patients with cranial nerves II, III, IV and VII being the most frequently affected [49, 50]. When associated with cranial nerve palsy, this is thought to be due to entrapment of the nerve in basal exudates or vascular compromise resulting in nerve ischaemia; late-stage fibrosis can cause permanent loss of function [49] (Fig. 20). Inflammation and granulation tissue is most prominent in the suprasellar cistern, surrounding the pituitary gland and hypothalamus. It is thought that this inflammation may be the cause of the syndrome of inappropriate anti-diuretic hormone secretion/diabetes insipidus that is seen in up to 70% of children [66]. Meningeal, parenchymal and ependymal tuberculoma formation can be seen in 15% of children with TB meningitis. Potential sequelae of TB meningitis include meningeal or parenchymal calcification and focal areas of atrophy/encephalomalacia in areas of infarction with ex-vacuo dilatation of ventricles.

The differential diagnosis of TB meningitis includes other infectious disease (e.g. non-tuberculous, viral and fungal) and non-infectious leptomeningeal conditions such as sarcoidosis and meningeal carcinomatosis (e.g. medulloblastoma, pineoblastoma) [50]. Pachymeningeal TB refers to either isolated dural involvement or a predominantly dural-based lesion with secondary pial or parenchymal involvement that can be focal or diffuse and is uncommon [49]. Lesions appear hyperdense on CT, isointense on T1 and iso- to hypointense on T2 and show contrast enhancement [67].

**Fig. 21:**
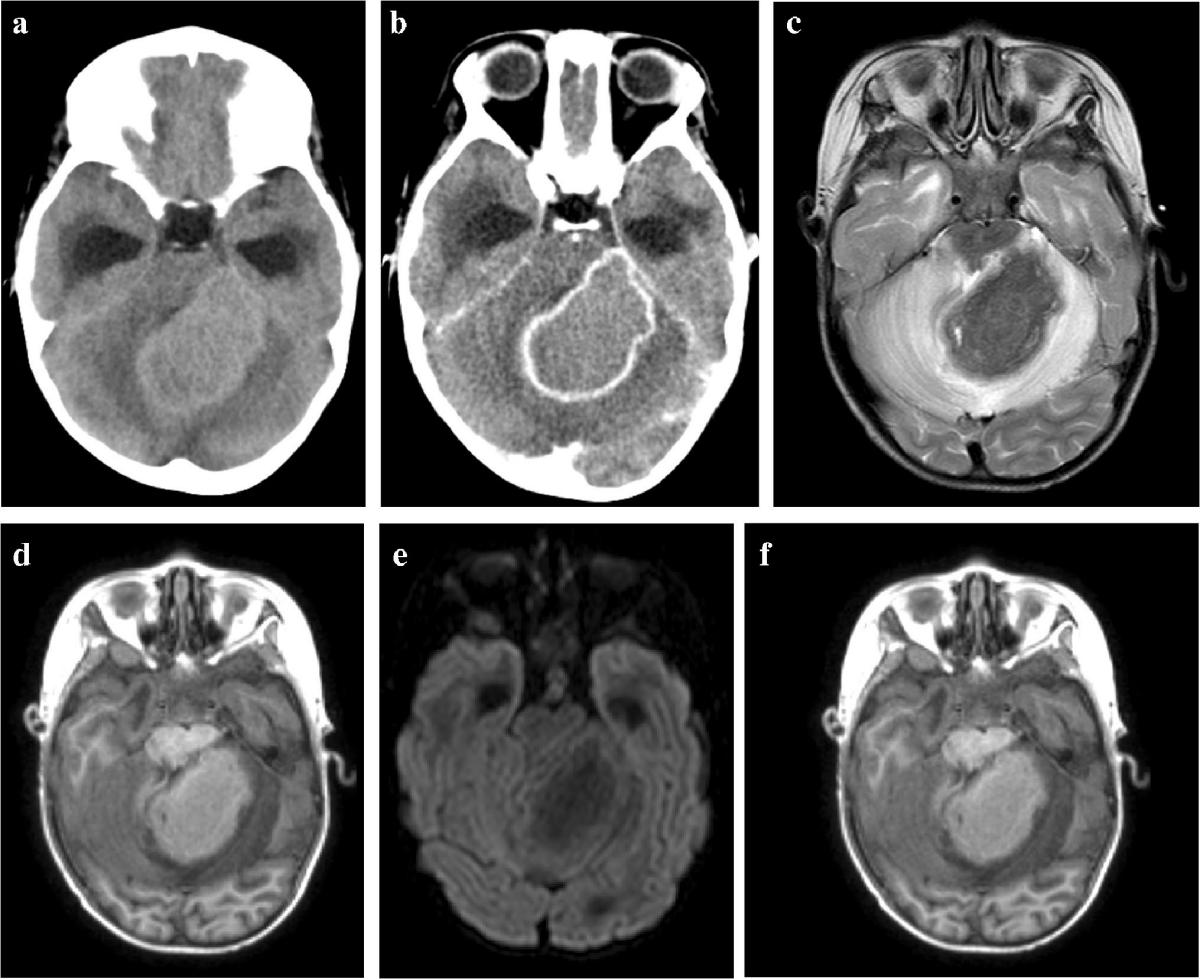
A 5-year-old girl with headaches and vomiting referred with a posterior fossa mass. **a**, **b** Pre- (**a**) and post-contrast (**b**) axial computed tomography images show an intermediate density mass with peripheral enhancement involving the left cerebellar peduncle and cerebellar hemisphere and complicated by hydrocephalus. **c**, **d** Axial magnetic resonance images (MRI). The mass is profoundly hypointense on T2 (**c**) and shows no diffusion restriction (**d**). **e** Magnetic resonance spectroscopy shows  a lactate peak (*arrow*). **f **Axial post-gadolinium T1 MRI shows central intermediate intensity and peripheral enhancement. These imaging features are characteristic of a tuberculoma—there was response to anti-tuberculous treatment

The pathophysiology of tuberculoma formation is complex, with the host’s immune cells (particularly macrophages) targeted by *Mycobacterium tuberculosis* [68]. The host immune response eventually slows down bacterial growth with granuloma formation around infected and necrotic macrophages, although bacteria can persist within these granulomas for years [69]. Most tuberculomas occur at the grey/white matter junction, supporting the hypothesis of haematogenous spread, with a small number developing from extension of CSF infection into adjacent parenchyma via cortical veins or perivascular Virchow-Robin spaces [49]. Pathologically, the tuberculoma is composed of a central zone of solid caseous necrosis containing TB bacilli, surrounded by a capsule of collagenous tissue, giant cells and inflammatory cells. Outside the capsule, there is oedema and astrocyte proliferation [70]. Tuberculomas can involve the brain, spinal cord, subarachnoid, subdural or epidural space [49]. They may be single or multiple with sizes varying from 1mm to 80 mm [36] (Fig. 21). Infratentorial tuberculomas are more common in children than adults [70].

**Fig. 22:**
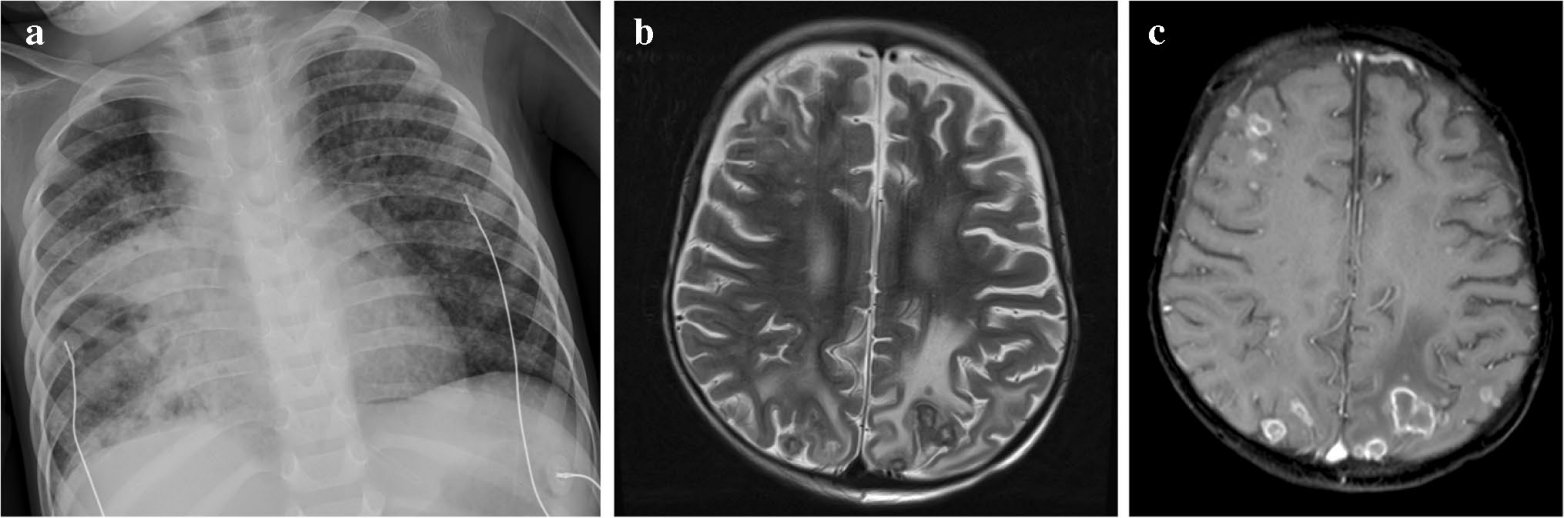
** A** 9-month-old boy presenting with a decreased level of consciousness. **a** Posteroanterior chest radiograph shows paratracheal and hilar lymphadenopathy and a diffuse micronodular pattern in keeping with miliary tuberculosis. **b, c** Axial T2 (**b**) and T1 post-gadolinium (**c**) magnetic resonance imaging  show innumerable lesions at the grey/white matter junction and meninges, some of which are hypointense (caseating tuberculomas) and some of which have T2 hyperintense centres (liquifying caseating tuberculomas) and show surrounding oedema (**b**). There is peripheral enhancement on the T1 post-gadolinium image (**c**)

Miliary central nervous system tuberculomas are usually associated with TB meningitis and many affected paediatric patients have miliary TB on chest imaging [50] (Fig. 22). The clinical presentation is more subtle than that of TB meningitis and depends on the location and size; a focal seizure in an otherwise normal child is the most common presentation in TB endemic areas [57]. Affected children may also manifest with focal neurological signs or raised intracranial pressure due to obstruction of CSF pathways. Ataxia and sudden onset of severe neurological dysfunction are more common in children with infratentorial lesions. Diagnosis is based on neuroimaging and response to therapy as the CSF findings and culture are usually negative [49, 57].

On CT, tuberculomas are hypo- or isodense with ring or nodular enhancement and moderate perilesional oedema, [50]. CT scans are helpful in identifying calcifications, which are seen in about 10% of tuberculomas [71]. The imaging features of tuberculoma on MRI depend on the pathological state of its centre, which can be noncaseating, solidly caseating, caseating with central liquefaction or calcified [49]. The non-caseating granuloma is hypointense on T1 and hyperintense on T2 and shows no diffusion restriction and homogeneous enhancement [72]. The caseating granuloma is isointense to cortex on T1, has characteristic low signal on T2 and shows peripheral or homogenous enhancement and no diffusion restriction [50]. When the caseating granuloma liquefies, central T2 hyperintensity and diffusion restriction can be seen; this may be difficult to distinguish from a tuberculous or pyogenic abscess on imaging [72]. Magnetic resonance spectroscopy (MRS) can also assist as it demonstrates spectra of characteristic resonances especially in the lipid, lactate and choline spectra [73]. A lipid peak on MRS in the context of a ring-enhancing lesion is very suggestive of tuberculoma as a result of lipids in the cell wall [72] (Fig. 21). A choline peak at 3.2 ppm in addition to a lipid peak at 1.3 ppm is considered characteristic of tuberculoma [73]. The response to treatment can be judged by the degree of contrast enhancement on follow-up imaging. Late changes include calcification and regional atrophy, although many lesions leave no trace following successful treatment [49].

**Fig. 23:**
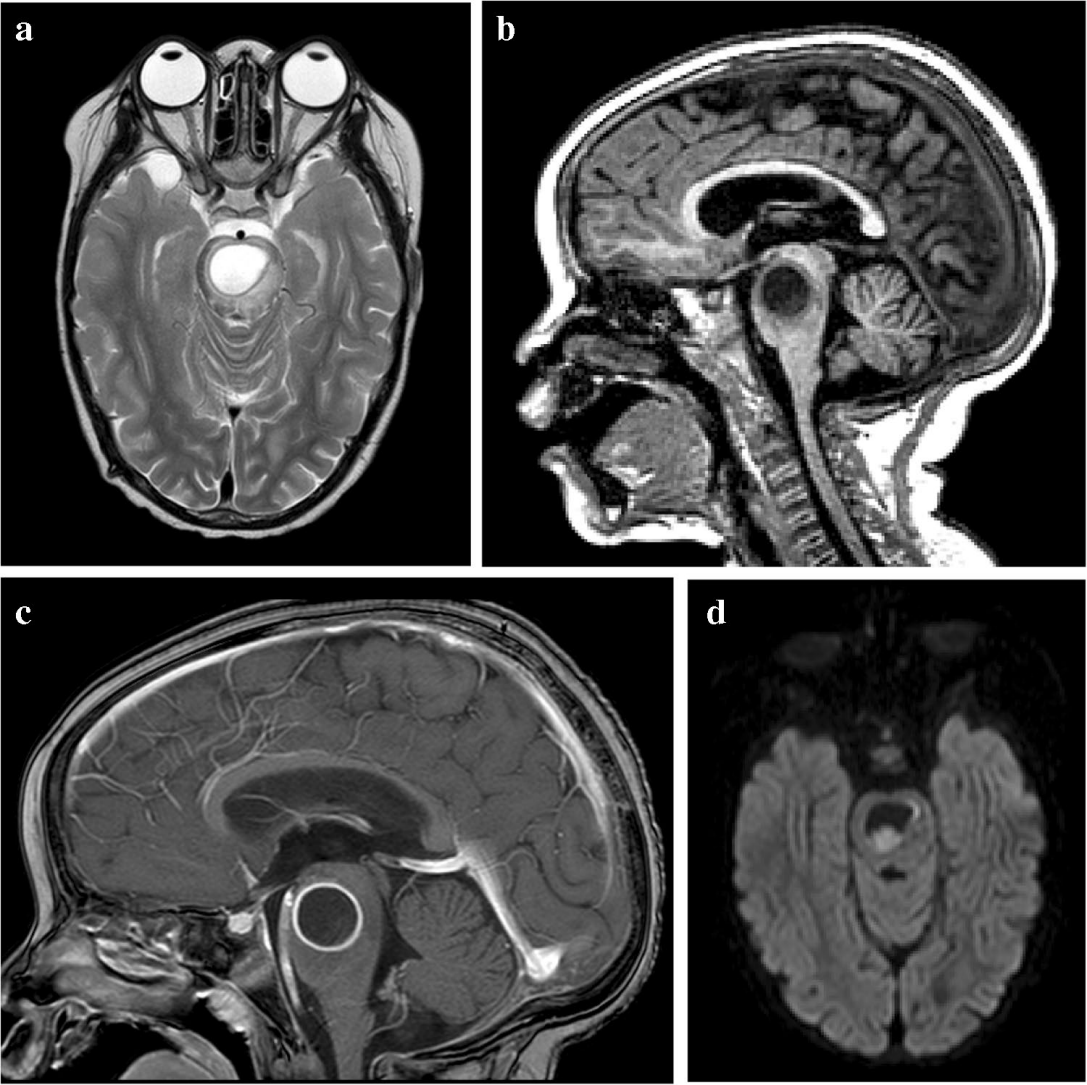
Brain magnetic resonance imaging in a 12-year-old boy **a** Axial T2 demonstrates a hyperintense lesion in the pons with a hypointense rim. **b, c** Sagittal images show intermediate to low signal on T1 (**b**) with peripheral enhancement post-gadolinium (**c**). **d** Axial diffusion weighted imaging shows some restriction in the wall and content. There was corresponding low signal on apparent diffusion coefficient map (not shown). The differential for these imaging findings includes a tuberculous (TB) abscess or liquifying tuberculoma. The patient’s clinical condition deteriorated requiring drainage; surgery confirmed a TB abscess with the content teeming with TB bacilli

Tuberculous abscess formation is a rare complication of TB of the central nervous system [72]. It can develop from parenchymal granulomas or via the spread of tuberculous foci in the meninges to the brain parenchyma in patients with TB meningitis [49]. A TB abscess is an encapsulated collection of pus formed secondary to liquefactive caseation of cerebritis with abundant viable tubercle bacilli. This is in contrast to a tuberculoma, which contains few bacilli and its wall lacks the giant cell granulomatous reaction seen with a tuberculous abscess [49, 72]. The appearance is more akin to pyogenic brain abscess but with a thicker wall. It is essential to differentiate tuberculoma from TB abscess because treatment of tuberculoma is medical, whereas TB abscess has a more accelerated clinical course, does not respond to medical management and usually requires surgical drainage [57]. On CT, a TB abscess is hypodense with perilesional oedema and ring enhancement post-contrast with local mass effect. On MRI, it may be either uni- or multilocular, greater than 30 mm in size and well-defined with perilesional oedema causing mass effect [49]. Contents of the abscess are hypointense on T1 and hyperintense or heterogeneous on T2 with variable intensity on FLAIR, restricted diffusion and with low apparent diffusion coefficient values [72] (Fig. 23). The abscess wall is usually isointense on T1 and hypointense on T2 images. A TB abscess usually has a thicker wall than a pyogenic or fungal abscess [73]. Structural neuroimaging does not allow differentiation of TB and pyogenic abscesses but lipid peaks on MRS are suggestive of mycobacteria [72, 73]. MRS may show a cytosolic amino acid peak in pyogenic abscess and a trehalose peak in addition to amino acid peak in cases of fungal abscess [72]. Generally, a multiloculated lesion, greater than 30 mm with restricted diffusion, and a lipid peak suggest tubercular abscess [72].

### Spinal tuberculosis

**Fig. 24:**
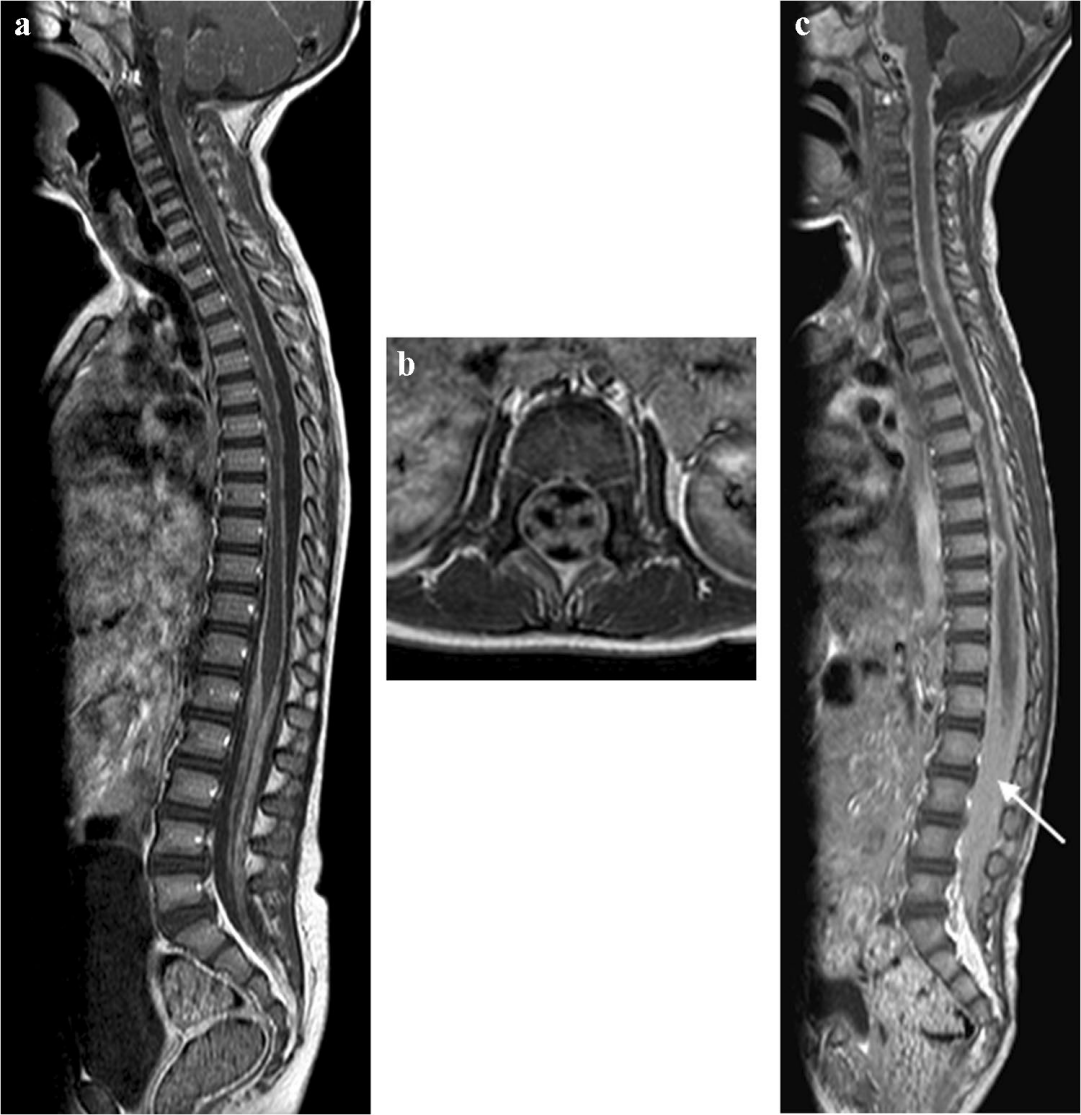
Sagittal (**a**) and axial T1 post-gadolinium (**b**) magnetic resonance imaging (MRI) in a 6-year-old girl with tuberculous (TB) meningitis show enhancement of the arachnoid with enhancement and clumping of the nerve roots. **c** Sagittal T1 post-contrast MRI in a different patient with TB meningitis, a 4-year and-10-month old girl who had repeated failed lumbar punctures, shows the spinal canal to be completely ocupied by enhancing tissue (*arrow*)

Spinal arachnoiditis has been reported on MRI in up to 70% of paediatric TB meningitis patients [65]. Pathologically, it is characterised by a granulomatous exudate that fills the subarachnoid space and extends over several segments. The MRI findings include enhancing subarachnoid nodules, clumping of the cauda equina nerve roots, CSF loculation and abnormal signal and enhancement of the cord [50]. Post-contrast T1 shows mild coating of the cord or near-complete obliteration of the subarachnoid space with nodular or linear thick enhancement at the cauda equina [65] (Fig. 24). The reliability of lumbar puncture opening pressure, the ability to obtain CSF from the lumbar cistern, the interpretation of the protein content in CSF and the success of air encephalography may be affected by spinal disease [65]. The spinal cord can be directly or indirectly affected with cord T2 high signal representing oedema, cord infarct or myelitis that may result in syringohydromyelia [49].

Spinal arachnoiditis is often asymptomatic, possibly because the exudate does not disturb cord or nerve root function [74]. More severe cases can progress to motor and/or sensory deficits with a poor outcome [74].

**Fig. 25:**
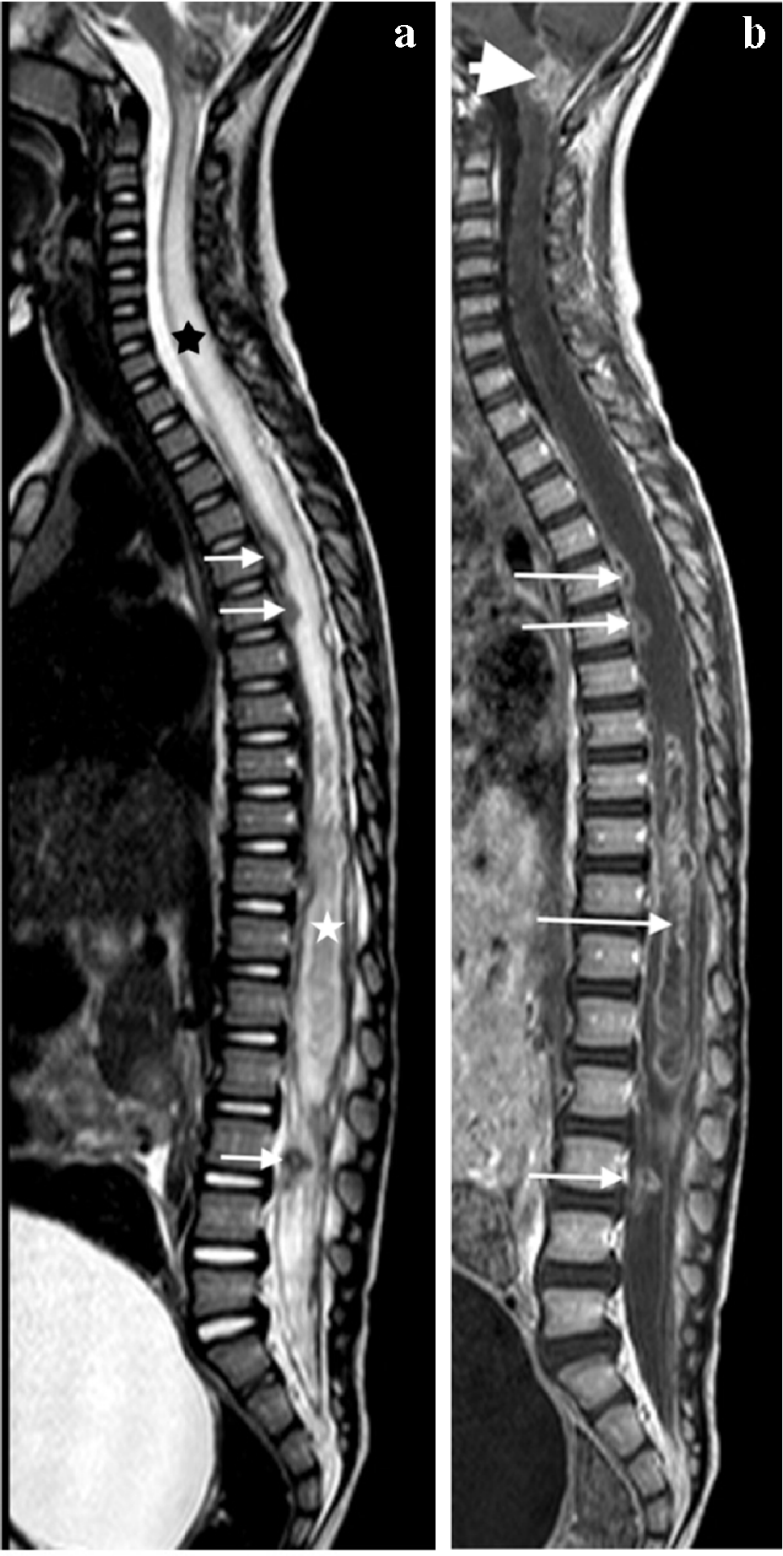
Sagittal magnetic resonance images in a 3-year-old girl with tuberculous meningitis and paraplegia. **a** T2 image demonstrates an irregular hypointense intramedullary lesion from T7 (*black asterisk*) to the conus (*white* *asterisk*); additional lesions are seen of the anterior cord at T6/T7 and within the nerve roots (*arrows*). There is marked cord oedema extending to the cranio-cervical junction. **b** T1post-gadolinium image demonstrates peripheral enhancement of the lesions (*thin* *arrows*) consistent with tuberculomas. Multiple small enhancing tuberculomas are also seen at the cranio-cervical junction (*thick* *arrow*)

**Fig. 26:**
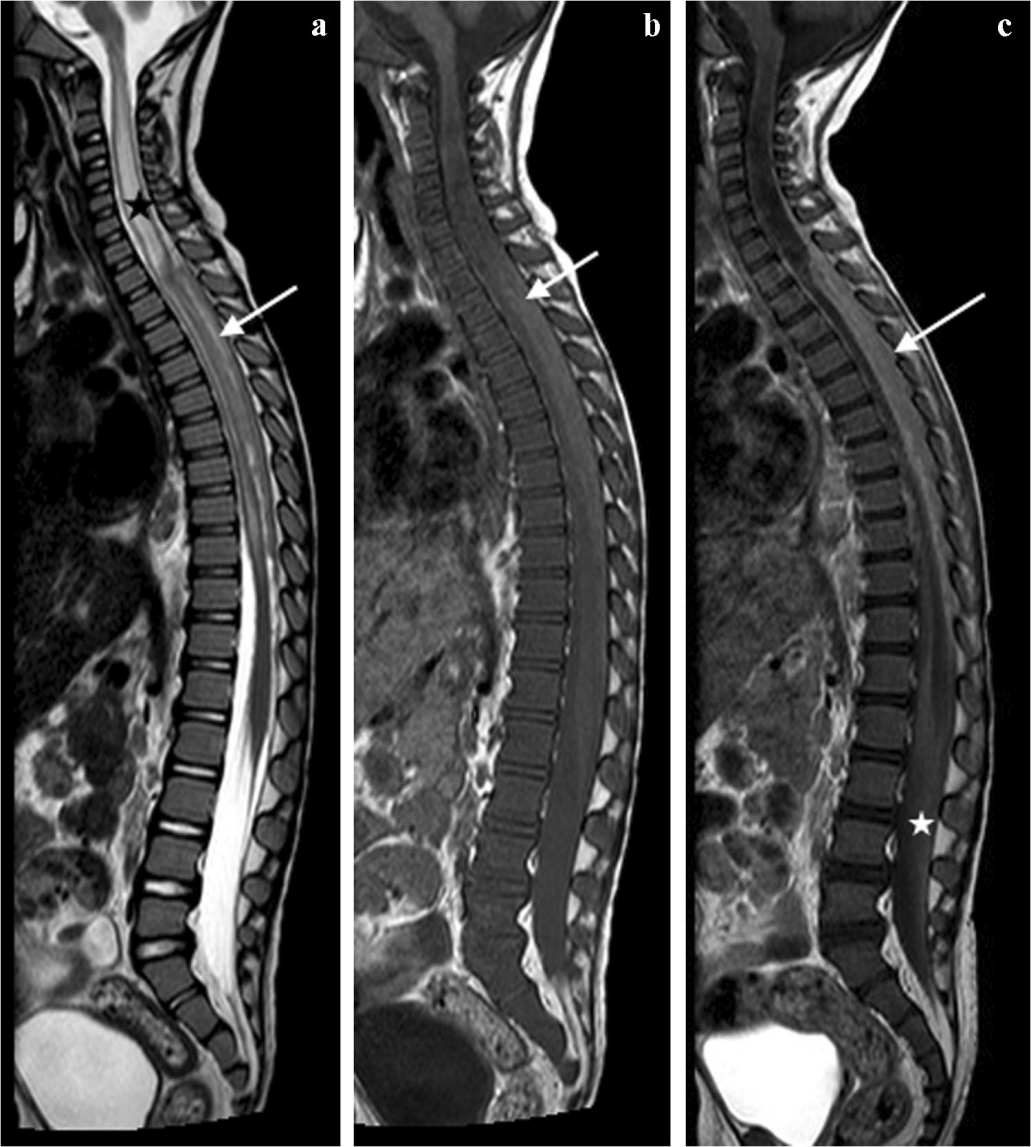
Sagittal magnetic resonance images of the spine in a 5-year-and-3-month-old girl with tuberculous meningitis and progressive lower limb weakness. **a** T2 image shows a hypointense posterior epidural mass extending from T2 to T9 (*arrow*) and compressing the cord anteriorly. There is cord oedema proximal to the mass (*asterisk*). **b**, **c**  T1 pre- (**b**) and post- (**c**) gadolinium show the mass to be homogeneously enhancing (*arrows*). There is also nerve root enhancement (*asterisk in ***c**)

Spinal cord tuberculomas/abscesses are rare [49]. They arise from haematogenous dissemination and have the same imaging characteristics as their intracranial counterparts (Fig. 25). Epidural TB lesions are isointense to the spinal cord on T1 and mixed signal on T2 sequences. Enhancement post-contrast will be peripheral if a true abscess has occurred [49]. These lesions may result in extrinsic compression of the spinal cord (Fig. 26).

### Musculoskeletal tuberculosis

**Fig. 27:**
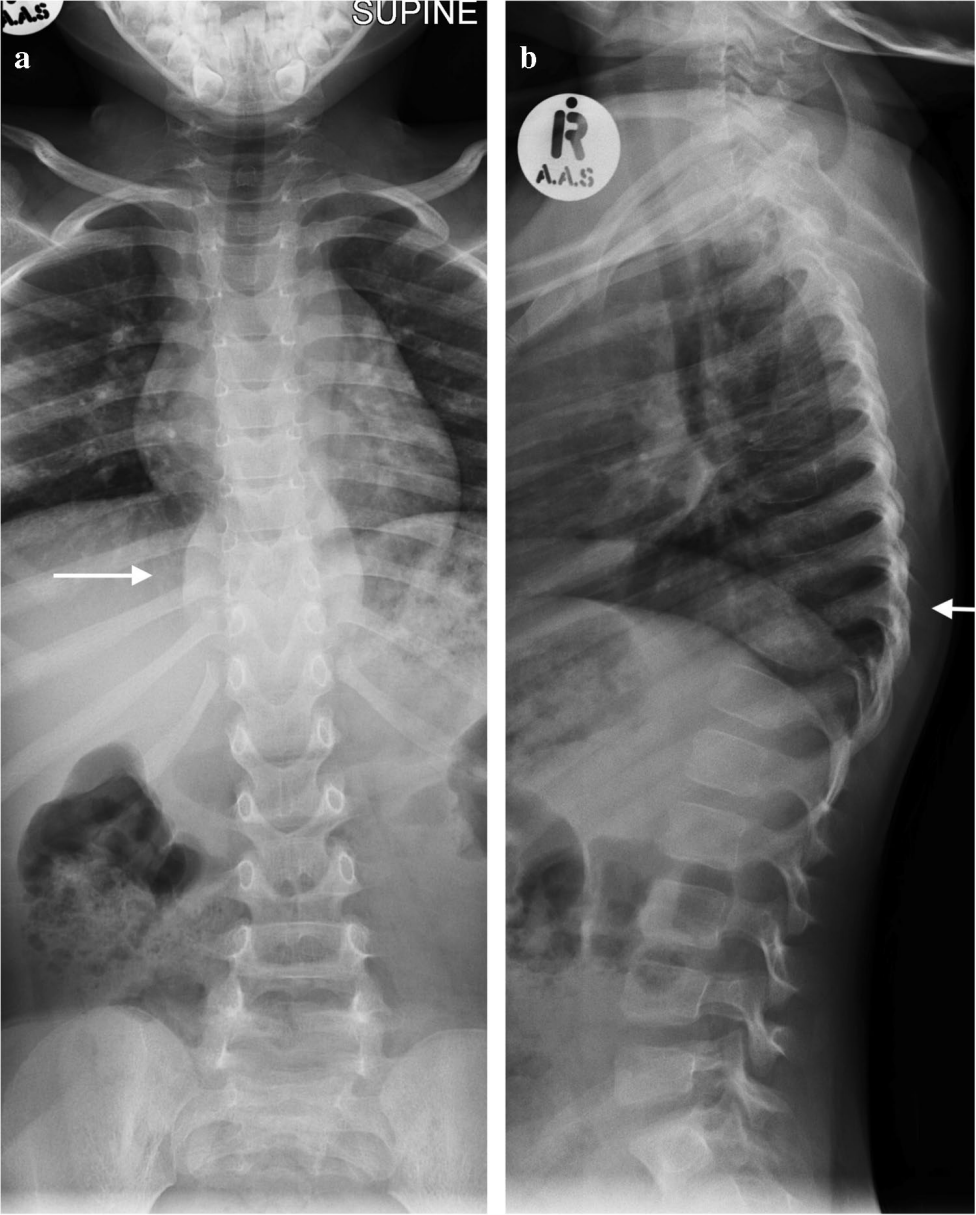
Spine radiographs in a 3-year-old boy who presented with a lump on his back and in whom tuberculosis was confirmed. **a** The anteroposterior radiograph shows a  paravertebral soft tissue mass extending from T8 to T11 (*arrow*). **b** The lateral radiograph shows a thoracic kyphosis due to collapse of the T10 vertebral body (*arrow*)

**Fig. 28:**
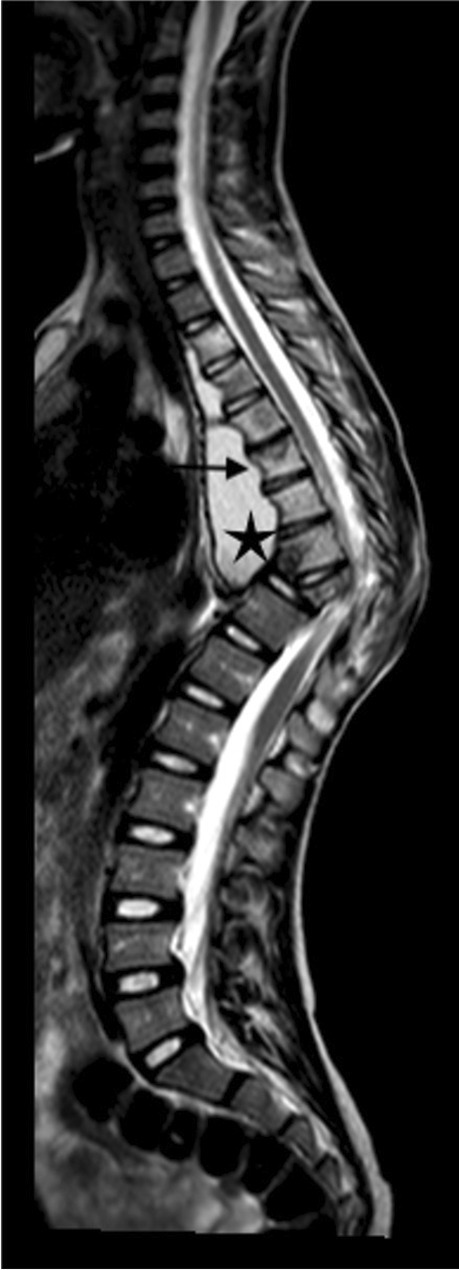
A 6-year-old boy with tuberculous spondylitis. Sagittal T2 magnetic resonance image of the spine shows scalloping of the anterior vertebral bodies (*arrow*) secondary to an anterior subligamentous collection (*asterisk*). There is a thoracic kyphosis due to collapse of the T10 vertebral body

**Fig. 29:**
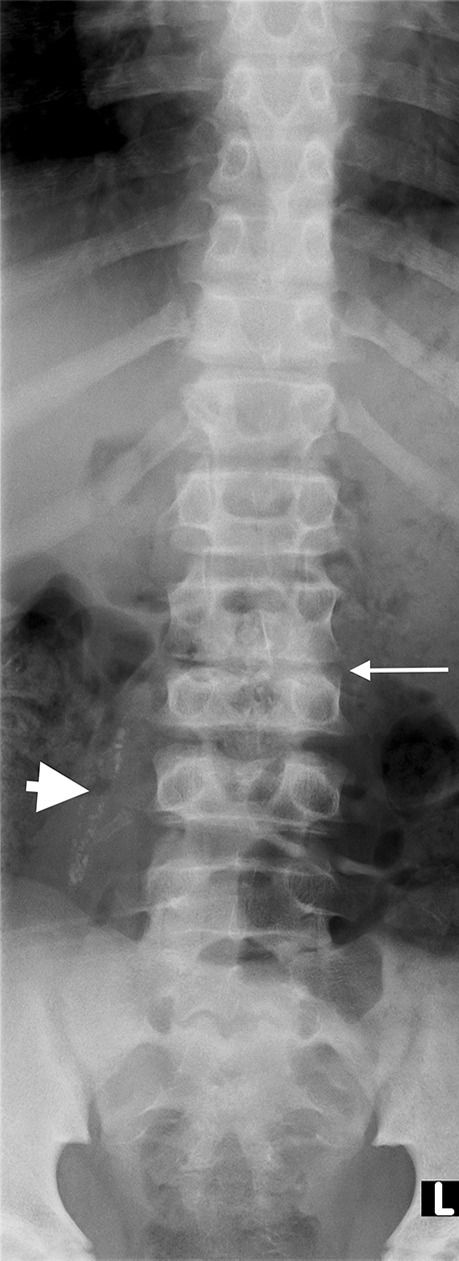
Anteroposterior radiograph of the lumbar spine in a 10-year-old boy with a history of treated tuberculous spondylitis shows disc space narrowing at L2/L3 and loss of height of L3 with sclerosis of its superior endplate (*thin arrow*). There is calcification of the right psoas muscle (*thick arrow*)

Tuberculous spondylitis and vertebral infection result from haematogenous dissemination or latent reactivation. The lower thoracic and upper lumbar levels are most affected [75, 76]. Tuberculosis affects the vertebral bodies early, with disc destruction only seen later in the disease process; infection begins in the anterior vertebral body adjacent to the superior and inferior endplates and spreads to involve the adjoining disc spaces either by extension beneath the anterior or posterior longitudinal ligament or penetration of the subchondral bone plate. Involvement of the disc manifests as disc space loss [75]. Despite being the mainstay of diagnosis, particularly in resource-limited settings, conventional radiographs have disadvantages. At least 50% of the vertebral body needs to be destroyed before changes are evident and this may take up to 6 months from the time of infection [77]. Findings range from a ‘normal’ vertebral body or anterior wedging to complete loss of the vertebral body with resultant kyphosis [78] (Fig. 27). Subligamentous extension and paravertebral collections cause scalloping and erosion of the anterior vertebral bodies and paravertebral and psoas soft tissue masses [79] (Fig. 28). Radiographic progression of bone destruction may continue for up to 14 months post-initiation of anti-tuberculous therapy and should not be considered treatment failure [80]. Sclerosis, periosteal reaction and soft tissue calcification occur late or following treatment [81, 82] (Fig. 29).

**Fig. 30:**
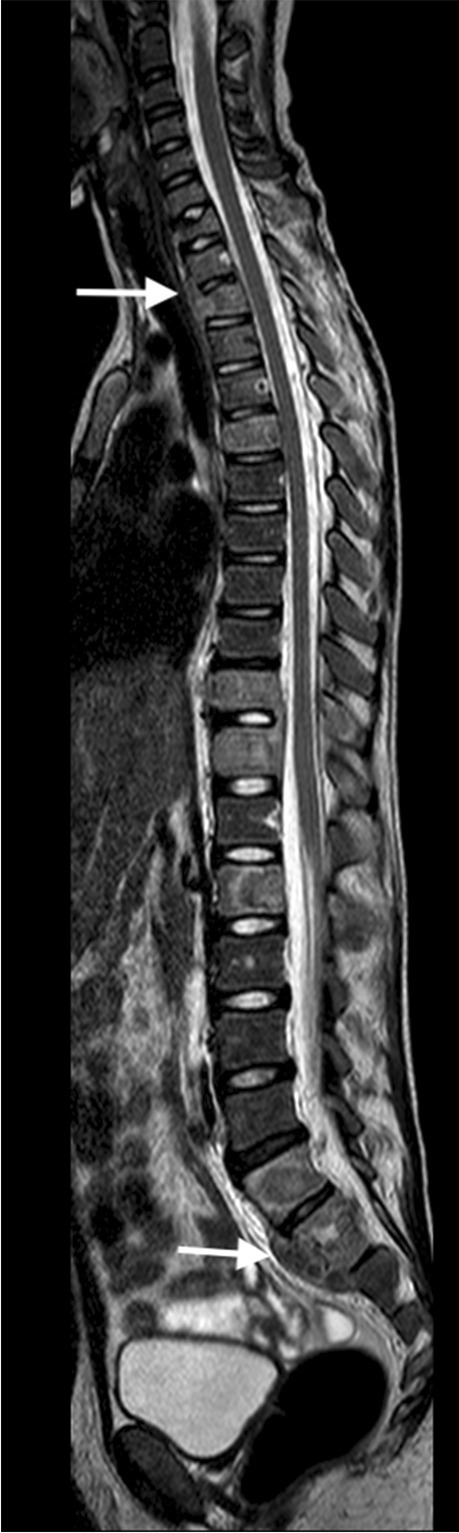
A sagittal T2 magnetic resonance image of the spine in a 7-year-old girl with tuberculous spondylitis. There is multilevel non-contiguous disease as evidenced by the increased signal in the vertebral bodies; note the anterior subligamentous spread at T1/T2 and S1/S2 (*arrows*). There is preservation of the disc height at all levels and disc signal at almost all levels

MRI is the imaging modality of choice in TB spondylitis as it avoids ionising radiation and demonstrates the spinal cord, intervertebral discs and any soft tissue masses. The entire spine should be imaged to identify multiple non-contiguous areas of disease [83, 84] (Fig. 30). Involved vertebral bodies are hypointense on T1 and hyperintense on T2 with contrast enhancement either restricted to the endplates or more diffuse [75].

Abnormal signal often extends into the pedicles, although the cortex is usually maintained [78].

**Fig. 31:**
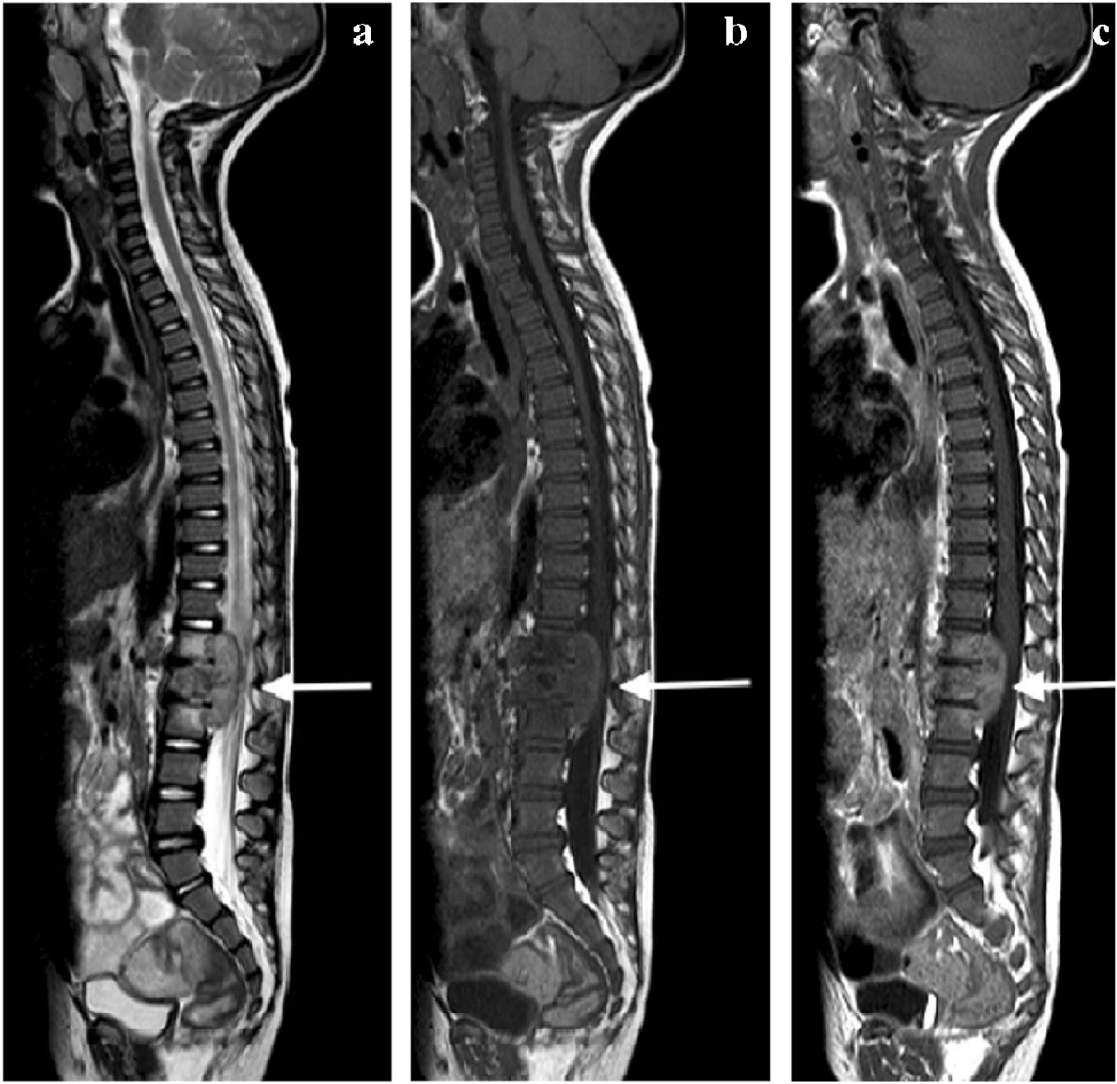
A 2-year-old boy with back pain. **a**–**c** Sagittal T2 (**a**), T1 (**b**) and T1 post-gadolinium (**c**) magnetic resonance images of the spine show the involved T12, L1 and L2 vertebral bodies with a soft tissue mass that enhances homogeneously and protrudes posteriorly into the spinal canal compressing the cauda equina (*arrows*). Note the enhancing vertebral bodies and the T2 hypointense non-enhancing discs, also the involvement of the dens of C2 that was not suspected clinically. Tuberculosis was confirmed

**Fig. 32:**
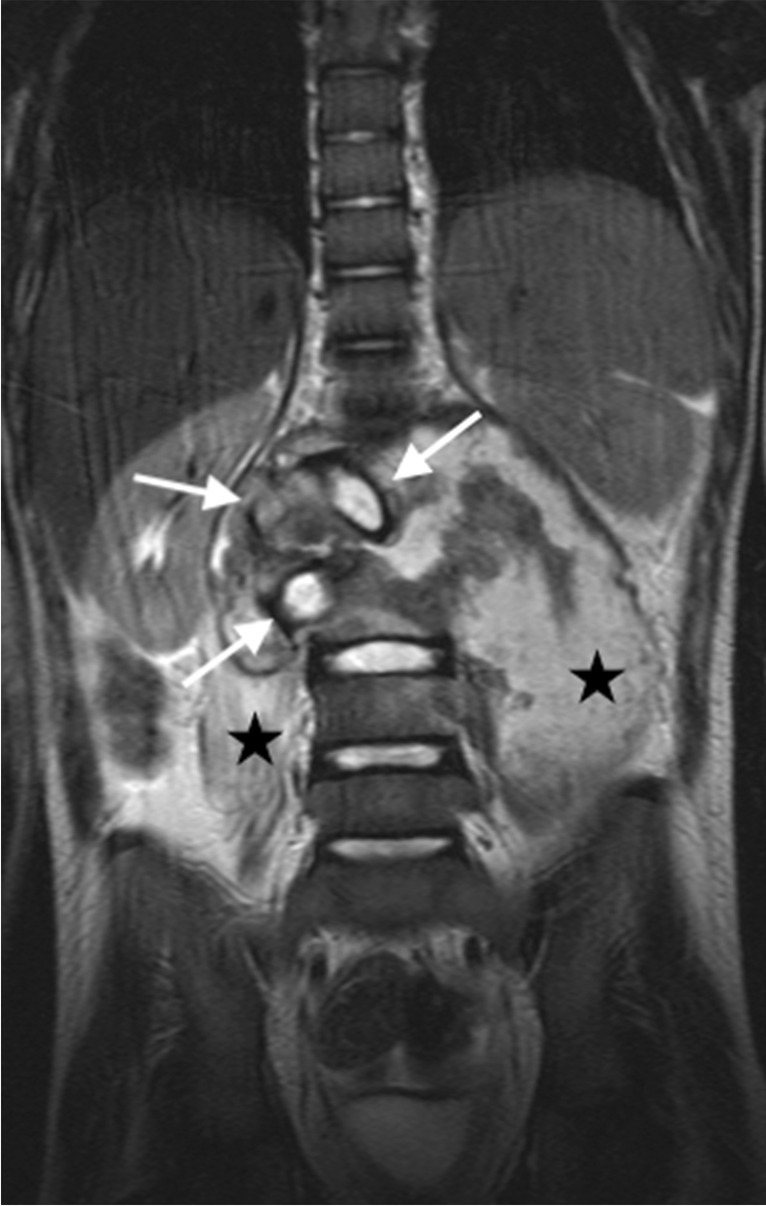
A coronal T2 magnetic resonance image in an 8-year-old girl with tuberculous spondylitis shows expanded discs with normal signal (*arrows*) subsumed within a large paraspinal collection that extends into both psoas muscles (*asterisks*)

The spinous processes are invariably spared and therefore may be helpful in defining the number of bodies involved in multilevel contiguous collapse [85]. The appearance of the disc is variable and independent of the stage and severity of the disease. It may retain T2 high signal or become hypointense and lose volume [78] (Figs 30, 31 and 32).

**Fig. 33:**
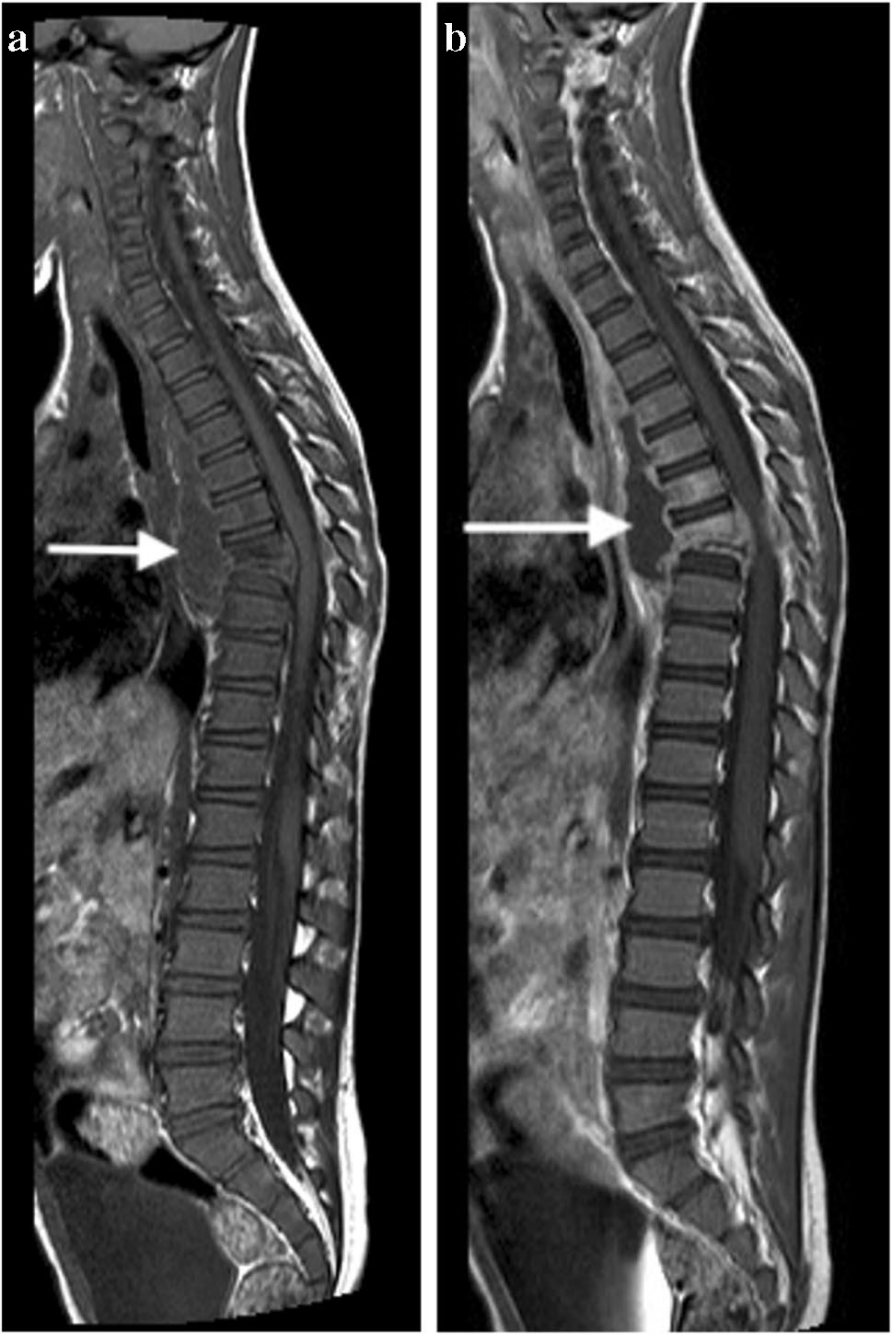
Sagittal T1 pre- (**a**) and post-gadolinium (**b**) magnetic resonance images in a 5-year-old girl who presented with kyphosis. There is a hypointense peripherally enhancing anterior subligamentous collection (*arrows*) causing scalloping of the anterior vertebral bodies. There is kyphosis centred around the destroyed T7 and T8 vertebral bodies (identified by way of their intact posterior elements). Tuberculosis was confirmed

**Fig. 34:**
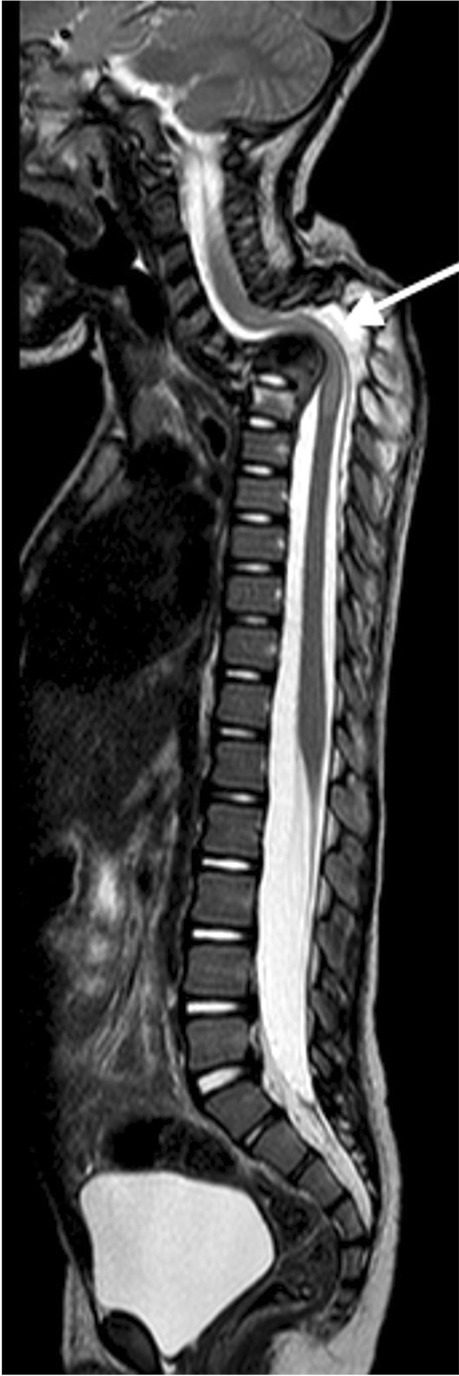
Sagittal T2 magnetic resonance image in a 4-year-old boy presenting with kyphosis and tetraparesis. There is cervicothoracic kyphosis and the cord is draped over the apex of the gibbus with localised cord signal abnormality (*arrow*). The patient made a full recovery post-surgical and anti-tuberculous therapy

The associated epidural mass is low–intermediate signal on T1 and intermediate–high signal on T2-weighted images. Paraspinal collections return the signal of proteinaceous fluid on all pulse sequences. These occur more commonly than in pyogenic infection and may be bilateral, disproportionately larger than the area of bone involvement and distant from the spondylitic source [78, 82]. Enhancement of the epidural mass and paraspinal collection may be diffuse and homogeneous or peripheral; when solid, this suggests caseation without necrosis [75, 82] (Fig. 33). The slow progression of TB spondylitis allows the cord to accommodate the reduced canal volume; it has been reported that 75% volume loss may occur before symptoms emerge [13, 14]. Increased cord signal is associated with a worse prognosis; however, even with complete paralysis at presentation, a high percentage of patients are ambulatory post-treatment [86, 87] (Fig. 34). CT is superior to radiography in demonstrating bone destruction, calcification and soft tissue changes but bone oedema and cord detail cannot be assessed. Since it is more widely available than MRI (especially in resource-limited settings where TB is endemic), CT may be useful in the setting of acute myelopathy for surgical planning; however, the radiation dose is of concern in children [78].

Tuberculous arthritis is the second most common form of skeletal tuberculosis.

It occurs either due to direct spread from adjacent metaphyseal lesions into the joint space (which is uncharacteristic of pyogenic arthritis) or less commonly by haematogenous spread [88, 89]. Single-joint involvement is more common, with the large joints such as hip and knee being most frequently involved [89, 90]. Common clinical manifestations include joint pain, stiffness and functional loss ocurring over months [91].

**Fig. 35:**
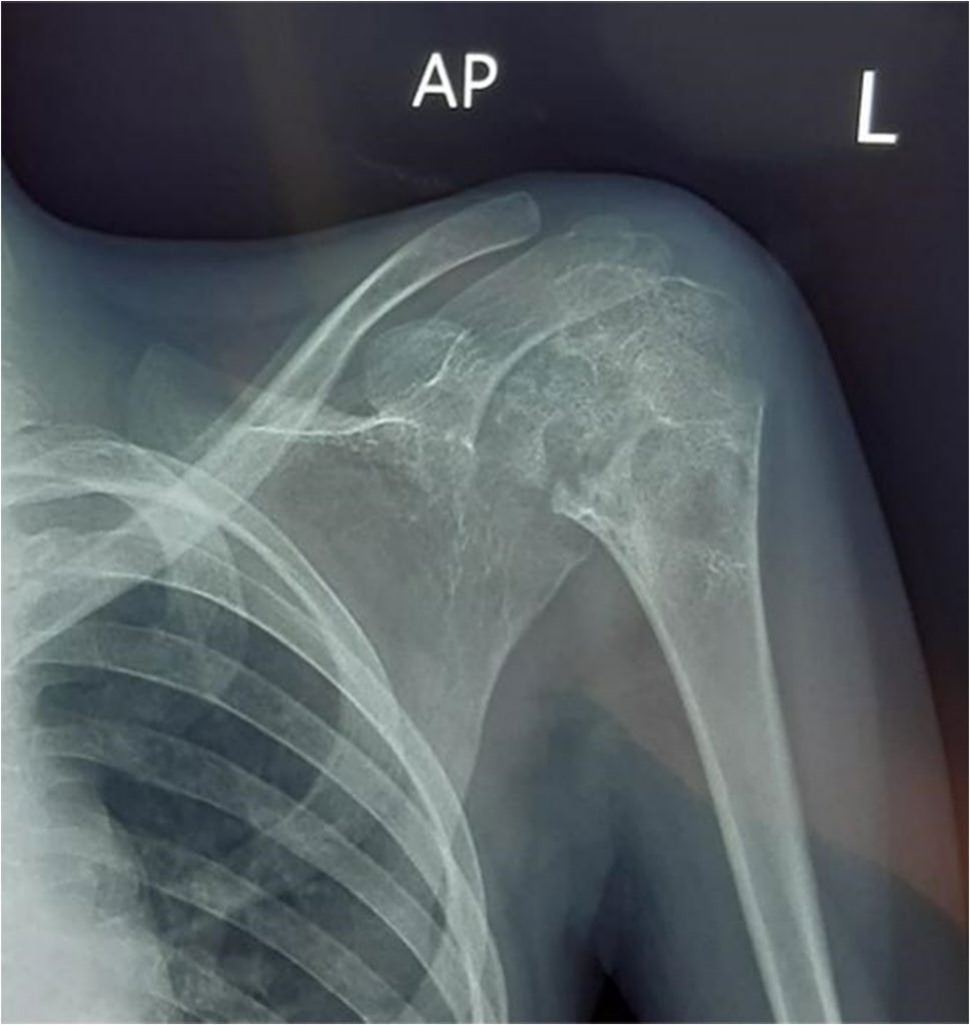
Anteroposterior shoulder radiograph in a 4-year-old girl presenting with left shoulder pain demonstrates the Phemister triad: juxta-articular osteoporosis, peripheral osseous erosions and narrowing of joint space, typical of tuberculous arthritis. Tuberculosis was confirmed on bone biopsy

 Radiography is the mainstay of initial investigation, although abnormalities may not be readily appreciated until the late stage of disease. Tuberculous arthritis resulting from direct transphyseal spread initially manifests as periarticular osteopaenia with focal bony destruction [88–90]. Joint swelling and effusion due to the presence of synovial hypertrophy may be the only radiographic findings in the early stages of TB arthritis from haematogenous spread [88–90]. As disease progresses, there may be adjacent bony changes including periarticular osteopenia, bone erosions and cortical irregularity with eventual loss of joint space and formation of tiny intra-articular loose bodies known as ‘rice bodies’ [88–90]. The Phemister triad, which comprises of juxta-articular osteoporosis and peripheral osseous erosions with gradual narrowing of joint space, is typical of TB arthritis (Fig. 35), but is also seen in rheumatoid arthritis [90, 91]. Layered periosteal reaction, which is uncommonly seen in adult TB arthritis, may be seen in paediatric patients [89, 91]. Fibrous ankylosis, joint destruction and pathological subluxation/dislocation are the characteristics of end-stage TB arthritis [88–90]. Bone ankylosis, as seen in pyogenic arthritis, is uncommon in TB arthritis [88–90]. Epiphyseal hyperaemia in paediatric patients may result in advanced maturity of the epiphyseal plate with widening of the intercondylar notch and limb shortening [89]. Ultrasound may aid in the initial evaluation of TB arthritis by demonstrating joint effusion and may be used to guide joint aspiration for microbiological diagnosis [88–90]. CT, which is superior to conventional radiography, can detect early TB arthritis, confirm the extent of bone destruction and identify sequestration [91].

MRI is the imaging modality of choice in detecting early TB arthritis. Synovial proliferation of TB arthritis is characteristically T2 hypo- to isointense, allowing its differentiation from other arthropathies [88–90]. This is due to the mixture of inflammatory tissue, necrosis, prior blood degradation products and fibrosis [88, 90]. Cartilaginous destruction, subchondral erosions and bone marrow oedema with soft tissue abnormalities, e.g., myositis, soft tissue abscess or fistula formation, are also MRI features of TB arthritis [88–90].

Differential diagnosis of TB arthritis includes other pyogenic arthritides and rheumatological conditions such as rheumatoid arthritis or juvenile idiopathic arthritis [89, 90].

A study by Sung et al. comparing TB and pyogenic arthritis concluded that subchondral marrow signal abnormality and bony erosion were more frequently seen in TB arthritis [92]. Cold abscesses from TB infection show a thin enhancing rim, whereas pyogenic abscesses show a thick irregular rim [92]. Rheumatoid arthritis tends to cause smaller erosions with non-uniform synovial thickening [93]. Extraarticular cystic masses are commonly seen in TB arthritis. Definitive diagnosis requires joint aspiration or biopsy of the synovium [89, 90].

Poncet’s arthritis, a rare form of aseptic reactive arthritis, is commonly underrecognised [88, 91, 94]. It usually occurs in patients with extrapulmonary TB, especially those with erythema nodosum [94]. Patients usually present with acute polyarthritis [94, 95] in contrast to monoarticular involvement in TB arthritis. Symptoms typically subside within a few weeks of medical treatment, with no residual joint destruction [94, 95].

Tuberculous osteomyelitis is much less common, accounting for 11% of musculoskeletal manifestations of TB [96]. Through the haematogenous route of dissemination, TB osteomyelitis commonly occurs multifocally as part of primary infection in infants and younger children, while in older children, a solitary focus of infection as part of reactivated TB is usually seen [90, 97]. Metaphyses of the long bones, especially the lower limbs, are commonly involved in paediatric patients [90, 96]. Patients usually present with long-standing bone pain and swelling [90].

**Fig. 36:**
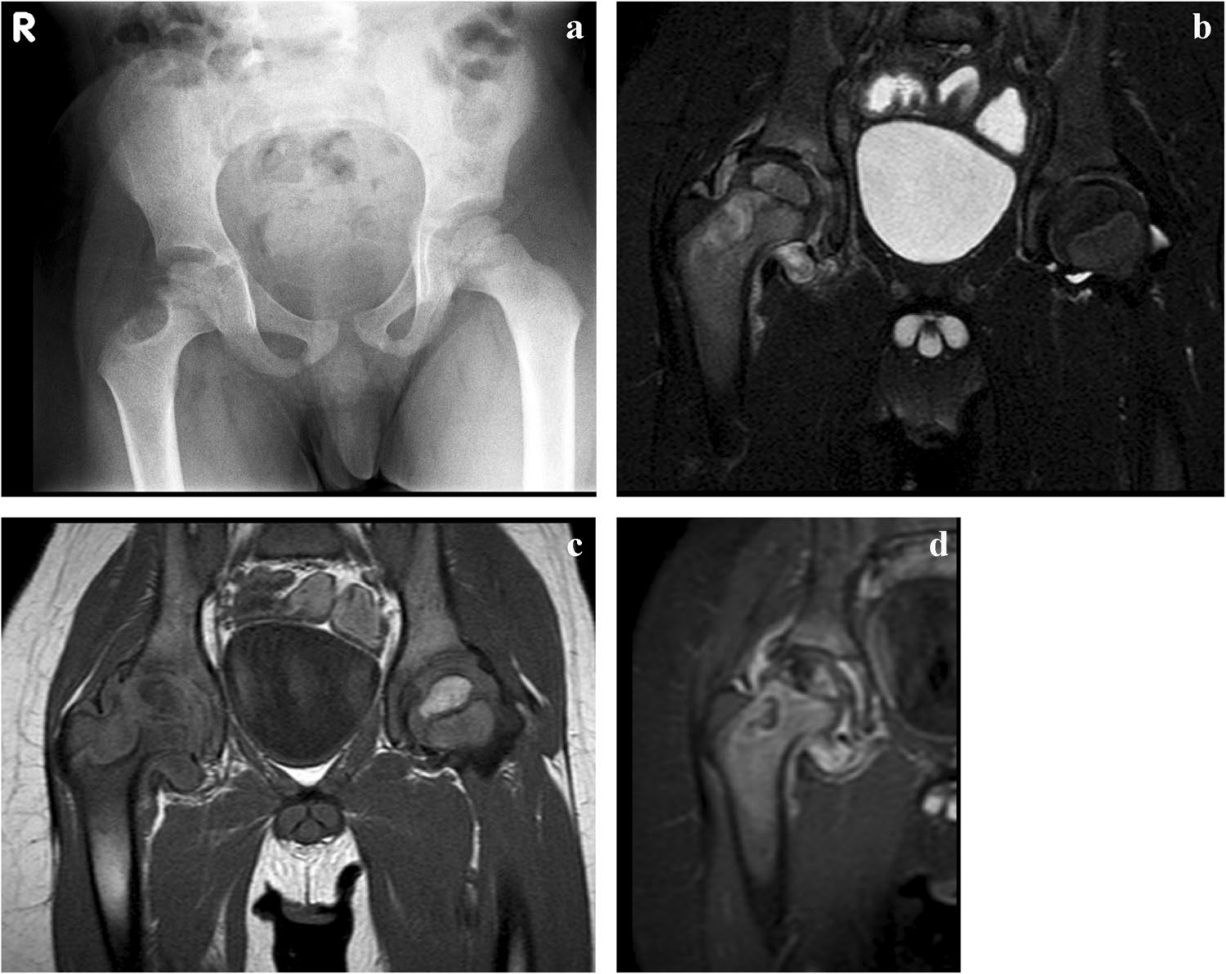
Cystic tuberculous (TB) osteomyelitis in a 7-year-old boy who had been limping since a minor fall 3 months previously. **a** An anteroposterior pelvic radiograph shows a well-defined eccentrically located cystic lesion of the lateral right femoral neck with cortical irregularity of the adjacent metaphysis. The femoral head is osteopaenic. **b-d** Coronal magnetic resonance images. T2 with fat suppression (**b**) and T1 (**c**) confirm involvement of the metaphysis and epiphysis and that the process crosses the joint to involve the superolateral acetabulum with a small associated effusion. There is heterogeneous enhancement of the bone and diffuse synovial enhancement post-contrast (**d**). Bone biopsy–confirmed tuberculosis

Conventional radiography is the mainstay of initial investigation. Four radiographic appearances of TB osteomyelitis have been described, including cystic form, infiltrative form, focal erosions and spina ventosa [96]. The cystic form of TB osteomyelitis manifests as an eccentric expanding radiolucent lesion, typically in the metaphysis of the affected bone [96]. Bony sequestrum and periosteal reaction, though less frequently seen when compared with pyogenic osteomyelitis, may be seen in some patients [96] (Fig. 36). Infiltrative TB osteomyelitis manifests as extensive bony destruction with permeative margins, mimicking that of chronic osteomyelitis and aggressive tumours [96]. Pathological fracture may occur as an associated complication. The focal erosive form of TB osteomyelitis presents as local bony osteolysis or cortical erosions with minimal marginal sclerosis [96].

CT can be used as an adjunct to allow more accurate assessment of the extent of bony destruction, especially in areas of difficult visualisation on radiographs. MRI is the imaging modality of choice for early detection of TB osteomyelitis. Loss of fat marrow signal on T1 images and T2 hyperintensity with contrast enhancement are typical bone marrow signal changes [88, 90]. Soft tissue involvement, such as associated abscesses, fistula and adjacent sinus tracts are also well visualised on contrast-enhanced sequences [88, 90]. Tissue biopsy for histological diagnosis is often required for confirmation of the diagnosis due to the non-pathognomonic radiological appearances [96].

**Fig. 37:**
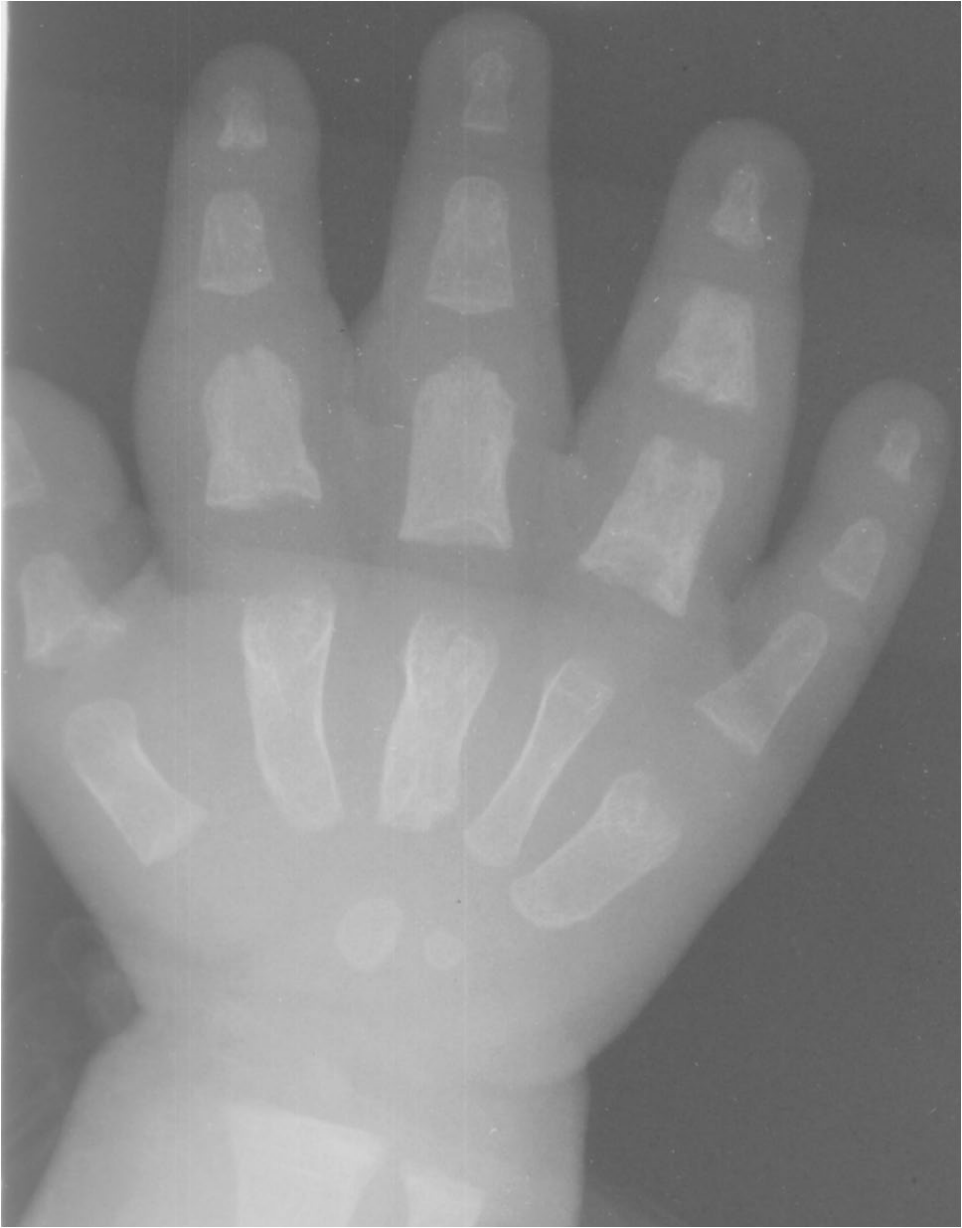
Dorsopalmar radiograph of the right hand in a 9-month-old girl, presenting with a painless swelling of the fingers for 2 months. There is soft tissue swelling of the second through fourth digits with fusiform expansion of the second, third and fifth metacarpals and fusiform swelling and erosion of the first, second, third and fourth proximal and fourth middle phalanges, typical of tuberculous dactylitis (spina ventosa)

Tuberculous dactylitis, also known as spina ventosa, is one of the rare musculoskeletal manifestations of TB. It typically occurs before ossification of the epiphyses in children younger than 5 years of age [90, 98]. Spina ventosa typically affects the short tubular bones, especially of the upper limbs, particularly involving the proximal phalanx of index and middle fingers [90, 99]. *Mycobacterium tuberculosis* rapidly destroys the marrow space replacing and expanding it with granulation tissue [98]. This results in fusiform expansion of the affected bone with internal cyst-like cavities, hence named spina ventosa [88, 89] (Fig. 37). Associated soft tissue swelling, bony sequestration and periosteal reaction have been uncommonly reported [98].

Tuberculous tenosynovitis is an unusual manifestation of extrapulmonary TB, predominantly affecting the flexor tendon sheaths of the hands [88–90]. Ultrasound is the initial imaging modality of choice for diagnosing and evaluating the extent of disease. Diffuse thickening of the tendons and synovium with minimal synovial sheath effusion is usually seen [89, 90], in contrast to the predominant effusions seen in suppurative tenosynovitis [88, 89].

**Fig. 38:**
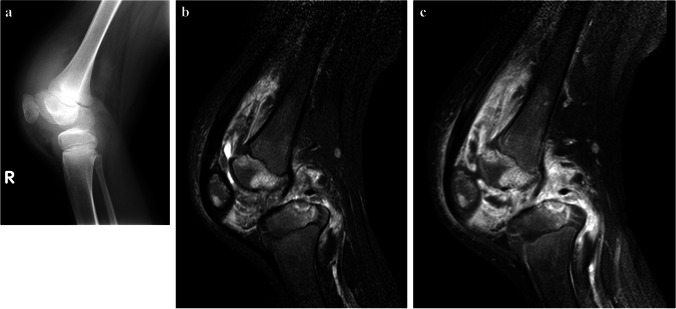
Images of the right knee in a 12-year-old boy with tuberculous tenosynovitis and osteomyelitis. There was a history of a swollen knee for 6 months, no history of trauma and although he was not septic, he was noted to be severely malnourished. **a** A lateral knee radiograph shows a large joint effusion, suspected synovial thickening and osteopaenia of the patella. No periosteal reaction or focal bone lesion is demonstrated. He proceeded to magnetic resonance imaging (MRI) following a failed attempt at joint aspiration. **b **A sagittal short tau inversion recovery MRI shows thickened low signal synovium lining the suprapatellar and popliteal fossa with a small amount of fluid in the suprapatellar space. There is also patchy oedema of the patella and femoral epiphysis with cartilage thinning and a focal erosive lesion of the tibial epiphysis. **c** A sagittal fat saturated T1 post-contrast MRI shows diffuse synovial and patchy multifocal bone enhancement. Synovial biopsy confirmed tuberculosis

MRI helps to precisely delineate the extent of disease. Tuberculous tenosynovitis can occur with osteomyelitis (Fig. 38).

Three stages of TB tenosynovitis have been described by Jaovisidha et al. The hygromatous stage is the earliest stage of involvement in which there is serous exudate within the tendon sheath with no significant or early sheath thickening [100]. Thickening of the tendon sheath with presence of T2 hypointense rice bodies is characteristic of the second stage of disease—the serofibrinous stage [100]. Spontaneous rupture of tendon may occur at this stage with most tendon fibres being replaced by fibrous tissue. The last stage of disease—the fungoid stage—is the end result where there is a soft tissue mass replacing the affected tendon [100].

Tuberculous bursitis is a rare manifestation of extrapulmonary TB, accounting for less than 1% of skeletal manifestations of TB [100]. It commonly affects joints susceptible to trauma [100] such as the trochanteric bursa. Conventional radiographs may demonstrate soft tissue swelling with flecks of calcification [100]. Local osteopaenia may also be seen in chronic cases together with focal bony destruction due to pressure effects [88, 89]. MRI is the main investigation of choice, characteristically showing diffuse bursal distension or the presence of small abscesses [88, 89]. T2 hypointense material within the distended bursa may represent fibrotic material and caseous necrosis [88, 89].

Tuberculous myositis is extremely rare due to the inherent resistance to mycobacterial infection of muscle fibres [88]. It usually occurs due to direct spread from adjacent arthritis or osteomyelitis and is commonly seen in immunosuppressed patients. Chest wall involvement is most common, with direct spread from adjacent pleuritis and lymph node TB [88, 89]. MRI is the modality of choice, showing T1 hypointensity and T2 hyperintensity in the affected muscles. Abscesses are commonly seen and have a T1 hyperintense and T2 hypointense contrast-enhancing rim [88]. Absence of adjacent cellulitis and deep vein thrombosis allows differentiation from other forms of pyogenic myositis [101].

### Abdominal tuberculosis

Despite modern technologies and developments, diagnosis of abdominal TB still represents a significant challenge. Imaging relies on all radiology modalities, including radiography, fluoroscopy, US, CT and MRI, and depends on the presenting symptoms and availability of the imaging equipment, which is often limited, especially in low- and middle-income countries. Abdominal radiography is often non-specific, but may show an abnormal gas pattern, bowel wall thickening and dilatation or separation of the small bowel loops due to an infiltrative process affecting the mesentery and omentum. In case of involvement of solid abdominal organs, organomegaly may be detected. Chest radiography should be performed, as approximately 15–25% of cases with abdominal TB have concomitant pulmonary infection [102].

Abdominal TB presents with a triad of pain, fever and weight loss, occurring in 54% of patients [103]. Although similar symptoms are seen in other abdominal and gastrointestinal infections, bowel inflammatory disease and abdominal malignancies, presence of the triad should always raise a high suspicion of TB, especially in children living in endemic areas [104] and low- and middle-income countries [105]. Abdominal TB may occur in various forms, including TB lymphadenopathy and peritoneal, gastrointestinal and visceral TB. These are separate entities, but there is usually a combination of various pathologies in the individual infected patient. Multiple intra-abdominal sites are involved in more than 50% of patients with a combination of lymphadenopathy and gastrointestinal involvement being the most common [103].

In children with single-site involvement, lymphadenopathy is most common, followed by pathology in the ileocecal region. The type of abdominal infection is closely related to the entry point of the tubercle bacilli into the abdominal cavity. Bacteria ingested with infected milk or sputum affect the mucosal layer of the gastrointestinal tract, with formation of epithelioid tubercles in the lymphoid tissue of the submucosa. Progression of infection causes necrosis of the tubercles and ulceration of the overlying mucosa and bowel wall, with further spread of infection into adjacent mesentery, omentum and lymph nodes. The haematogenous route disseminates bacteria from other centres of infection in the body through the blood stream, to solid abdominal organs, such as the  liver, spleen or kidneys, lymph nodes and peritoneum. Similarly, the lymphatic system offers an easy route for TB bacilli to enter the abdominal cavity from other infected lymph nodes. Other pathways include direct spread from infected adjacent organs, such as the ovaries or fallopian tubes, spine (in the presence of discitis) or from a collection within the psoas muscle [103–105].

**Fig. 39:**
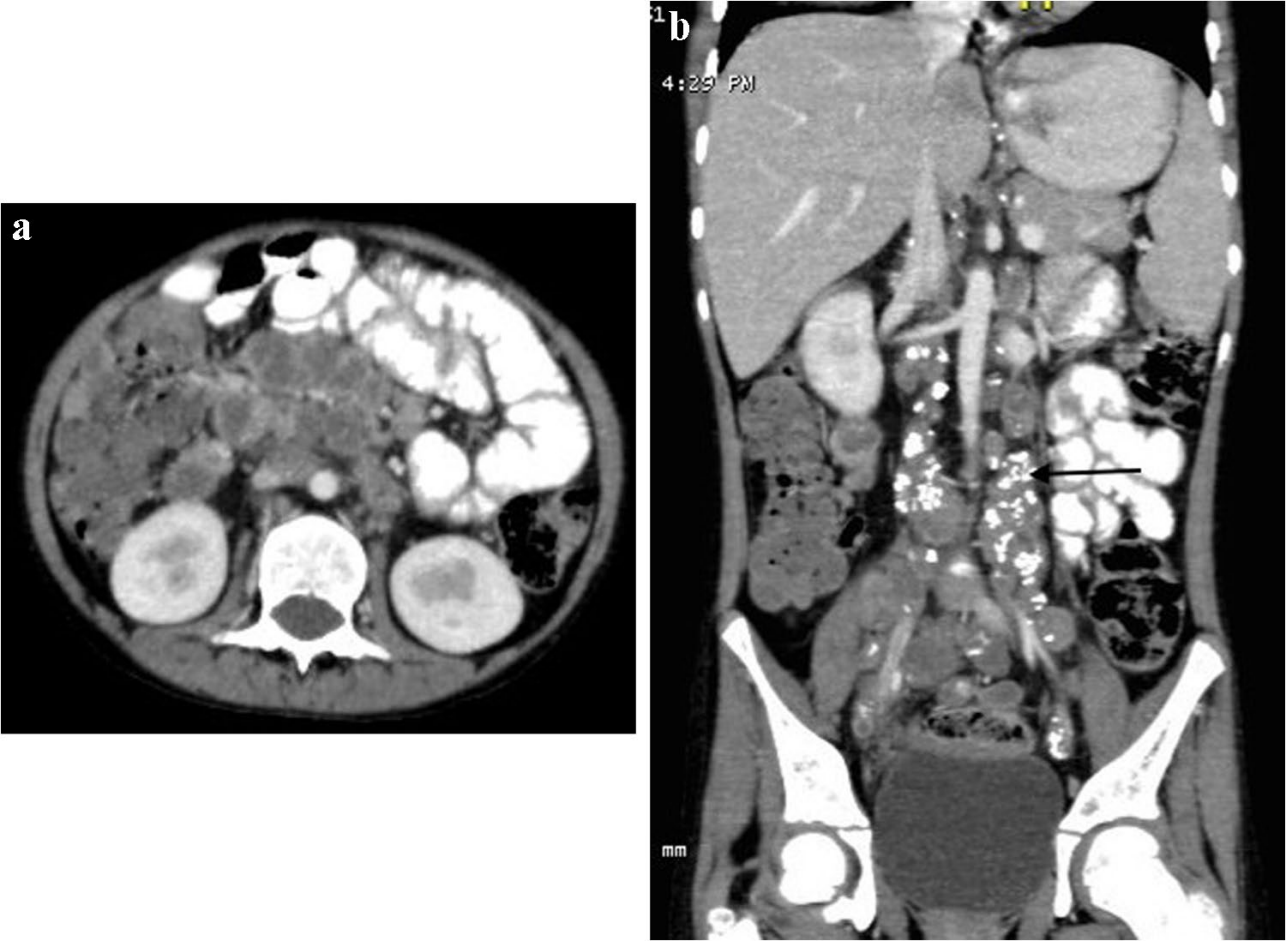
A 9-year-old boy with ileocecal tuberculosis. Axial (**a**) and coronal (**b**) contrast-enhanced computed tomography images show circumferential mural thickening involving the caecum and terminal ileum. Enlarged calcified lymph nodes are also seen on the coronal image (*arrow*)

Lymphadenopathy is the most common manifestation of abdominal TB and is often caused by ingestion of infected food. It mainly affects the lymph nodes in the mesentery and omentum of the epigastrium, at the porta hepatis, along coeliac axis and around the pancreas. There are various patterns of TB lymphadenopathy, with multiple mildly enlarged lymph nodes located in clusters being most common. Central necrosis and peripheral enhancement complete the features of infection [106] (Fig. 39).

**Fig. 40:**
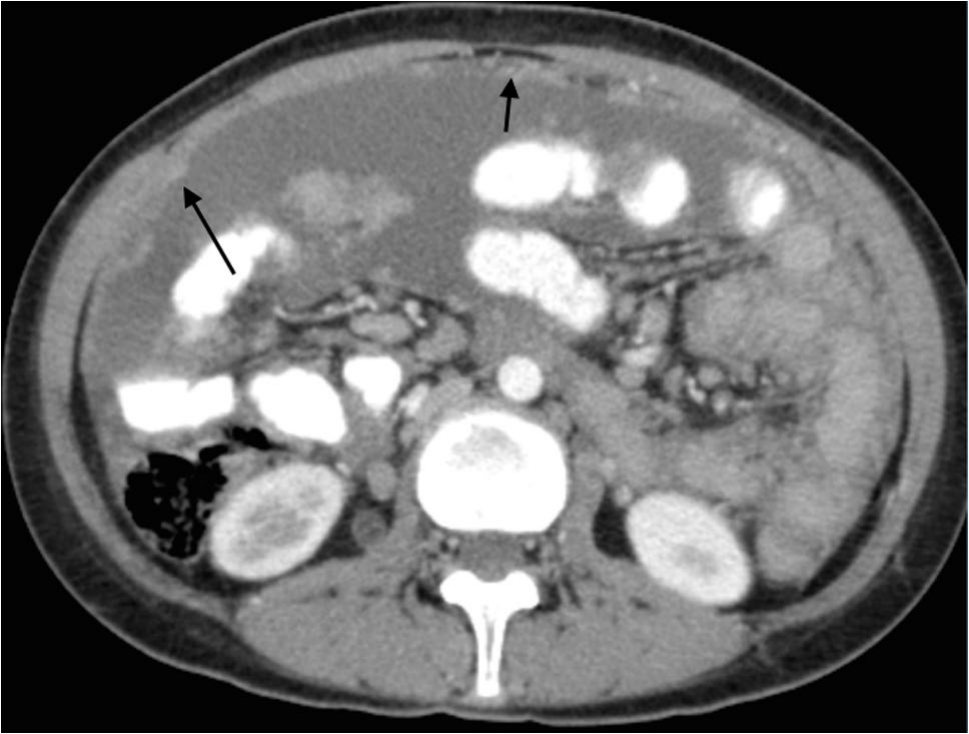
An axial computed tomography image of the mid-abdomen following intravenous and oral contrast administration in an 8-year-old boy shows high attenuating free fluid and fine nodularities in the peritoneum (*arrows*), in keeping with peritoneal tuberculosis of the wet ascitic type

**Fig. 41:**
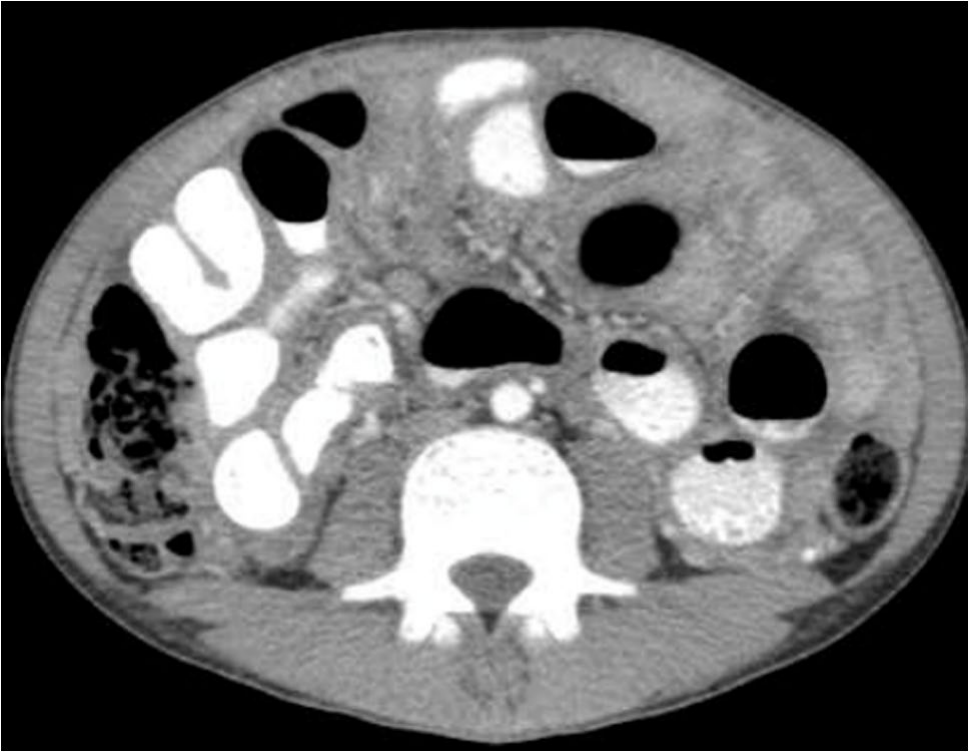
An axial computed tomography image of the mid-abdomen following intravenous and oral contrast in a 3-year-and-5-month-old boy shows diffuse thick hazy stranding of the peritoneum separating the intestinal loops, in keeping with peritoneal tuberculosis of the dry plastic type

Peritoneal involvement is usually divided into three types, depending on the most dominant pathological process. However, all processes are often present concomitantly, with a dynamic and changing clinical and pathological picture. The wet ascitic type is characterised by a large volume of free or loculated fluid of high density (due to increased protein levels) and prominent peritoneal enhancement (Fig. 40). The dry plastic type is characterised by a fibrous peritoneal response with nodules in the peritoneum and formation of adhesions (Fig. 41). In the fixed fibrotic type, disease affecting omentum and mesentery extends into bowel serosa, with features of matted bowel loops on imaging. Omental masses and loculated ascites are often present [103, 105, 106].

Ultrasound is an important tool in the differential diagnosis between TB, non-TB infective ileitis and Crohn’s disease. Barium studies are the preferred imaging modality in the assessment of gastrointestinal TB, especially in low- and middle-income countries [107]. Early signs of disease include narrowing of the bowel lumen due to spasm and mucosal oedema, followed by wall thickening and wide gaping of the ileocecal valve with narrowing of the terminal ileum (Fleischner sign). Advanced disease shows focal or diffuse aphthous ulcers, usually bigger than in Crohn’s disease and typically in a linear or stellate orientation, following distribution of the lymphoid follicles; transverse in the colon and longitudinal in small bowel. CT remains an important imaging modality in the diagnosis of abdominal TB [102]. It is important in the assessment of the peritoneum and its reflections, mesentery and lymph nodes. It is often used in adults as the first imaging test, but it needs careful consideration in children due to the high radiation dose, and as such US is the first-line radiological investigation in children. On CT, in most patients, the ascitic fluid has high attenuation values (20–45 HU) due to the high protein and cellular contens. Up to 45% of cases show a lattice-like appearance due to multiple septations. Progression of disease is associated with developing fibrosis—the terminal ileum is narrow and fixed and the caecum is shortened, distorted in shape and rigid, causing displacement of the hepatic flexure inferiorly [107].

**Fig. 42:**
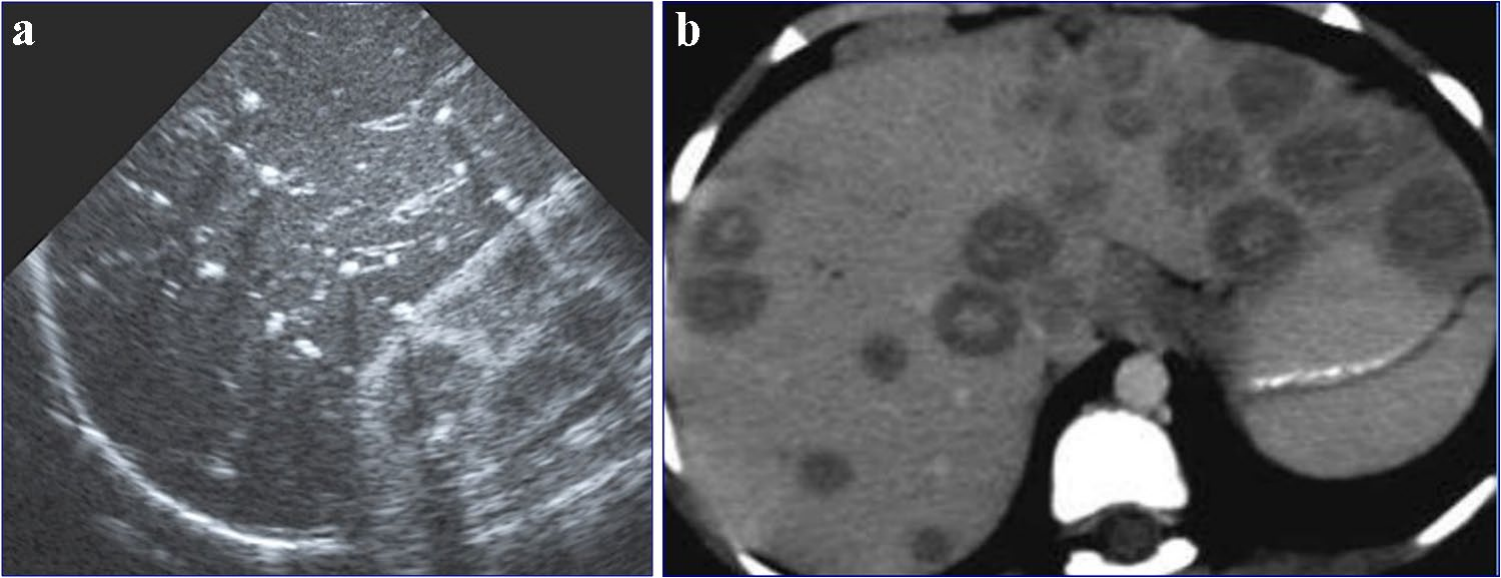
Hepatic tuberculosis (TB) in two children. **a** A transverse abdominal ultrasound in a 3-year-old boy reveals multiple small (3–5 mm) hyperechoic nodules, some of which exhibit posterior shadowing compatible with micronodular hepatic TB. **b** An axial contrast-enhanced computed tomography  abdominal image in a 7-year-old girl shows multiple round nodules measuring more than 10 mm. The lesions are low in attenuation with some showing central hyperattenuation. Some of the lesions in the left hepatic lobe coalesce and appear mass-like

Isolated involvement of solid abdominal organs is the least common manifestation of abdominal TB and is present in 10% to 15% of cases. It is caused by haematogenous spread and affects the genitourinary system, followed by the liver, spleen and pancreas [108] (Fig. 42).

**Fig. 43:**
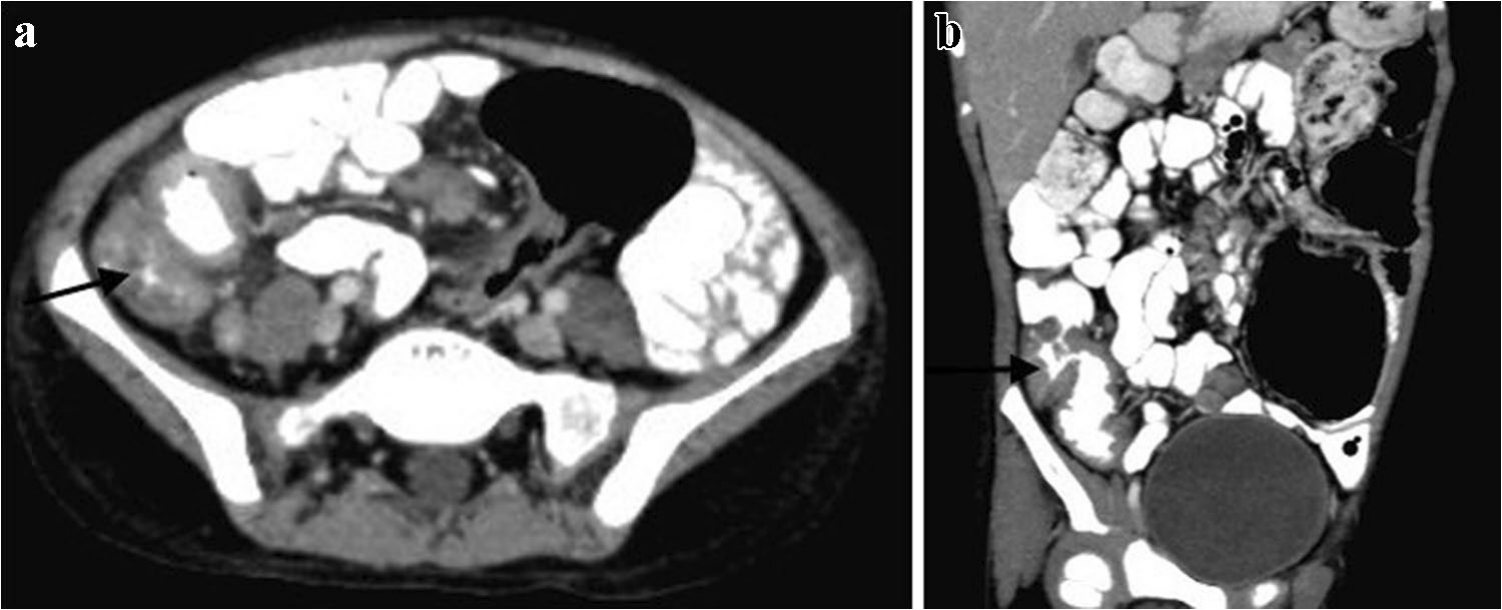
A 9-year-old boy with ileocecal tuberculosis. Axial (**a**) and coronal (**b**) contrast-enhanced computed tomography of the abdomen show circumferential mural thickening involving the caecum (*arrow* in **a**) and terminal ileum (*arrow *in **b**)

Abnormalities of the terminal ileum can be seen in up to 64% of patients with gastrointestinal TB and are often divided into ulcerative and ulcero-hypertrophic variants (Fig. 43). The progression of abdominal disease is often associated with hyperplastic bowel wall thickening, strictures and adhesions [108]. Peritoneal thickening and vivid enhancement can be seen in up to 100% of patients with TB ascites [109]. Abdominal TB can demonstrate multiple peritoneal nodules and irregular peritoneal thickening.

Mesenteric changes in abdominal TB include nodular lesions, mesenteric thickening and loss of normal configuration, with a typical ‘smudge’ appearance. Focal changes and fibrosis within the bowel wall may cause tethering of the adjacent mesentery and formation of stellate lesions.

CT is more superior than US in the assessment of TB lymphadenopathy, especially for the visualisation of deeper located abdominal lymph nodes. Various patterns of lymphadenopathy have been reported: increased number of normal size nodes, mildly enlarged scattered nodes, localised clusters of enlarged nodes and large conglomerate masses [107]. There are also different patterns of nodal enhancement. Peripheral enhancement is most common and reflects central liquefaction or caseous necrosis, with highly vascular inflammatory reaction in the perinodal areas. Involvement of solid abdominal organs and small and large bowel is easily detected with cross-sectional imaging. Recently, MRI has become more popular in imaging of abdominal TB [110]. Similar to CT, MRI can be used in the assessment of abdominal lymph nodes, solid organs, peritoneum, mesentery and small and large bowel. It is used in high-income countries to investigate non-specific abdominal pain and symptoms suggestive of inflammatory bowel disease in children due to its high contrast and spatial resolution and lack of ionising radiation. Standard MRI sequences, diffusion-weighted imaging and apparent diffusion coefficient images are especially helpful in the assessment of active infection and its response to treatment.

### Urogenital tuberculosis

Reportedly, nearly 20% of patients with TB have extrapulmonary TB, which is defined as infection of an organ or organs in addition to or other than the lungs [111, 112]. Urogenital TB, also referred to as genitourinary TB, is one of the common forms of extrapulmonary TB in adults and is reported to account for approximately 30% to 40% of all extrapulmonary TB cases [112, 113]. From 2% to 20% of patients with chest TB have concurrent genitourinary TB [113].

Urogenital TB affects the adult population between the second and fourth decades of life (with a mean age of 40 years) but is rare in children [114–116]. There is often a lengthy latent period (5–20 years, sometimes more than 20 years) between the original infection in the lungs and the subsequent appearance of clinical renal disease, which is the most likely reason that renal involvement is rare before the age of 20 years [114, 117]. The imaging findings described here are the classical findings described in adults.

In urogenital TB, the kidney is the most commonly involved site [112, 114, 117]. Diagnosis of renal TB and progression of disease can be detected by US, CT and MRI. Ultrasound is the initial imaging modality, while CT and MRI are the options for cross-sectional imaging [118].

**Fig. 44:**
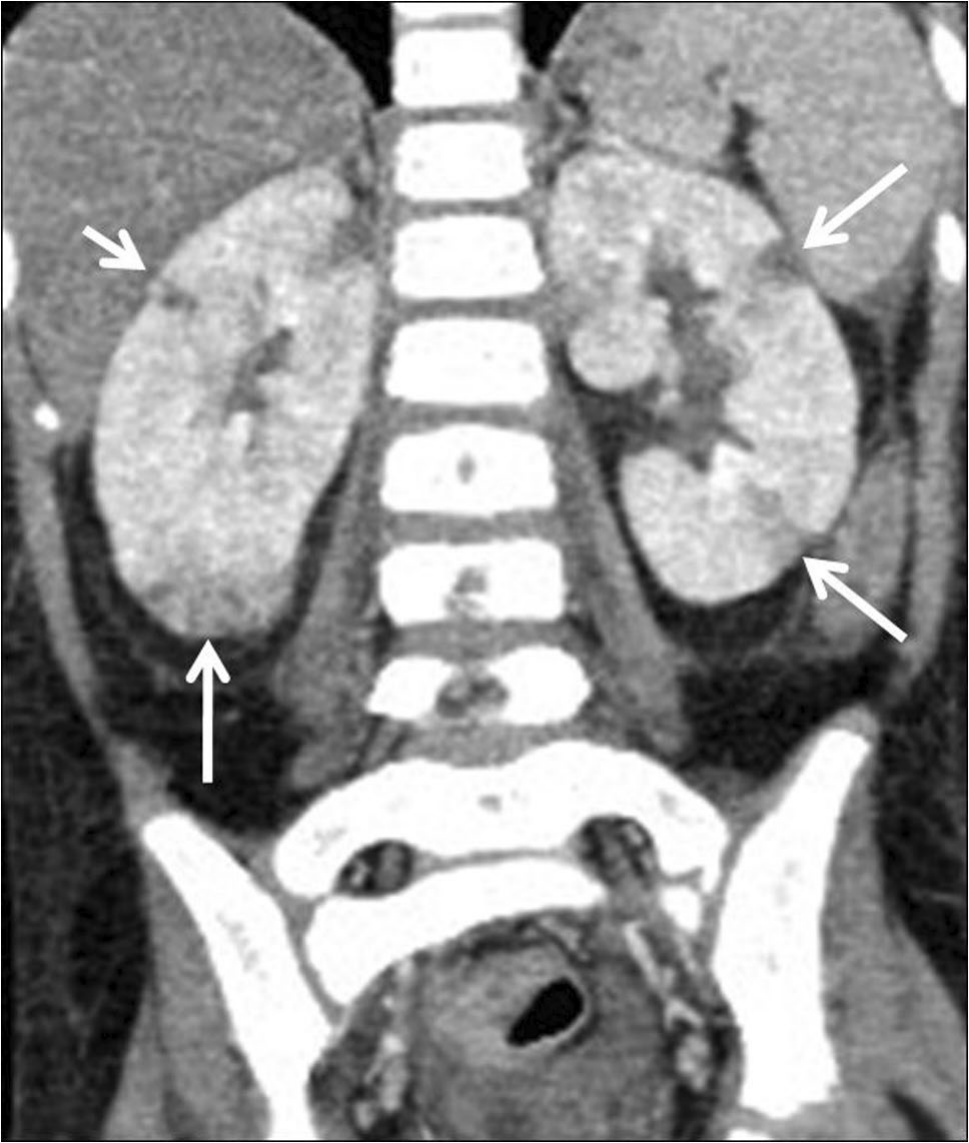
Tuberculous pyelonephritis in a 4-year-old boy. A coronal contrast-enhanced computed tomography image of the abdomen shows a striated nephrogram in the bilateral kidneys with wedge-shaped hypodense areas (*arrows*)

**Fig. 45:**
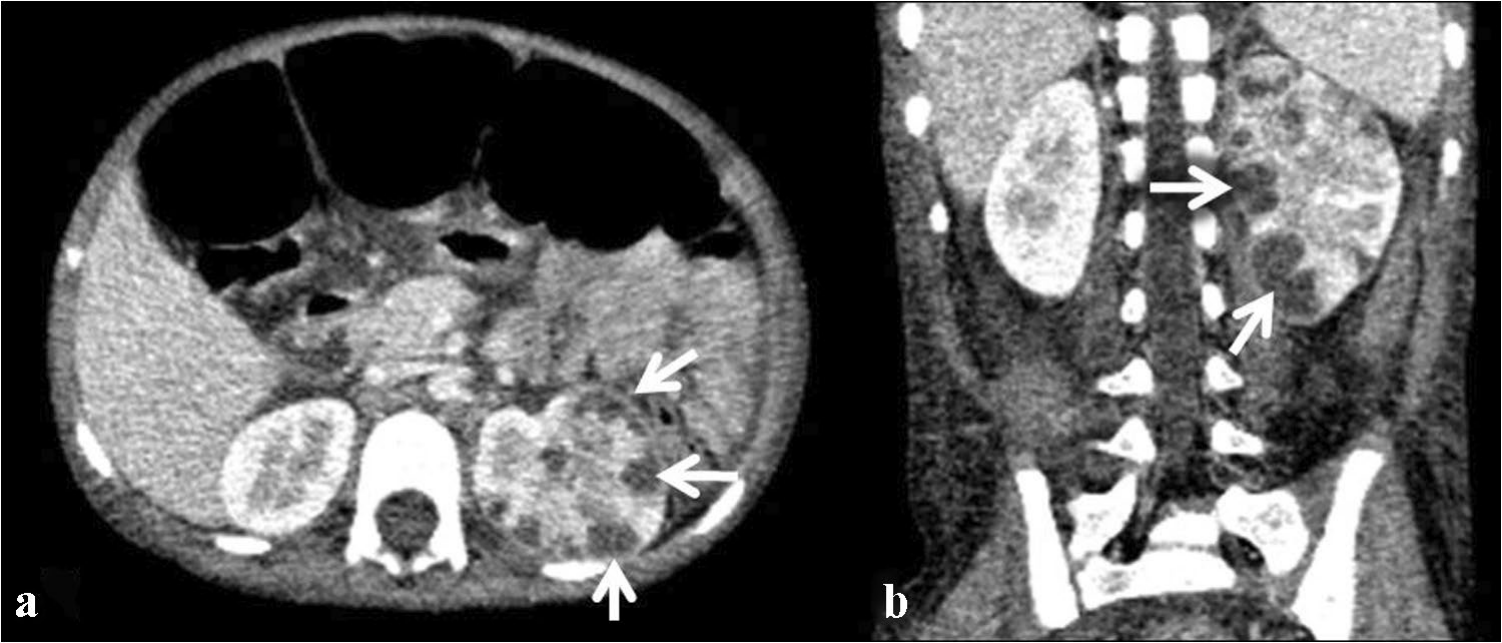
Tuberculous renal abscesses in a 5-year-old girl. Contrast-enhanced computed tomography images of the abdomen in axial (**a**) and coronal (**b**) planes show multiple peripherally enhancing hypodense lesions in the periphery of the left kidney (*arrows*)

Renal involvement in TB may be parenchymal or involve the pelvicalyceal system. Parenchymal involvement can be seen on CT and MRI as hypo-enhancing granulomas, striated nephrogram (Fig. 36), TB renal abscess formation (Figs. 44 and 45), tuberculomas and eventually autonephrectomy [112, 114, 118, 119]. The healing stage may demonstrate calcification, varying in appearance from punctate foci to almost complete replacement of the renal parenchyma, characteristically described as a putty kidney (during the autonephrectomy stage).

Peripheral lobar calcifications in the kidney represent yet another typical pattern that may be seen in end-stage kidney disease and is said to be characteristic of TB. A lobar pattern of calcification, in which a densely calcified rim is seen to outline the periphery of the distorted renal lobar anatomy, is also characteristic of renal TB [114, 118]. Pelvicalyceal system involvement can be seen at imaging as papillary necrosis, focal calyceal blunting/dilatation, infundibular and/or pelvic stenosis, peripheral urothelial calcification and urothelial thickening [112, 120].

Antegrade transit (urinary) of renal granuloma from the pelvicalyceal system can potentially seed into the ureters, bladder and even the urethra. Ureteral TB is said to develop concomitantly with renal TB in 50% of cases, and isolated ureteric TB without renal TB has not been described [117]. The most common sites of ureteric involvement are the distal third of the ureter, followed by the ureteropelvic junction [114]. Imaging findings of ureteric involvement in TB include ureteral thickening and enhancement, with periureteral stranding similar to that which may be seen with infectious urethritis [112].

Tuberculosis of the bladder, testes, vas deferens and epididymis can develop by means of retrograde spread from the prostate, in addition to lymphatic and haematogenous spread. Infection of the female genital tract has also been reported to develop from lymphatic or haematogenous spread from primary TB or secondary pulmonary TB [112, 118].

## Conclusion

Tuberculosis is one of the main causes of infectious disease in the chest and is associated with substantial morbidity and mortality in the paediatric population, particularly in low- and middle-income countries. In this review, we have described and illustrated the classic findings of childhood TB in each of the systems. Identifying the classic radiological features of paediatric TB is important for the individual management of patients and for a deeper understanding of the disease process. Repeat imaging provides valuable information on disease progression and resolution. Diagnosis of TB in paediatric patients in certain systems remains a clinical challenge given its vague presentation and lack of pathognomonic features. A high index of clinical suspicion and early recognition of classic imaging features are key to prompt diagnosis, initiation of treatment and prevention of long-term morbidity.

## References

[1] World Health Organisation Global Tuberculosis report 2021. Geneva. https://www.who.int/publications/i/item/9789240037021. Accessed Jan 2023

[2] Nachiappan AC, Rahbar K, Shi X (2017). Pulmonary tuberculosis: role of radiology in diagnosis and management. Radiographics.

[3] George R, Andronikou S, Theron S (2009). Pulmonary infections in HIV-positive children. Pediatr Radiol.

[4] Zar HJ (2008). Chronic lung disease in human immunodeficiency virus (HIV) infected children. Pediatr Pulmonol.

[5] Jeena PM, Coovadia HM, Thula SA (1998). Persistent and chronic lung disease in HIV-1 infected and uninfected African children. AIDS.

[6] Marais BJ, Gie RP, Schaaf HS (2006). Childhood pulmonary tuberculosis: old wisdom and new challenges. Am J Respir Crit Care Med.

[7] Zar HJ (2007). Diagnosis of pulmonary tuberculosis in children-what's new?. S Afr Med J.

[8] Marais BJ (2007). Childhood tuberculosis-risk assessment and diagnosis. S Afr Med J.

[9] Pitcher RD, Lombard CJ, Cotton MF (2015). Chest radiographic abnormalities in HIV-infected African children: a longitudinal study. Thorax.

[10] Andronikou S, van der Merwe DJ, Goussard P (2012). Usefulness of lateral radiographs for detecting tuberculous lymphadenopathy in children- confirmation using sagittal CT reconstruction with multiplanar cross-referencing. SA J Radiol.

[11] Sodhi KS, Bhalla AS, Mahomed N at al (2017) Imaging of thoracic tuberculosis in children: current and future directions. Pediatr Radiol 47:1260–1268 10.1007/s00247-017-3866-1 29052772

[12] Moseme T, Andronikou S (2014). Through the eye of the suprasternal notch: point-of-care sonography for tuberculous mediastinal lymphadenopathy in children. Pediatr Radiol.

[13] Leung AN, Muller NL, Pineda PR (1992). Primary tuberculosis in childhood: radiographic manifestations. Radiology.

[14] Andronikou S, Joseph E, Lucas S (2004). CT scanning for the detection of tuberculous mediastinal and hilar lymphadenopathy in children. Pediatr Radiol.

[15] Pitcher RD, Beningfield SJ, Zar HJ (2015). The chest X-ray features of chronic respiratory disease in HIV-infected children - a review. Paediatr Respir Rev.

[16] Weber AL, Bird KT, Janower ML (1968). Primary tuberculosis in childhood with particular emphasis o hanges affecting the tracheobronchial tree. Am J Roentgenol Radium Ther Nucl Med.

[17] Du Toit G, Swingler G, Iloni K (2002). Observer variation in detecting lymphadenopathy on chest radiography. Int J Tuberc Lung Dis.

[18] Kim WS, Moon WK, Kim IO (1997). Pulmonary tuberculosis in children: evaluation with CT. AJR Am J Roentgenol.

[19] Sharma SK, Mohan A (2017) Miliary tuberculosis. Microbiol Spectr 5. 10.1128/microbiolspec.TNMI7-0013-2016 10.1128/microbiolspec.tnmi7-0013-2016 PMC11687475 28281441

[20] Gurkan F, Bosnak M, Dikici B (1998). Miliary tuberculosis in children: a clinical review. Scand J Infect Dis.

[21] Hussey G, Chisholm T, Kibel M (1991). Miliary tuberculosis in children: a review of 94 cases. Pediatr Infect Dis J.

[22] Chang CW, Wu PW, Yeh CH (2018). Congenital tuberculosis: case report and review of the literature. Paediatr Int Child Health.

[23] Kim WS, Choi JI, Cheon JE (2006). Pulmonary tuberculosis in infants: radiographic and CT findings. AJR Am J Roentgenol.

[24] Kim HY, Song KS, Goo JM (2001). Thoracic sequelae and complications of tuberculosis. Radiographics.

[25] Rizzi EB, Schinina V, Cristofaro M et al (2011) Detection of pulmonary tuberculosis: comparing MR imaging with HRCT. BMC Infect Dis 11:243. 10.1186/1471-2334-11-243 10.1186/1471-2334-11-243 PMC3184086 21923910

[26] Peprah KO, Andronikou S, Goussard P (2012). Characteristic magnetic resonance imaging low T2 signal intensity of necrotic lung parenchyma in children with pulmonary tuberculosis. J Thorac Imaging.

[27] Yikilmaz A, Koc A, Coskun A (2011). Evaluation of pneumonia in children: comparison of MRI with fast imaging sequences at 1.5T with chest radiographs. Acta Radiol.

[28] Wong JS, Ng CS, Lee TW (2006). Bronchoscopic management of airway obstruction in pediatric endobronchial tuberculosis. Can Respir J.

[29] Lee EY, Restrepo R, Dillman JR (2012). Imaging evaluation of pediatric trachea and bronchi: systematic review and updates. Semin Roentgenol.

[30] Lee EY, Greenberg SB, Boiselle PM (2011). Multidetector computed tomography of pediatric large airway diseases: state-of-the-art. Radiol Clin North Am.

[31] Leung AN (1999). Pulmonary tuberculosis: the essentials. Radiology.

[32] Marais BJ, Gie RP, Schaaf HS (2004). The natural history of childhood intra-thoracic tuberculosis: a critical review of literature from the pre-chemotherapy era. Int J Tuberc Lung Dis.

[33] Moon WK, Kim WS, Kim IO (1999). Complicated pleural tuberculosis in children: CT evaluation. Pediatr Radiol.

[34] Marais BJ (2008). Tuberculosis in children. Pediatr Pulmonol.

[35] Hatipoğlu ON, Osma E, Manisali M (1996). High resolution computed tomographic findings in pulmonary tuberculosis. Thorax.

[36] Mahomed N, Reubenson G (2017) Immune reconstitution inflammatory syndrome in children. SA J Radiol 21:1257. 10.4102/sajr.v21i2.1257 10.4102/sajr.v21i2.1257 PMC6837832 31754484

[37] Van Rie A, Sawry S, Link-Gelles R (2015). Paradoxical tuberculosis-associated immune reconstitution inflammatory syndrome in children. Pediatr Pulmonol.

[38] Chiang SS, Khan FA, Milstein MB (2014). Treatment outcomes of childhood tuberculous meningitis: a systematic review and meta-analysis. Lancet Infect Dis.

[39] He Y, Han C, Chang KF et al (2017) Total delay in treatment among tuberculous meningitis patients in China: a retrospective cohort study. BMC Infect Dis 17:341. 10.1186/s12879-017-2447-0 10.1186/s12879-017-2447-0 PMC5429562 28499348

[40] Cresswell FV, Tugume L, Bahr NC (2020). Xpert MTB/RIF Ultra for the diagnosis of HIV-associated tuberculous meningitis: a prospective validation study. Lancet Infect Dis.

[41] Donovan J, Cresswell FV, Thuong NTT (2020). Xpert MTB/RIF ultra for the diagnosis of tuberculous meningitis: a small step forward. Clin Infect Dis.

[42] Andronikou S, Wilmshurst J, Hatherill M (2006). Distribution of brain infarction in children with tuberculous meningitis and correlation with outcome score at 6 months. Pediatr Radiol.

[43] Van der Merwe DJ, Andronikou S, Van Toorn R (2009). Brainstem ischemic lesions on MRI in children with tuberculous meningitis: with diffusion weighted confirmation. Childs Nerv Syst.

[44] Pienaar M, Andronikou S, Van Toorn R (2009). MRI to demonstrate diagnostic features and complications of TBM not seen with CT. Childs Nerv Syst.

[45] Omar N, Andronikou S, Van Toorn R (2011). Diffusion-weighted magnetic resonance imaging of borderzone necrosis in paediatric tuberculous meningitis. J Med Imaging Radiat Oncol.

[46] Theron S, Andronikou S, Grobbelaar M (2006). Localized basal meningeal enhancement in tuberculous meningitis. Pediatr Radiol.

[47] Andronikou S, Smith B, Hatherhill M (2004). Definitive neuroradiological diagnostic features of tuberculous meningitis in children. Pediatr Radiol.

[48] Przybojewski S, Andronikou S, Wilmshurst J (2006). Objective CT criteria to determine the presence of abnormal basal enhancement in children with suspected tuberculous meningitis. Pediatr Radiol.

[49] Bernaerts A, Vanhoenacker FM, Parizel PM (2003). Tuberculosis of the central nervous system: overview of neuroradiological findings. Eur Radiol.

[50] Andronikou S, Kilborn T (2016) Radiology of childhood tuberculosis. In: Handbook of child and adolescent tuberculosis. Oxford University Press, pp 109–146

[51] Dekker G, Andronikou S, Van Toorn R (2011). MRI findings in children with tuberculous meningitis: a comparison of HIV-infected and non-infected patients. Childs Nerv Syst.

[52] Marais S, Pepper DJ, Marais BJ (2010). HIV-associated tuberculous meningitis-diagnostic and therapeutic challenges. Tuberculosis (Edinb).

[53] Andronikou S, Govender N, Ramdass A (2012). MRI appearances of tuberculous meningitis in HIV-infected children: a paradoxically protective mechanism?. Imaging Med.

[54] Andronikou S, Wieselthaler N, Smith B (2005). Value of early follow-up CT in paediatric tuberculous meningitis. Pediatr Radiol.

[55] Ranjan P, Kalita J, Misra UK (2003). Serial study of clinical and CT changes in tuberculous meningitis. Neuroradiology.

[56] Figaji AA, Fieggen AG (2010). The neurosurgical and acute care management of tuberculous meningitis: evidence and current practice. Tuberculosis (Edinb).

[57] Van Toorn R (2015). Central nervous system in children. Handbook of child & adolescent tuberculosis.

[58] Bruwer GE, Van der Westhuizen S, Lombard CJ (2004). Can CT predict the level of CSF block in tuberculous hydrocephalus?. Childs Nerv Syst.

[59] Figaji AA, Fieggen AG, Peter JC (2003). Endoscopic third ventriculostomy in tuberculous meningitis. Childs Nerv Syst.

[60] Schoeman JF, Van Zyl LE, Laubscher JA (1995). Serial CT scanning in childhood tuberculous meningitis: prognostic features in 198 cases. J Child Neurol.

[61] Lammie GA, Hewlett RH, Schoeman JF (2009). Tuberculous cerebrovascular disease: a review. J Infect.

[62] Shukla R, Abbas A, Kumar P, Gupta RK (2008). Evaluation of cerebral infarction in tuberculous meningitis by diffusion weighted imaging. J Infect.

[63] Misra UK, Kalita J, Maurya PK (2011). Stroke in tuberculous meningitis. J Neurol Sci.

[64] Leiguarda R, Berthier M, Starkstein S el al (1988) Ischemic infarction in 25 children with tuberculous meningitis. Stroke 19:200–204 10.1161/01.str.19.2.200 3344536

[65] Rohlwink UK, Kilborn T, Wieselthaler N (2016). Imaging features of the brain, cerebral vessels and spine in pediatric tuberculous meningitis with associated hydrocephalus. Pediatr Infect Dis J.

[66] Andronikou S, Van Toorn R, Boerhout E (2009). MR imaging of the posterior hypophysis in children with tuberculous meningitis. Eur Radiol.

[67] Goyal M, Sharma A, Mishra NK (1997). Imaging appearance of pachymeningeal tuberculosis. AJR Am J Roentgenol.

[68] Huynh KK, Joshi SA, Brown EJ (2011). A delicate dance: host response to mycobacteria. Curr Opin Immunol.

[69] Trivedi R, Saksena S, Gupta RK (2009). Magnetic resonance imaging in central nervous system tuberculosis. Indian J Radiol Imaging.

[70] DeLance AR, Safaee M, Oh MC (2013). Tuberculoma of the central nervous system. J Clin Neurosci.

[71] Wasay M, Kheleani BA, Moolani MK (2003). Brain CT and MRI findings in 100 consecutive patients with intracranial tuberculoma. J Neuroimaging.

[72] Mishra R, Shrivastava A, Raj S, Chouksey P (2021). Intracranial tuberculomas and tubercular abscess- overcoming challenges. Neurology India.

[73] Luthra G, Parihar A, Nath K (2007). Comparative evaluation of fungal, tubercular, and pyogenic brain abscesses with conventional and diffusion MR imaging and proton MR spectroscopy. AJNR Am J Neuroradiol.

[74] Srivastava T, Kochar DK (2003). Asymptomatic spinal arachnoiditis in patients with tuberculous meningitis. Neuroradiology.

[75] Andronikou S, Jadwat S, Douis H (2002). Patterns of disease on MRI in 53 children with tuberculous spondylitis and the role of gadolinium. Pediatr Radiol.

[76] Gardam M, Lim S (2005). Mycobacterial osteomyelitis and arthritis. Infect Dis Clin North Am.

[77] Desai SR (1994). Early diagnosis of spinal tuberculosis by MRI. J Bone Joint Surg Br.

[78] Kilborn T, van Rensburg PJ, Candy S (2015). Pediatric and adult spinal tuberculosis: imaging and pathophysiology. Neuroimaging Clin N Am.

[79] Andronikou S, Bindapersad M, Govender N (2011). Musculoskeletal tuberculosis - imaging using low-end and advanced modalities for developing and developed countries. Acta Radiol.

[80] Chapman M, Murray RO, Stoker DJ (1979). Tuberculosis of the bones and joints. Semin Roentgenol.

[81] Teo HE, Peh WC (2004). Skeletal tuberculosis in children. Pediatr Radiol.

[82] Prasad A, Manchanda S, Sachdev N (2012). Imaging features of pediatric musculoskeletal tuberculosis. Pediatr Radiol.

[83] Gouliamos AD, Kehagias DT, Lahanis S (2001). MR imaging of tuberculous vertebral osteomyelitis: pictorial review. Eur Radiol.

[84] Polley P, Dunn R (2009). Noncontiguous spinal tuberculosis: incidence and management. Eur Spine J.

[85] Boxer DI, Pratt C, Hine AL at al (1992) Radiological features during and following treatment of spinal tuberculosis. Br J Radiol 65:476–479 10.1259/0007-1285-65-774-476 1628177

[86] Hoffman EB, Crosier JH, Cremin BJ (1993). Imaging in children with spinal tuberculosis. A comparison of radiography, computed tomography and magnetic resonance imaging. J Bone Joint Surg Br.

[87] Dunn R, Zondagh I, Candy S (2011). Spinal tuberculosis: magnetic resonance imaging and neurological impairment. Spine (Phila Pa 1976).

[88] De Backer AI, Vanhoenacker FM, Sanghvi DA (2009). Imaging features of extraaxial musculoskeletal tuberculosis. Indian J Radiol Imaging.

[89] De Backer AI, Mortele KJ, Vanhoenacker FM (2006). Imaging of extraspinal musculoskeletal tuberculosis. Eur J Radiol.

[90] Raut AA, Naphade PS, Ramakantan R (2016). Imaging spectrum of extrathoracic tuberculosis. Radiol Clin North Am.

[91] Hamijoyo L (2010). Tuberculous arthritis: an overview. Indonesian. Journal of Rheumatology.

[92] Hong SH, Kim SM, Ahn JM (2001). Tuberculous versus pyogenic arthritis: MR imaging evaluation. Radiology.

[93] Choi JA, Koh SH, Hong SH (2009). Rheumatoid arthritis and tuberculous arthritis: differentiating MRI features. AJR Am J Roentgenol.

[94] Kroot EJ, Hazes JM, Colin EM (2007). Poncet's disease: reactive arthritis accompanying tuberculosis. Two case reports and a review of the literature. Rheumatology (Oxford).

[95] Verma H, Rajvanshi N, Rathaur VK et al (2021) Poncet's disease: a case report. J Trop Pediatr 67:116 10.1093/tropej/fmaa116 33306806

[96] Rasool M (2009). Tuberculosis- the masquerader of bone lesions in children. SA Orthop J.

[97] Agarwal A, Khan SA, Qureshi NA (2011). Multifocal osteoarticular tuberculosis in children. J Orthop Surg (Hong Kong).

[98] Bhaskar KT, Bareh J (2013) Tuberculous dactylitis (spina ventosa) with concomitant ipsilateral axillary scrofuloderma in an immunocompetent child: A rare presentation of skeletal tuberculosis. Adv Biomed Res 2:29. 10.4103/2277-9175.107993 10.4103/2277-9175.107993 PMC3748632 23977657

[99] Ritz N, Connell TG, Tebruegge M (2011). Tuberculous dactylitis-an easily missed diagnosis. Eur J Clin Microbiol Infect Dis.

[100] Jaovisidha S, Chen C, Ryu KN (1996). Tuberculous tenosynovitis and bursitis: imaging findings in 21 cases. Radiology.

[101] Wang JY, Lee LN, Hsueh PR (2003). Tuberculous myositis: a rare but existing clinical entity. Rheumatology (Oxford).

[102] Akhan O, Pringot J (2002). Imaging of abdominal tuberculosis. Eur Radiol.

[103] Lal SB, Bolia R, Menon JV (2020). Abdominal tuberculosis in children: A real-world experience of 218 cases from an endemic region. JGH Open.

[104] Tinsa F, Essaddam L, Fitouri Z (2010). Abdominal tuberculosis in children. J Pediatr Gastroenterol Nutr.

[105] Delisle M, Seguin J, Zeilinski D (2016). Paediatric abdominal tuberculosis in developed countries: case series and literature review. Arch Dis Child.

[106] Malik R, Srivastava A, Yachha SK (2015). Childhood abdominal tuberculosis: Disease patterns, diagnosis, and drug resistance. Indian J Gastroenterol.

[107] Vanhoenacker FM, De Backer AI et al (2004) Imaging of gastrointestinal and abdominal tuberculosis. Eur Radiol 14(Suppl 3):E103–E115 10.1007/s00330-003-2047-9 14749955

[108] Debi U, Ravisankar V, Prasad KK (2014). Abdominal tuberculosis of the gastrointestinal tract: revisited. World J Gastroenterol.

[109] Ha HK, Jung JI, Lee MS (1996). CT differentiation of tuberculous peritonitis and peritoneal carcinomatosis. AJR Am J Roentgenol.

[110] De Backer AI, Mortelé KJ, Deeren D (2005). Abdominal tuberculous lymphadenopathy: MRI features. Eur Radiol.

[111] ECDC (2013) European centre for disease control and prevention. What is extrapulmonary TB?. https://www.ecdc.europa.eu/en/publications-data/third-external-evaluation-ecdc-2013-2017. Accessed Jan 2023

[112] Naeem M, Zulfiqar M, Siddiqui MA et al (2022) Imaging manifestations of genitourinary tuberculosis. Radiographics 42:E134. 10.1148/rg.229007 10.1148/rg.229007 35559663

[113] Figueiredo AA, Lucon AM, Junior RF (2008). Epidemiology of urogenital tuberculosis worldwide. Int J Urol.

[114] Merchant S, Bharati A, Merchant N (2013). Tuberculosis of the genitourinary system-Urinary tract tuberculosis: Renal tuberculosis-Part I. Indian J Radiol Imaging.

[115] Tonkin AK, Witten DM (1979). Genitourinary tuberculosis. Semin Roentgenol.

[116] Wise GJ (2009). Urinary tuberculosis: modern issues. Curr Urol Rep.

[117] Muneer A, Macrae B, Krishnamoorthy S (2019). Urogenital tuberculosis - epidemiology, pathogenesis and clinical features. Nat Rev Urol.

[118] Merchant S, Bharati A, Merchant N (2013). Tuberculosis of the genitourinary system-Urinary tract tuberculosis: Renal tuberculosis-Part II. Indian J Radiol Imaging.

[119] Gaudiano C, Tadolini M, Busato F (2017). Multidetector CT urography in urogenital tuberculosis: use of reformatted images for the assessment of the radiological findings. A pictorial essay. Abdom Radiol (NY).

[120] Mapukata A, Andronikou S, Fasulakis S (2007). Modern imaging of renal tuberculosis in children. Australas Radiol.

